# StuA-Regulated Processes in the Dermatophyte *Trichophyton rubrum*: Transcription Profile, Cell-Cell Adhesion, and Immunomodulation

**DOI:** 10.3389/fcimb.2021.643659

**Published:** 2021-06-08

**Authors:** Tamires A. Bitencourt, João Neves-da-Rocha, Maira P. Martins, Pablo R. Sanches, Elza A. S. Lang, Julio C. Bortolossi, Antonio Rossi, Nilce M. Martinez-Rossi

**Affiliations:** Department of Genetics, Ribeirão Preto Medical School, University of São Paulo, USP, Ribeirão Preto, Brazil

**Keywords:** APSES-family, biofilm, dermatophyte, metabolism, transcription factor, virulence, mycosis

## Abstract

Fungal infections represent a significant concern worldwide, contributing to human morbidity and mortality. Dermatophyte infections are among the most significant mycoses, and *Trichophyton rubrum* appears to be the principal causative agent. Thus, an understanding of its pathophysiology is urgently required. Several lines of evidence have demonstrated that the APSES family of transcription factors (Asm1p, Phd1p, Sok2p, Efg1p, and StuA) is an important point of vulnerability in fungal pathogens and a potential therapeutic target. These transcription factors are unique to fungi, contributing to cell differentiation and adaptation to environmental cues and virulence. It has recently been demonstrated that StuA plays a pleiotropic role in dermatophyte pathophysiology. It was suggested that it functions as a mediator of crosstalk between different pathways that ultimately contribute to adaptive responses and fungal-host interactions. The complex regulation of StuA and its interaction pathways are yet to be unveiled. Thus, this study aimed to gain a deeper understanding of StuA-regulated processes in *T. rubrum* by assessing global gene expression following growth on keratin or glucose sources. The data showed the involvement of StuA in biological processes related to central carbon metabolism and glycerol catabolism, reactive oxygen species metabolism, and cell wall construction. Changes in carbohydrate metabolism may be responsible for the significant alteration in cell wall pattern and consequently in cell-cell interaction and adhesion. Loss of StuA led to impaired biofilm production and promoted proinflammatory cytokine secretion in a human keratinocyte cell line. We also observed the StuA-dependent regulation of catalase genes. Altogether, these data demonstrate the multitude of regulatory targets of StuA with a critical role in central metabolism that may ultimately trigger a cascade of secondary effects with substantial impact on fungal physiology and virulence traits.

## Introduction

The interaction between fungi and their hosts involves a plethora of molecular pathways that synergize to sense and respond to changes in the surrounding milieu (Burmester et al., 2011; Peres et al., 2016; Martinez-Rossi et al., 2017). Hence, signal transduction plays an important role to enable cell responsiveness in a stimulus-specific manner. Fungal cells have the refined ability to discriminate environmental signals and tune their metabolism to deal with changing conditions. Transduction pathways are highly conserved between divergent fungal species due to their adaptive value from an evolutionary perspective (Lengeler et al., 2000). Moreover, a dynamic and synchronized fungal-host transcription program is activated to cope with different stimuli during fungal infection. Extracellular molecules can trigger alterations in the host immune response, impacting on the fungus metabolism and promoting cell structural modifications, thereby altering the infection outcome (Munoz et al., 2019). Altogether, invasion success strictly depends on a balance between virulence, responsiveness, and the ability to inhibit the host defense mechanisms.

Dermatophyte fungal infection consists of a coordinated process that involves adhesion, penetration, and colonization of keratinized tissues. Moreover, dermatophytoses are chronic, opportunistic diseases responsible for high morbidity, with both infection and treatment dramatically decreasing the quality of life of affected individuals (Sharma et al., 2017). Species from the genus *Trichophyton* are mainly responsible for causing cutaneous infections in humans, and *T. rubrum* is the most common clinical isolate worldwide (Seebacher et al., 2008; Brown et al., 2012; Almeida et al., 2019). Anthropophilic species modulate a mild inflammatory response, causing the infection to slowly and persistently progress (Peres et al., 2010). For this reason, a broader understanding of the various mechanisms involved in the interaction between dermatophytes and their hosts is crucial for the development of efficient therapeutic strategies. In particular, transcription factors are promising candidates for new antifungal treatments, given their complex role and involvement in many aspects of virulence and fungal adaptive strategies (Shelest, 2008).

The APSES family of transcription factors (Asm1p, Phd1p, Sok2p, Efg1p, and StuA) is a conserved class of proteins that occur exclusively in fungi, and are involved in the control of cell differentiation and development in ascomycetes. An important correlation between these factors and virulence has been observed in several phytopathogenic species (Nishimura et al., 2009; Lysoe et al., 2011; Pasquali et al., 2013), as well as in *Candida* species in which Efg1affects filamentation and subsequent biofilm formation (Ramage et al., 2002; Connolly et al., 2013). In *Aspergillus fumigatus*, StuA is involved in the regulation of morphogenesis and biosynthesis of secondary metabolites (Sheppard et al., 2005). Furthermore, null mutants of *stuA* in the dermatophyte *Anthroderma benhamiae* highlighted its role in keratin degradation and sexual development (Krober et al., 2017).

To further investigate the role of StuA in dermatophytes, a *stuA* knockout (Δ*stuA*) *T. rubrum* strain was previously constructed by our research group. This strain exhibited several macroscopic and microscopic morphological changes during growth in different conditions including keratin, and human skin and nail fragments (Lang et al., 2020). The study revealed that this transcription factor is directly involved in fungal morphogenesis, germination, conidiation, pigmentation, mycelial hydrophobicity, and susceptibility to different stressors. The strain also exhibited decreased activity of keratinolytic proteases, insufficient capacity to form aerial hyphae, and an overall loss of fitness.

Given what is already know about StuA, it is now crucial to have a thorough understanding of its function. In this study, RNA sequencing (RNA-seq) analysis of the Δ*stuA T. rubrum* strain was performed to assess changes in global gene expression promoted by the presence of keratin or glucose medium compared with wild-type fungus. This approach is expected to reveal significant changes in metabolism triggered by the lack of StuA. The potential for biofilm formation and interleukin secretion of the Δ*stuA* strain were also evaluated.

## Material and Methods

### Strains and Culture Conditions

*T. rubrum* wild-strain CBS 118892 was provided by the Westerdijk Fungal Biodiversity Institute (Utrecht, Netherlands). Its complete genomic sequence is publicly available at the Dermatophyte Comparative Database of the Broad Institute (ftp://ftp.broadinstitute.org/pub/annotation/fungi). The null mutant strain (Δ*stuA*) was constructed by replacing the wild *stuA* gene with an inactivation cassette containing *hph*, which confers resistance to hygromycin (Lang et al., 2020). The wild-type and mutant strains were initially grown on malt extract agar solid medium (2% glucose, 2% malt extract, 0.1% peptone, 2% agar, pH 5.7) at 28°C for 20 days. Approximately 1 × 10^6^ conidia of each strain were inoculated into 100 mL of Sabouraud Dextrose Broth. The cultures were maintained at 28°C for 96 h under constant agitation (120 rpm).

The resulting mycelia were washed with sterile water and transferred to 100 mL of minimal medium (Cove, 1966) containing 70 mM sodium nitrate (Sigma-Aldrich, St. Louis, MO, USA) and either 50 mM glucose (Sigma-Aldrich) or 0.5% bovine keratin (m/v). Cultures were incubated for 24, 48, or 96 h at 28°C under agitation. Biological material from three independent glucose or keratin replicates was filtered and stored at −80°C.

### RNA Extraction and Sequencing

Total RNA was extracted from mycelia using the Illustra RNAspin mini isolation kit (GE Healthcare, Chicago, IL, USA). The RNA concentration and quality were estimated using a NanoDrop ND-1000 spectrophotometer (Thermo Fisher Scientific, Waltham, MA, USA) and the Agilent 2100 Bioanalyzer (Agilent, Santa Clara, CA, USA). cDNA synthesis was carried out with the TruSeq RNA library Kit (Illumina, San Diego, CA, USA) according to the manufacturer’s protocol. cDNA libraries were sequenced on a NextSeq 500 system (Illumina), generating 150-bp paired-end reads.

### Data Analysis

Analysis of RNA sequencing (RNA-seq) data was performed as previously described (Martins et al., 2020). Briefly, a filter for quality control was applied through the FastQC tool, and Trimmomatic (Bolger et al., 2014) was used to remove adapters and Illumina-specific sequences. A cutoff threshold based on an average base quality score of 15 over a window of 4 bases was determined, and reads shorter than 36 bases in post-trimming were excluded. Then, the trimmed paired-end reads were aligned to the reference genome retrieved from Ensembl Fungi database (https://fungi.ensembl.org/Trichophyton_rubrum_cbs_118892_gca_000151425/Info/Index) using the STAR software (Dobin et al., 2013). Reads mapping to multiple locations were excluded using the STAR´s – out Filter Multimap N max 1 parameter, and gene-level read-counts were quantified using the STAR’s -quant Mode Gene Counts parameter. A visual inspection was carried out using the Integrative Genomics Viewer software (Thorvaldsdottir et al., 2013). Biological replicates were inspected through principal component analysis plots.

Differentially expressed genes (DEGs) were determined by comparing the RNA-seq data from wild-type strain, with the data obtained for the Δ*stuA* strain. The cultivation, RNA preparation, and sequencing of the wild-type and *stuA* strains were performed together. The RNA-seq data from the wild-type strain were used as the reference of gene modulation in these comparative analyses. The DESeq2 Bioconductor package (Love et al., 2014) was used to identify the DEGs. After that, the Benjamini-Hochberg correction (Benjamini and Hochberg, 1995) was applied (*P* < 0.05), and a cutoff threshold of ± 1.5 log2-fold change was set to reveal statistically significant expression differences. We conducted a functional categorization of DEGs according to gene ontology (GO) terms assigned by the Blast2GO algorithm. Lastly, the most representative categories for each experimental condition were identified by enrichment analysis using the BayGO algorithm (Vencio et al., 2006).

### Validation by Real-Time Quantitative Reverse Transcription Polymerase Chain Reaction (RT-qPCR)

We selected a set of genes according to their prominence in fold-change and their potential implications in processes regulated by the StuA factor. Quantification was performed using the StepOnePlus Real-Time PCR system (Applied Biosystems, Waltham, MA, USA), and reactions were prepared with SYBR Green and PCR Master Mix (Life Technologies, Waltham, MA, USA). We used ROX dye as a normalizer for the fluorescent signal, and the reference genes *rpb2* and *gapdh* served as internal controls (Jacob et al., 2012). The 2^−ΔΔCt^ relative quantification method was applied to calculate relative fold-changes. The oligonucleotide sequences, concentrations, and efficiency of each reaction are shown in **Supplementary Table S1**. All reactions were performed in triplicate.

### Cytokine Secretion in Co-Culture With Human Keratinocytes (HaCaT)

The co-culture assay was performed using 1 × 10^6^ conidia/mL and 2.5 × 10^5^ HaCaT cells/mL, which were incubated for 24 h at 37°C in 5% CO_2_, as previously described (Lang et al., 2020). To gain a better understanding of fungus–host interaction and the contribution of StuA to the chronicity of dermatophyte infections, the levels of interleukin (IL)-1β, IL-4, and interferon (INF)-*γ* in cell supernatants were assessed by enzyme-linked immunosorbent assay (Peprotech, Cranbury, NJ, USA) in accordance with the manufacturer’s recommendations.

### *In Vitro* Characterization of Biofilm Formation

The characterization of biofilm formation by *T. rubrum* strains was carried out as previously described (Costa-Orlandi et al., 2014), with slight modifications. Briefly, approximately 1 × 10^6^ conidia/mL obtained from 10-day-old plates of *T. rubrum* were seeded in 96-well plates, and incubated for 4 h for pre-adhesion. Then, the supernatant was removed and replaced by 200 µL of RPMI 1640 medium supplemented with L-glutamine and without sodium bicarbonate (Gibco), buffered with Mops (Sigma-Aldrich), and added with 2% of glucose (w/v).

The plates were incubated at 37°C without agitation for 24, 48, 72, 96, and 120 h. The metabolic activity of biofilms was determined using the XTT reduction assay (2.3-bis(2-methoxy-4-nitro-5-sulfophenyl)-5-[carbonyl (phenylamino)]-2H-tetrazoliumhydroxide). At the end of each incubation period, 50 μL of XTT solution (1 mg∙mL^−1^ in phosphate-buffered saline solution) plus 4 μL of menadione solution (1 mM in acetone) were added to each well followed by incubation at 37°C for 3 h. The colorimetric change in fungal biofilms, which correlates to cell viability, was determined using a microplate reader (Multiskan FC, Thermo Fisher Scientific) at 450 nm. The biomass of *T. rubrum*-derived biofilms was measured by crystal violet staining after 96 h. The culture medium was removed from each well and adhered cells were washed thrice with phosphate-buffered saline solution, then the wells were allowed to dry at room temperature for about 1 min. Subsequently, 100 μL of 0.5% crystal violet solution was added to each well for 5 min. The wells were then washed four times with sterile water, followed by decolorization by the addition of 100 μL of 95% ethanol solution. The plates were analyzed using a microplate reader (Multiskan FC, Thermo Fisher Scientific) at a wavelength of 550 nm.

### Statistical Analyses

Statistical significance was evaluated by the unpaired Student’s *t*-test or one-way analysis of variance (ANOVA) followed by Tukey’s post-hoc test. The Pearson correlation coefficient was determined for the RT-qPCR and RNA-seq fold-change values. Prism v. 5.1 (GraphPad Software, San Diego, CA, USA) was used for the statistical analyses and to generate the graphs.

## Results

### Transcriptional Profile of the Δ*stuA* Mutant in the Presence of Glucose or Keratin

RNA-seq data revealed the global transcriptional changes in *T. rubrum* after deletion of the *stuA* and growth in glucose or keratin medium for three different time points. Next-generation sequencing generated 7,889,656 and 13,990,682 high-quality reads for each condition, which resulted in approximately 7,482,678 and 12,889,091 mapped paired-end sequences (**Supplementary Table S2**). Upregulated and downregulated transcripts were defined by a 1.5-fold change cutoff and a stringent statistical significance threshold of *P* < 0.05.

Comparison of the Δ*stuA T. rubrum* mutant and the wild-type strain (control) revealed 481, 749, and 503 genes modulated upon 24, 48, and 96 h, respectively, in the presence of glucose. As for keratin, 460, 689, 798 genes were modulated at 24, 48, and 96 h, respectively. A total of 1,170 DEGs were identified in samples grown in glucose and 1,230 DEGs in samples grown with keratin. Among these genes, 134 and 209 were modulated in the Δ*stuA* strain in a time-independent manner in response to glucose and keratin, respectively; considering only the three times analyzed.

**Figure 1:**
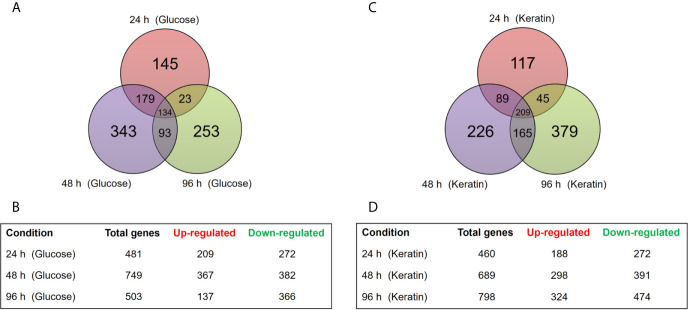
Time-dependent distribution of differentially expressed genes (DEGs) in the Δ*stuA* strain compared with that in the wild-type during growth on glucose or keratin sources. **(A)** Venn diagram illustrates the number of transcripts modulated in Δ*stuA T. rubrum* grown in glucose medium for 24, 48, or 96 h compared to wild-type. **(B)** Number of DEGs at each time point during Δ*stuA T. rubrum* growth in glucose. **(C)** Venn diagram illustrates the number of transcripts modulated at each time point in Δ*stuA T. rubrum* grown in keratin-supplemented medium. **(D)** Number of DEGs in Δ*stuA T. rubrum* grown on keratin for 24, 48, or 96 h.

As expected, most of the observed changes were time-dependent. Notably, the greatest number of DEGs was observed at 48 h of culture in glucose and at 96 h of culture with keratin. A previous study reported that dermatophyte growth on keratin triggers an alkalization of the culture medium at 72−96 h, which corresponds to high proteolysis activity and probably correlates with the initiation of the dermatophyte infection process (Maranhão et al., 2007). **Figure 1** schematically shows the distribution of these data.

A survey of the top 10 most significantly modulated genes (**Supplementary Tables S3**, **S4**) revealed several transcripts with undefined molecular functions, most of which were exclusive to one single experimental condition. Thus, it seems that future improvements in the annotation of *T. rubrum* and of other related species it is still needed for a better understanding of root processes, such as the ones studied here. Nonetheless, critical metabolic pathways, including the reactive oxygen species (ROS) metabolism, cell wall organization, and genes involved in protein folding and proteostasis, seemed highly relevant in this analysis. The complete DEGs list for each culture condition is shown in **Supplementary Table S5**.

### Functional Categorization of DEGs

Following transcriptome analyses, the identified DEGs were further characterized to determine the main metabolic pathways and processes to which they are associated. This approach was expected to precisely define the StuA regulator activity implicated in these particular molecular contexts. Thus, the Blast2Go tool (Conesa et al., 2005) was used to assess the distribution of DEGs as an effect of the presence of the Δ*stuA* mutation.

**Figure 2:**
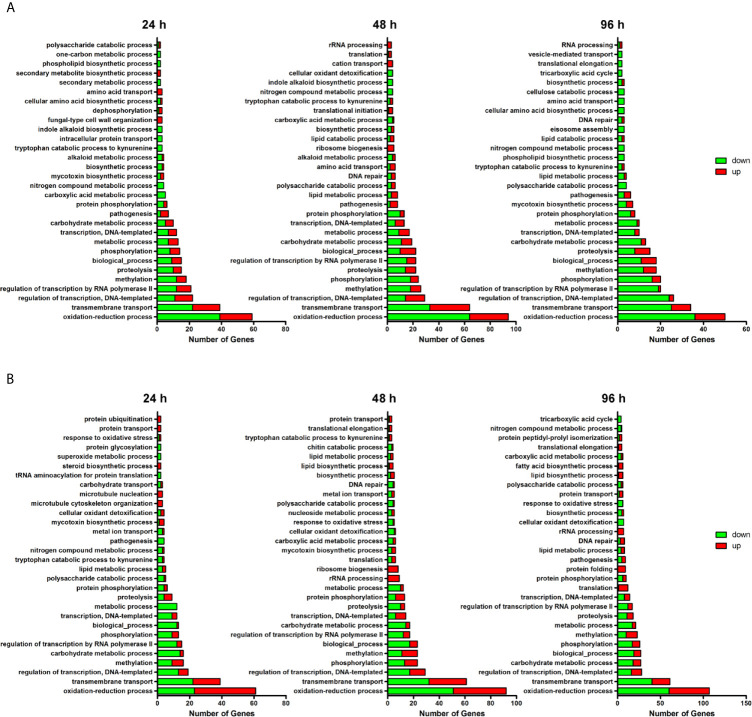
A summary of the Gene Ontology (GO) terms related to biological processes (BP) classification. Red and green bars represent up- and down-regulated genes, respectively. Categories determined for DEGs in Δ*stuA T. rubrum* strain during its growth in **(A)** glucose- and **(B)** keratin-supplemented medium at each time point.

A summary of main GO terms is hierarchically presented in **Figure 2**, and the full list of the enriched GO terms is shown in **Supplementary Table S6**. Carbohydrate metabolism, phosphorylation, oxidation-reduction, and regulation of transcription, microtubule nucleation, and chitin processes, were among the processes associated with the identified DEGs. Moreover, these main classes comprised genes involved in biological processes related to cell cycle, carbon metabolism, ROS metabolism, phosphorylation, and cell wall construction (**Table 1**).

**Table 1:** Differentially expressed genes belonging to some gene ontology terms – metabolism, oxidative stress response, phosphorylation,and reproduction – in *ΔstuA* mutant vs. wild‐type *Trichophyton rubrum*.

ID/Gene Product Name	Glucose			Keratin		
	24h	48h	96h	24h	48h	96h
**GO:0005975 - carbohydrate metabolic process**						
TERG_03052 - glycosyl hydrolase	1.65	2.41				1.99
TERG_03054 - glycosyl hydrolase	2.20	2.66				1.93
TERG_03223 - N-acetylglucosamine-6-phosphate deacetylase				-3.10	-3.04	
TERG_03226 - glucosamine-6-phosphate deaminase				-1.94	-2.08	
TERG_03229 - hexokinase					-1.65	-1.77
TERG_05576 - cell wall glucanase			-1.61			
TERG_05625 - class V chitinase				-2.51	-2.13	-1.60
TERG_06189 - endo-1,3(4)-beta-glucanase		-2.32	-1.99			
TERG_06242 - glucanase				-3.67		-3.14
TERG_06638 - endochitinase		-2.05				
TERG_06929 - chitinase		1.77		-2.63	-3.78	-2.11
TERG_08459 - cell wall glucanase		-1.71	-2.19			
TERG_12107 - 1,4-alpha-glucan-branching enzyme						1.98
**GO:0006072 - glycerol-3-phosphate metabolic process**						
TERG_01901 - glycerol kinase		-2.10	-2.99	2.00	2.68	2.13
**GO:0006096 - glycolytic process**						
TERG_07235 - fructose-1,6-bisphosphatase	-1.64					
TERG_01281 - malate synthase, glyoxysomal		-2.33	-2.12			-2.07
TERG_11639 - isocitrate lyase					-1.90	-2.50
**GO:0006099 - tricarboxylic acid cycle**						
TERG_01272 - 2-methylcitrate synthase				-1.54	-1.85	-1.58
**GO:0006164 - purine nucleotide biosynthetic process**						
TERG_01446 - GMP synthase [glutamine-hydrolyzing]					1.64	2.01
**GO:0006189 - 'de novo' IMP biosynthetic process**						
TERG_06128 - phosphoribosylglycinamide formyltransferase			2.10			
**GO:0006190 - inosine salvage**						
TERG_05439 - IMP-specific 5'-nucleotidase				-1.59	-1.61	-1.98
**GO:0016310 - phosphorylation**						
TERG_00119 - CAMK/CAMKL/GIN4 protein kinase	1.70	2.00	1.61	2.03	3.95	
TERG_06366 - CAMK protein kinase			-1.68			
TERG_06761 - CAMK protein kinase	1.95					
TERG_03544 - CMGC/RCK/MAK protein kinase	-1.70	-1.74	-3.13	-1.84		-1.55
TERG_04058 - STE/STE11 protein kinase						1.60
TERG_00315 - RAN protein kinase			-1.88	-1.60		
TERG_08066 - STE/STE7 protein kinase					1.51	1.84
TERG_04731 - STE/STE20 protein kinase					1.61	
TERG_00694 - glutamate 5-kinase			-1.88	2.07	1.87	2.85
**GO:0006537 - glutamate biosynthetic process**						
TERG_06509 - glutamate synthase				1.66		
**GO:0006807 - nitrogen compound metabolic process**						
TERG_02133 - fluG protein	-4.68	-3.92	-3.91	-2.42		
TERG_02368 - extracellular developmental signal biosynthesis protein FluG	-2.02	-2.53	-1.98	-1.54	-1.95	-3.15
**GO:0006979 - response to oxidative stress**						
TERG_01463 - cytochrome c peroxidase					-1.99	-2.06
TERG_02005 - catalase		-1.88				
TERG_06053 - catalase		-2.51				-2.32
**GO:0019430 - removal of superoxide radicals**						
TERG_08969 - cytosolic Cu/Zn superoxide dismutase				-2.58	-2.33	-3.17
**GO:0030437 - ascospore formation**						
TERG_03336 - Sec14 cytosolic factor						1.83
**GO:0032220 - plasma membrane fusion involved in cytogamy**						
TERG_08778 - hypothetical protein		5.11				
**GO:0045132 - meiotic chromosome segregation**						
TERG_02489 - sister chromatid cohesion acetyltransferase Eco1		-1.72	-1.70			
**GO:0000079 - regulation of cyclin-dependent protein serine/threonine kinase activity**						
TERG_04793 - cyclin				-1.67	-1.51	-1.60

### Validation of Transcriptome Data by RT-qPCR

**Figure 3:**
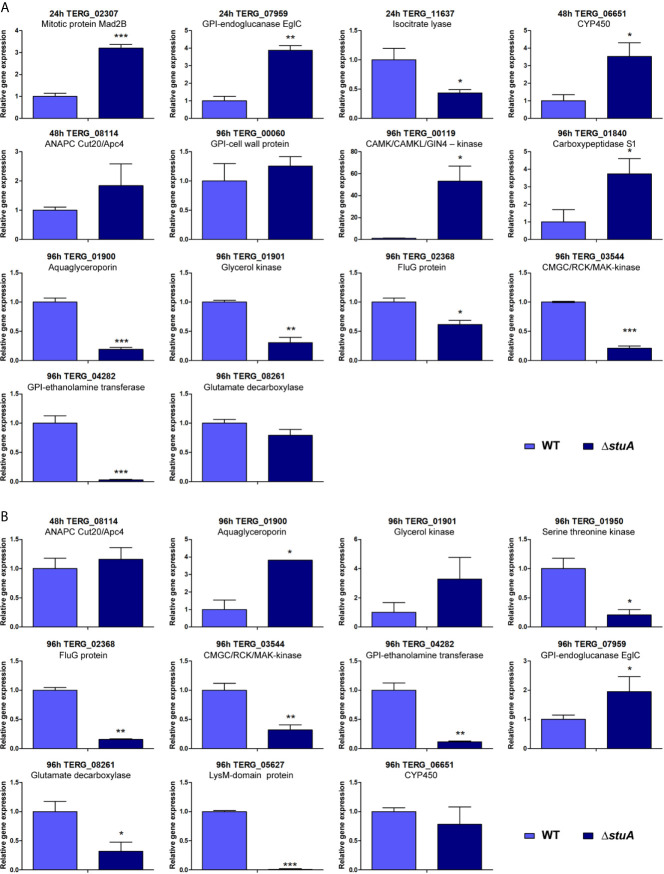
Panel of genes validated by RT-qPCR. Transcript levels are represented as fold-change values at each time point in the Δ*stuA* strain during grown in **(A)** glucose- and **(B)** keratin-supplemented medium relative to control (wild-type strain). Statistical significance was determined using an unpaired *t*-test (*P < 0.05; **P < 0.01; ***P < 0.001).

Next, potential StuA-regulated genes were strategically chosen to validate the RNA-seq data. Therefore, transcripts modulated under different conditions were selected and their individual expression was assessed. RT-qPCR analysis was carried out for 16 genes related to processes such as lipid, carbohydrate, and amino acid metabolism; cell wall organization; membrane transport; signal transduction; cell cycle regulation; fungal resistance; and pathogenesis. Expression profiles are shown in **Figure 3**, and an overview of these results is displayed in **Supplementary Table S7**. A positive correlation between the RNA-seq and RT-qPCR values was observed (Pearson’s correlation, *r* ≥ 0.70, *P* < 0.001).

### StuA Affects Cell-Cell Adhesion

**Figure 4:**
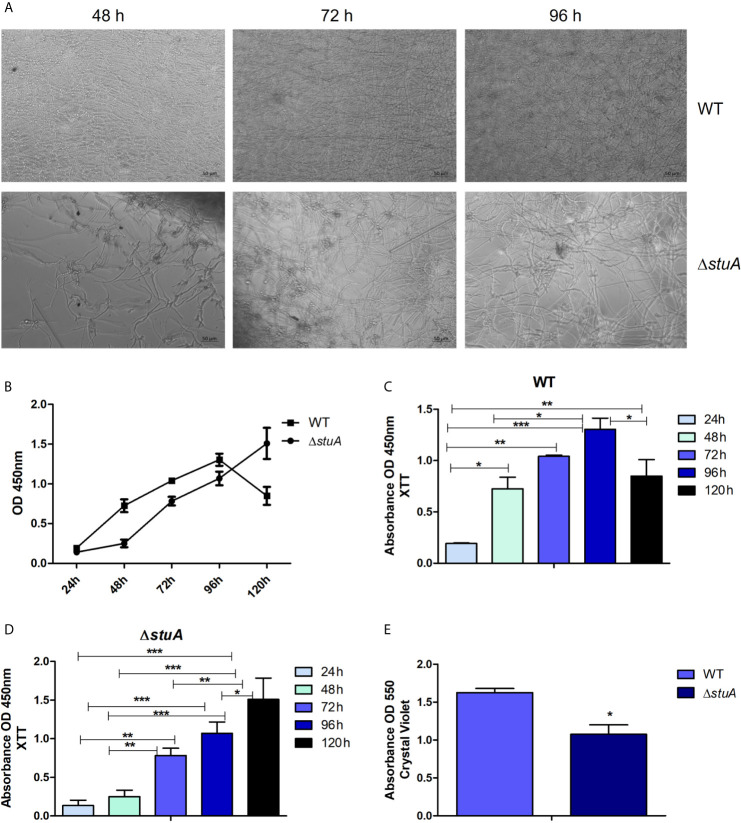
StuA supports cell-cell adhesion. **(A)** Morphology and **(B)** comparative kinetics of biofilm formation in Δ*stuA* and wild-type strains of *T. rubrum.* Metabolic activity of the biofilm produced by **(C)** wild-type and **(D)** Δ*stuA* strains of *T. rubrum* at each time point. Statistical significance was determined using ANOVA followed by Tukey’s post-hoc test (*P < 0.05; **P < 0.01; ***P < 0.001). **(E)** Biomass production in Δ*stuA* and wild-type strains of *T. rubrum* determined at 96 h by crystal violet staining. Statistical significance was determined by unpaired T-test (*P < 0.05).

Given the involvement of StuA on cell wall organization and cell wall in interactions with host tissues, the biofilm formation by the Δ*stuA* strain were further characterized. Biofilms are heterogeneous structures surrounded by an extracellular matrix. The formation of biofilms is associated with a set of advantages such as protection against hostile environments, microbial communication, gene expression regulation, and improvement in nutrition, growth, and excretion (Percival et al., 2012; Costa-Orlandi et al., 2017; de Barros et al., 2020). The data revealed that Δ*stuA* mutant had a different kinetic of mature biofilm formation and marked differences in hyphal development compared to wild-type *T. rubrum* (**Figures 4A, B**). In the wild-type strain, a significant increase in metabolic activity was observed at 72 h, with a slight increase at 96 h, followed by an activity decay at 120 h (**Figure 4C**), consistent with previous data (Garcia et al., 2020). In contrast, in the Δ*stuA* strain, an increase in metabolic activity was only detected at 120 h (**Figure 4D**). Moreover, the biomass production at 96 h was significantly reduced in the mutant strain (**Figure 4E**). This finding agreed with the metabolic activity data determined by XTT assay (**Figures 4C, D**).

### *stuA* Deletion Induces a Proinflammatory Response of Keratinocytes

**Figure 5:**
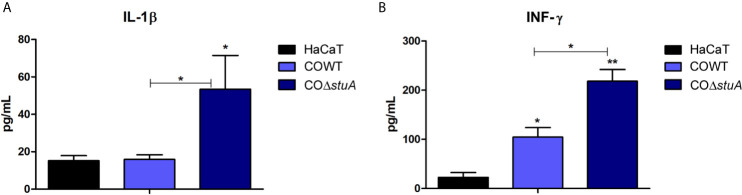
*stuA* deletion induces a proinflammatory profile in human keratinocytes. Levels of pro-inflammatory cytokines produced by HaCaT cells after coculture with conidia from wild-type and Δ*stuA* strains of *T.* rubrum for 24 h. Comparison of **(A)** interleukin (IL)-1β and **(B)** interferon (INF)-γ secretion induced by each strain. Statistical significance was determined using ANOVA followed by Tukey’s post-hoc test (*P < 0.05; **P < 0.01).

Lastly, we evaluated the impact of *stuA* deletion on the cell wall composition regarding the host immune response. Conceivably, the components of *T. rubrum* cell wall interact with keratinocytes and, due to this interplay, a set of cytokines is released as an inflammatory response by host cells. HaCaT cells were incubated with conidia cells from wild-type and Δ*stuA* strains for 24 h. The culture supernatants were collected, and cytokine production was assessed. The levels of IL-1β and IFN-γ were evaluated, and a pronounced increase in proinflammatory cytokine production was observed in the Δ*stuA* strain (**Figure 5**). In turn, similarly low levels of the anti-inflammatory cytokine IL-4 was observed in both strains (data not shown).

## Discussion

Under the lens of high-throughput approaches, it is possible to uncover some of the mechanisms governed by transcription factors. In this work, high-throughput RNA-seq was performed to unveil the regulatory role of StuA in *T. rubrum*. Our data revealed that deletion of *stuA* triggers a series of stress response events involving the central carbon metabolism, glycerol catabolism, ROS metabolism, and cell wall remodeling.

Previous work has demonstrated the involvement of StuA in central carbon metabolism related to glycolysis, tricarboxylic acid cycle, and amino acid synthesis, and reported slower growth of the mutant strain on glucose, sucrose, and glycerol. In contrast, supplementation of media with glutamate restored growth rates (IpCho et al., 2010). Thus, it is reasonable to hypothesize that glutamate metabolism might work as a bypass that leads to the production of osmolytes such as glycerol in Δ*stuA* mutant strains. Our data confirmed the role of StuA in central carbon metabolism and showed a trend towards upregulation of glutamate metabolism (TERG_00694 and TERG_06509) when the strain was grown on keratin sources. Increased glycerol catabolism was observed in the Δ*stuA T. rubrum* strain growth on keratin, whereas reduced glycerol catabolism was observed in glucose-supplemented culture conditions (**Table 1**). Previously, it has been shown that the growth of wild-type and Δ*stuA* strains from *T. rubrum* in liquid keratin media generated equivalent biomass. Simultaneously, the mutant strain showed a considerable reduction in keratinolytic activity (Lang et al., 2020). Therefore, it is conceivable that a shift in an alternative metabolism route with the activation of glycerol catabolism occurred as an adaptation to the change in preferred sources of carbon and energy, thereby buffering the stress caused by gene deletion. Pathogenic microorganisms have demonstrated the ability to adjust their metabolism according to their surrounding milieu by activating glycerol metabolism, a specific adaptation that was deemed as pathometabolism (Eisenreich et al., 2015; Blotz and Stulke, 2017). Here, we showed that dermatophytes can exhibit this metabolic adaptation mechanism, which could be another survival strategy in the host.

The present data also suggests that the impact of the Δ*stuA* mutation on central carbon metabolism and osmolyte synthesis may be responsible for secondary effects in fungal cells. Indeed, a recent study suggested the involvement of StuA in osmoregulation, with impact on cell wall construction and conidia germination (Lang et al., 2020). The consumption of osmolytes is closely related to conidial germination and viability (Hallsworth and Magan, 1995), as they are responsible for generating a driving force for conidial development that ultimately controls the turgor pressure to counteract the rigid cell wall barrier (Foster et al., 2017). Crosstalk between some pathways is evidenced during these processes, mainly represented by the mitogen-activated protein kinase (MAPK) cell wall integrity and high-osmolarity glycerol (Hog1) pathways (Hohmann, 2009). Indeed, the present data suggested that some genes belonging to these pathways appeared to be regulated by StuA (GO:0016310 – phosphorylation), such as genes encoding serine/threonine-protein kinases -STE family (TERG_08066; TERG_04058; TERG_04731), and calmodulin-dependent kinases (CAMK; TERG_06366; TERG_06761), as shown in **Table 1**. Another line of evidence suggests that MAPK and CAMK are associated with conidial germination, fungal development, and ROS metabolism (Riquelme et al., 2018).

It has also been demonstrated that Δ*stuA* mutant strains in species such as *A. fumigatus* and *T. rubrum* have a rapid germination rate and low conidiation production (Sheppard et al., 2005; Lang et al., 2020). In microbes, growth rate and conidia germination are used to determine stress status. It is assumed that the optimal growth rate might trigger lower cellular resilience, with little competitive ability. In contrast, a rapid germination rate stretches the metabolism too far and can impact on cell function and structure, mainly due to increased ROS production (Hallsworth, 2018). Previously, a reduction in conidiation was demonstrated in an Δ*stuA Aspergillus nidulans* mutant (Miller et al., 1992; Melin et al., 2003), suggesting a cooperative relationship between *stuA*, bristle (*brlA*), and abacus (*abaA*) for asexual development (Miller et al., 1992). It has been demonstrated that StuA is responsible for regulating the abaA-brlA pathway in *A. nidulan*s, indicated by the presence of genes that display StuAp response elements (StREs) upstream these regulatory developmental pathways (Dutton et al., 1997). Thus, we verified that genes encoding regulators of cell division, such as ascospore- and cyclin-related genes, were mainly upregulated in the Δ*stuA T. rubrum* strain. In contrast, genes involved in the asexual reproduction FluG-pathway (TERG_02133 and TERG_02368) were decreased in the Δ*stuA T. rubrum* strain (**Table 1**).

In addition, RNA-seq analyses revealed a reduction in the transcript levels of catalase genes in Δ*stuA T. rubrum* strain (TERG_06053, TERG_02005), suggesting a StuA-dependent regulation of catalase genes (**Table 1**). This finding agrees with other reports that showed the involvement of StuA regulation in catalase expression and an increase in susceptibility to oxidative stress (Scherer et al., 2002; Sheppard et al., 2005).

The Δ*stuA T. rubrum* strain presented hyphae thickness, increased conidia size, hypertrophy, and structures similar to that of chlamydoconidia, unlike the wild-type strain (Lang et al., 2020). In addition, the conidial wall thickness of the mutant strain was about 1.6 times greater than that of the wild-type strain (results not shown). Here, we showed that this mutant presented differently expressed genes encoding chitinases, glucanases, and cell wall integrity-related genes (**Table 1**). In addition, a LysM-domain protein was downregulated in our *stuA* mutant strain. Similarly, a previous report also showed downregulation of a LysM-encoding protein in a strain harboring another mutated APSES transcription factor (Sarmiento-Villamil et al., 2018). Fungal LysM-containing proteins contribute to host immune evasion by damping the free fungal chitin molecules (de Jonge et al., 2010). In addition, a previous study also suggested the involvement of LysM-domain genes in dermatophytes degradation of keratin (Lopes et al., 2019).

Given the relationship between cell wall composition and immune response, we sought to investigate the inflammatory response triggered by the Δ*stuA* mutant strain. Our data showed a shift in HaCaT cells towards a proinflammatory profile upon coculture with the mutant strain compared with the wild-type strain. These findings suggest a role for StuA in immune evasion and/or chronicity of skin fungal infections. Noteworthy, the faster germination rate of the Δ*stuA* mutant strain, as previously shown (Lang et al., 2020), might also be related to a more robust proinflammatory response and consequent fungal clearance (Rosowski et al., 2018). Spore germination is followed by cell wall remodeling. The altered cell wall composition could also impact on the adhesion of conidia/hyphae to other surfaces or fungal cells, as previously demonstrated for a null *sitA* strain of *A. fumigatus*, which identified considerable differences in cell wall surface proteins (Bom et al., 2015). Moreover, a previous report showed the involvement of Efg1 and C2H2 transcription factors, amino acid metabolism, and cell wall genes in the biofilm environment (Fanning and Mitchell, 2012). This study demonstrated that more than 50% of cell wall genes from *A. fumigatus* are changed in the biofilm habitat, which are expected to be related to adherence properties and sensory for adherence-induced responses. Among the surface genes potentially involved in the biofilm matrix are included those encoding the beta-glucan and polysaccharide, hydrophobins, and adhesins. (Fanning and Mitchell, 2012). Herein, we showed that deletion of *stuA* downregulated the genes coding the 1,3-beta-glucan synthase component FKS1, cell wall serine-threonine-rich galactomannnoprotein Mp1, and a putative hydrophobin (**Table S5**). Moreover, genes encoding C2H2 zinc finger domain-containing proteins were differentially modulated in the Δ*stuA* strain (**Table S5**).

**Figure 6:**
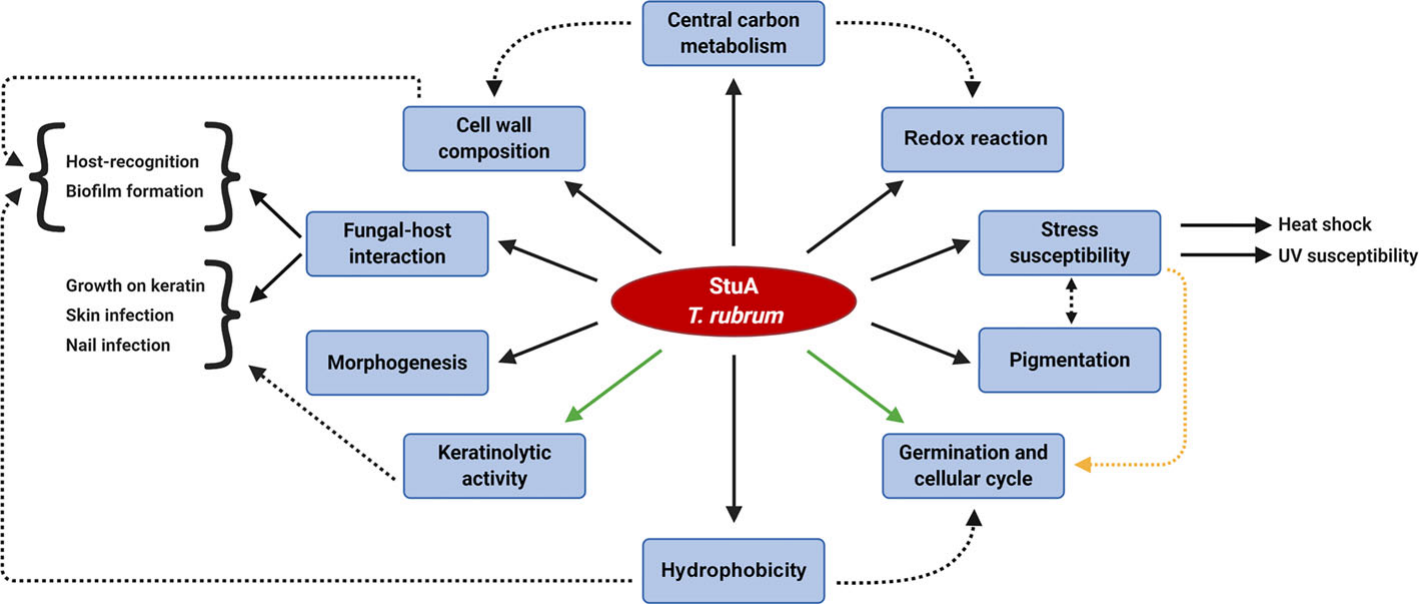
Schematic overview of the biological processes potentially regulated by StuA in the dermatophyte *Trichophyton rubrum*. The regulation of central carbon metabolism is cross-related to other StuA targeted-processes. Dashed arrows indicate a correlation between some downstream effects. Interwoven pathways yet to be elucidated might involve turning in and off some routes, such as the control of keratinolytic activity and the germination rates (green arrows). A conceivable hypothesis of the regulation of germination rates and cell cycle is associated with a stress response mechanism (yellow dashed arrow).

Hence, we decided to evaluate the kinetics of biofilm formation and biomass production in the Δ*stuA* strain of *T. rubrum.* The involvement of StuA in spore germination and development has been widely discussed. Therefore, to rule out any of these features as interference factors in biofilm experiments, we previously evaluated the growth of wild-type and Δ*stuA* mutants in RPMI medium, and we obtained similar dried weights (Lang et al., 2020). Hence, based on the biofilm kinetics results, we suggested that the Δ*stuA* strain needed more time to reach the maturation stage than the wild-type strain. This result suggests that the mutant strain takes more time to disperse cells, colonize other surfaces, and restart the cycle of biofilm formation. Previous reports have shed light on the cross-interaction between biofilm formation and concealing of cell wall ligands, which ultimately impacts on host recognition (Lin et al., 2015; Kernien et al., 2017). In addition, biofilm formation relies on MAPK cascade signaling (Chen and Thorner, 2007) and on hydrophobin spore coating, as previously shown for RodA and DewC in *A. fumigatus* (Carrion Sde et al., 2013; Brown et al., 2016). In this sense, the causative effect of StuA deletion on biofilm formation and immunomodulation may occur due to deep changes in overall metabolism in the Δ*stuA* strain, which trigger important differences in cell wall pattern construction. We proposed a model that illustrates the current knowledge on the main processes potentially regulated by StuA in *T. rubrum* (**Figure 6**).

In summary, this study demonstrated that StuA plays a crucial role in overall fungal metabolism, cell wall construction, and host recognition. How these paths cross with StuA in this complex regulatory circuit remains to be investigated. Nevertheless, based on the present and previous data, the tight role of StuA in central metabolism is fundamental for cellular physiology and virulence traits.

## Data Availability Statement

The datasets presented in this study can be found in online repositories. The generated RNA-seq dataset is available at the Gene Expression Omnibus (http://www.ncbi.nlm.nih.gov/geo), under the accession numbers GSE163357 and GSE134406.

## Author Contributions

NM-R, TB, JN-d-R, and AR designed the research. TB and JN-d-R performed the experimental design and laboratory experiments. PS performed the bioinformatics analyses. MM and EL assisted in RNA-seq construction. JB assisted in biofilm assays. TB, JN-d-R, NM-R, and AR wrote the manuscript. All authors reviewed the manuscript. All authors contributed to the article and approved the submitted version.

## Funding

This work was supported by grants from the Brazilian Agencies: São Paulo Research Foundation - FAPESP [proc. No. 2019/22596-9, and Fellowships No. 2015/23435-8 to TB, No. 2018/15458-6 to JN-d-R, and No. 2018/11319-1 to MM]; National Council for Scientific and Technological Development - CNPq [Grants No. 305797/2017-4 and 304989/2017-7]; Coordenação de Aperfeiçoamento de Pessoal de Nível Superior (CAPES) - Finance Code 001, and Fundação de Apoio ao Ensino, Pesquisa e Assistência - FAEPA.

## Conflict of Interest

The authors declare that the research was conducted in the absence of any commercial or financial relationships that could be construed as a potential conflict of interest.
