# Supplementary material for: StuA-Regulated Processes in the Dermatophyte Trichophyton rubrum: Transcription Profile, Cell-Cell Adhesion, and Immunomodulation
Source: Front Cell Infect Microbiol. 2021 Jun 8;11:643659. doi: 10.3389/fcimb.2021.643659 (PMC8218993; doi:10.3389/fcimb.2021.643659)
Supplement: Supplementary file 1 [file DataSheet_1.pdf]

Supplementary Material

StuA-regulated processes in the dermatophyte *Trichophyton rubrum*: transcription profile, cell-cell adhesion, and immunomodulation

Supplementary Table S1. Primer sets used for real-time quantitative reverse transcription polymerase chain reaction (qRT-PCR).

Gene ID	Sequence 5'-3'	Concentration (nM)	Efficiency (%)
TERG_02307	F: CTGATGAGGAGTACTTGCCTAC<br>R: CTATCCACTGCTGGTCATCTT	300	91.2
TERG_11637	F: CAAGTCAAGAACTGGTGGAAAG<br>R: CTCTGTGCATTTGATGGGTAATC	100	93.5
TERG_07959	F: CCGACTTTGAGCAGGACTTT<br>R: CTGGGAGAATGAAAGCAGTGT	300	94.4
TERG_06651	F: GCTGTCACTCGGTTATACCT<br>R: CATCTCGGATGCGTCTCTAA	100	92.9
TERG_08114	F: CTTCCTCAACCTTAGCTTCTCC<br>R: ATTCGGGTGGTAGTTCGTATTC	300	96.4
TERG_00060	F: CATGGAATTGCTCACGAAAGG<br>R: GATGCTGTGGTTGCTTGTATG	100	101.7
TERG_00119	F: GTGCGACTCTTCAGACAGATAA<br>R: TGAACCGTCAAGCAGGATATT	100	90.6
TERG_01840	F: ATGATGCTTGCCTACCAACC<br>R: TGACTGTTCCGGTAGCAATG	300	91.2
TERG_01900	F: GCCACGGTTGATGTCCTATT<br>R: CAAAGAACGGAGCGACCATA	100	97.8
TERG_01901	F: GGTTTACCACTGTCCACCTATC<br>R: AACGCAAGTCTTCCCTCATC	100	94.0
TERG_02368	F: GCGTGTCTTGCCTGGTATCT<br>R: TAGGAGTTTCGCGATTTTGG	300	99.9
TERG_03544	F: CTCGCTCTTAGCCAGTTTGT<br>R: GACGGCGTCTGTGAAGTATT	100	92.0
TERG_04282	F: CCCTTTGAAATTCGCCATGAG<br>R: TTACTACTGGCTGCGGAAAG	100	90.1
TERG_08261	F: GCGAGCTTCACTCTTAACTTCT<br>R: GCATTATAGCACGGTATCCTCTC	300	90.5
TERG_01950	F: CGGGTCTATCTTCTGGTACA<br>R: GGTTAACGGGATCTTCAAGG	300	95.3
TERG_05627	F: GGCGTGGAATCCGTACATTA<br>R: GCATGGGCATAGACGTAGATATAG	100	94.6

Supplementary Table S2. General features of the RNA sequencing reads mapped on the *Trichophyton rubrum* reference genome.

Sample	Raw reads	High-quality reads	Mapped reads STAR	Total mapped reads (%)
Δ*stuA* libraries				
Δ*stuA* Glucose 24 hours I	9,171,325	8,898,166	8,493,828	95.46
Δ*stuA* Glucose 24 hours II	10,541,692	10,326,195	9,899,856	95.87
Δ*stuA* Glucose 24 hours III	12,036,641	11,751,222	11,267,440	95.88
Δ*stuA* Glucose 48 hours I	10,116,417	9,891,678	9,664,321	97.70
Δ*stuA* Glucose 48 hours II	10,289,669	10,070,030	9,720,639	96.53
Δ*stuA* Glucose 48 hours III	13,694,576	13,406,285	12,889,091	96.14
Δ*stuA* Glucose 96 hours I	8,795,089	8,539,811	8,265,888	96.79
Δ*stuA* Glucose 96 hours II	11,644,168	11,423,553	10,308,226	90.24
Δ*stuA* Glucose 96 hours III[a]	23,274	22,163	13,189	59.51
Δ*stuA* Keratin 24 hours I	8,073,262	7,889,656	7,482,678	94.84
Δ*stuA* Keratin 24 hours II	8,451,660	8,266,135	7,540,055	91.22
Δ*stuA* Keratin 24 hours III	12,550,744	12,274,004	11,429,534	93.12
Δ*stuA* Keratin 48 hours I	9,035,221	8,826,673	8,384,302	94.99
Δ*stuA* Keratin 48 hours II	9,070,728	8,879,514	8,317,581	93.67
Δ*stuA* Keratin 48 hours III	12,184,619	11,891,866	10,944,759	92.04
Δ*stuA* Keratin 96 hours I	12,566,366	12,309,111	11,877,436	96.49
Δ*stuA* Keratin 96 hours II	10,315,165	10,083,929	9,512,786	94.34
Δ*stuA* Keratin 96 hours III	14,405,588	13,990,682	12,594,826	90.02
WT libraries				
WT Glucose 24 hours I	10,298,058	10,079,869	9,513,704	94.38
WT Glucose 24 hours II	11,730,284	11,470,387	9,274,834	80.86
WT Glucose 24 hours III	13,134,053	12,870,769	12,130,966	94.25
WT Glucose 48 hours I	12,139,571	11,873,826	11,045,360	93.02
WT Glucose 48 hours II	8,484,471	8,220,328	7,582,730	92.24
WT Glucose 48 hours III	13,900,719	13,563,244	12,759,015	94.07
WT Glucose 96 hours I	10,846,570	10,620,463	9,913,587	93.34
WT Glucose 96 hours II	9,950,533	9,707,227	9,214,841	94.93
WT Glucose 96 hours III	12,788,333	12,287,043	11,533,469	93.87
WT Keratin 24 hours I	9,586,259	9,339,491	7,040,104	75.38
WT Keratin 24 hours II	10,093,406	9,815,286	8,266,023	84.22
WT Keratin 24 hours III	13,910,853	13,576,336	11,848,476	87.27
WT Keratin 48 hours I	11,636,035	11,292,471	9,374,940	83.02
WT Keratin 48 hours II	9,081,225	8,769,772	6,548,726	74.67
WT Keratin 48 hours III	10,036,969	9,786,133	8,751,212	89.42
WT Keratin 96 hours I	8,644,359	8,369,738	6,533,731	78.06
WT Keratin 96 hours II	8,440,082	7,993,994	6,216,676	77.77
WT Keratin 96 hours III	12,753,703	12,436,022	9,652,577	77.62

[a] library omitted in the subsequent analyses.

Supplemetary Table S3. Top 10 genes modulated in Δ*stuA* mutant *Trichophyton rubrum* grown in glucose medium.

ID	Gene Product Name	$\log_2$(Fold change)
**<u>24 hours (Glucose)</u>**		
	**Up-regulated**	
TERG_07028	hypothetical protein	6.46
TERG_00598	hypothetical protein	5.25
TERG_04115	aldo-keto reductase, putative (*T. verrucosum*)	4.92
TERG_01917	O-methyltransferase, putative (*A. benhamiae*)	4.70
TERG_00235	hypothetical protein	4.53
TERG_07166	hypothetical protein	4.50
TERG_01004	extracellular sialidase/neuraminidase (*T. equinum*)	4.30
TERG_08358	dimethylallyl tryptophan synthase GliD1 (*T. equinum*)	4.23
TERG_06585	hypothetical protein	4.16
TERG_02820	polyketide synthase (*T. tonsurans*)	4.15
	**Down-regulated**	
TERG_04738	hypothetical protein	-8.64
TERG_06049	dimethylallyl tryptophan synthase, putative (*T. verrucosum*)	-8.17
TERG_02412	HHE domain protein (*T. verrucosum*)	-7.24
TERG_02134	indoleamine 2,3-dioxygenase (*T. equinum*)	-7.13
TERG_02842	6-hydroxy-D-nicotine oxidase (*T. equinum*)	-6.86
TERG_11863	hypothetical protein	-6.61
TERG_06267	hypothetical protein	-6.52
TERG_03161	hypothetical protein	-6.30
TERG_06051	4-dimethylallyltryptophan methyltransferase (*T. tonsurans*)	-5.77
TERG_04028	cytochrome P450, putative (*T. verrucosum*)	-5.62
**<u>48 hours (Glucose)</u>**		
	**Up-regulated**	
TERG_08358	dimethylallyl tryptophan synthase GliD1 (*T. equinum*)	8.43
TERG_07028	hypothetical protein	7.07
TERG_02952	oxidoreductase, short chain dehydrogenase/reductase family (*A. benhamiae*)	6.49
TERG_08229	hypothetical protein	6.12
TERG_04115	aldo-keto reductase, putative (*T. verrucosum*)	5.55
TERG_11686	hypothetical protein	5.35
TERG_08778	hypothetical protein	5.11
TERG_00598	hypothetical protein	4.93
TERG_00161	hypothetical protein	4.56
TERG_06601	flavin-containing amine oxidasedehydrogenase (*T. equinum*)	4.39
	**Down-regulated**	
TERG_06267	hypothetical protein	-8.01
TERG_03161	hypothetical protein	-7.26
TERG_04282	GPI ethanolamine phosphate transferase (*T. tonsurans*)	-7.00
TERG_06049	dimethylallyl tryptophan synthase, putative (*T. verrucosum*)	-6.83
TERG_02134	indoleamine 2,3-dioxygenase (*T. equinum*)	-6.28

TERG_06054	hypothetical protein	-6.14
TERG_08444	CipC protein (*M. canis*)	-6.11
TERG_04028	cytochrome P450, putative (*T. verrucosum*)	-6.11
TERG_06051	4-dimethylallyltryptophan methyltransferase (*T. tonsurans*)	-6.03
TERG_01279	flavin-binding monooxygenase (*T. equinum*)	-5.65
**96 hours (Glucose)**		
	**Up-regulated**	
TERG_07028	hypothetical protein	6.69
TERG_04464	hypothetical protein	5.98
TERG_04551	hypothetical protein	5.13
TERG_12391	hypothetical protein	4.94
TERG_03919	phytoene dehydrogenase (*T. equinum*)	4.59
TERG_06035	hypothetical protein	4.44
TERG_12090	hypothetical protein	3.73
TERG_12042	RNA helicase/RNAse III, putative (*A. benhamiae*)	3.70
TERG_06601	flavin-containing amine oxidasedehydrogenase (*T. equinum*)	3.65
TERG_05965	hypothetical protein	3.56
	**Down-regulated**	
TERG_06267	hypothetical protein	-8.73
TERG_05445	DUF323 domain-containing protein (*T. equinum*)	-7.99
TERG_05733	salivary glue protein Sgs-3 (*M. canis*)	-6.75
TERG_07504	sialidase (*T. tonsurans*)	-6.15
TERG_06049	dimethylallyl tryptophan synthase, putative (*T. verrucosum*)	-5.95
TERG_03161	hypothetical protein	-5.86
TERG_02380	hypothetical protein	-5.78
TERG_04282	GPI ethanolamine phosphate transferase (*T. tonsurans*)	-5.57
TERG_05615	hsp70-like protein (*T. equinum*)	-5.55
TERG_02134	indoleamine 2,3-dioxygenase (*T. equinum*)	-5.49

Supplementary Table S4. Top 10 genes modulated in Δ*stuA* mutant *Trichophyton rubrum* grown on keratin.

ID	Gene Product Name	log_2(Fold change)
**<u>24 hours (Keratin)</u>**		
	**Up-regulated**	
TERG_06585	hypothetical protein	8.28
TERG_07028	hypothetical protein	7.30
TERG_01913	cytochrome P450 monooxygenase, putative (*A. benhamiae*)	6.14
TERG_05733	salivary glue protein Sgs-3 (*M. canis*)	6.08
TERG_01915	O-methyltransferase (*A. benhamiae*)	6.06
TERG_12594	oxidoreductase, short-chain dehydrogenase/reductase family (*T. verrucosum*)	5.01
TERG_12089	cytochrome P450, putative (*T. verrucosum*)	4.72
TERG_01917	O-methyltransferase, putative (*A. benhamiae*)	4.57
TERG_05442	hypothetical protein	4.56
TERG_11528	tenascin C (*T. equinum*)	4.55
	**Down-regulated**	
TERG_06802	hypothetical protein	-5.48
TERG_04738	hypothetical protein	-5.34
TERG_06414	DUF1338 domain-containing protein (*T. equinum*)	-5.02
TERG_06651	cytochrome P450, putative (*T. verrucosum*)	-4.59
TERG_01599	hypothetical protein	-4.42
TERG_00714	cell pattern formation-associated protein (*T. tonsurans*)	-4.19
TERG_02365	hypothetical protein	-4.13
TERG_06160	nitrite reductase, copper-containing	-4.10
TERG_08806	hypothetical protein	-4.08
TERG_06854	glutamine-serine-proline rich protein, putative (*A. benhamiae*)	-4.05
**<u>48 hours (Keratin)</u>**		
	**Up-regulated**	
TERG_07357	hypothetical protein	7.63
TERG_06585	hypothetical protein	6.71
TERG_03345	hypothetical protein	6.11
TERG_04047	cysteine-rich secreted protein (*A. benhamiae*)	5.70
TERG_12594	oxidoreductase, short-chain dehydrogenase/reductase family (*T. verrucosum*)	5.33
TERG_03198	hypothetical protein	5.07
TERG_01913	cytochrome P450 monooxygenase, putative (*A. benhamiae*)	4.99
TERG_05141	NCS1 nucleoside transporter (*T. equinum*)	4.96
TERG_11907	FAD binding domain containing protein (*T. equinum*)	4.95
TERG_01915	O-methyltransferase (*A. benhamiae*)	4.95
	**Down-regulated**	
TERG_06414	DUF1338 domain-containing protein (*T. equinum*)	-7.31
TERG_00235	hypothetical protein	-6.67
TERG_05943	aminotransferase, class III (*T. verrucosum*)	-5.81
TERG_08444	CipC protein (*M. canis*)	-5.59
TERG_00060	GPI anchored cell wall protein, putative (*A. benhamiae*)	-5.16

TERG_08182	hypothetical protein	-5.14
TERG_00026	flavin-binding monooxygenase, putative (*T. verrucosum*)	-5.05
TERG_04738	hypothetical protein	-5.00
TERG_03732	hypothetical protein	-4.89
TERG_06854	glutamine-serine-proline rich protein, putative (*A. benhamiae*)	-4.88

96 hours (Keratin)

	**Up-regulated**	
TERG_01659	heat shock protein Hsp30-like, putative (*A. benhamiae*)	5.83
TERG_12507	hsp98-like protein (*T. equinum*)	5.14
TERG_00499	hypothetical protein	5.11
TERG_06585	hypothetical protein	4.87
TERG_07949	heat shock protein 78 (*T. tonsurans*)	4.69
TERG_07357	hypothetical protein	4.69
TERG_01731	hypothetical protein	4.27
TERG_03390	glutation S-transferase (*T. equinum*)	4.25
TERG_07099	hypothetical protein	4.17
TERG_05141	NCS1 nucleoside transporter (*T. equinum*)	4.16
	**Down-regulated**	
TERG_00235	hypothetical protein	-8.18
TERG_06414	DUF1338 domain-containing protein (*T. equinum*)	-7.38
TERG_05943	aminotransferase, class III (*T. verrucosum*)	-6.54
TERG_07504	sialidase (*T. tonsurans*)	-5.70
TERG_04879	hypothetical protein	-5.68
TERG_03311	hypothetical protein	-5.63
TERG_07326	glutathione S-transferase PARB (*T. equinum*)	-5.62
TERG_08444	CipC protein (*M. canis*)	-5.20
TERG_05611	hypothetical protein	-5.20
TERG_03732	hypothetical protein	-5.09

Supplementary Table S5. Differentially expressed genes in Δ*stuA* vs. wild-type *Trichophyton rubrum* grown in glucose medium or keratin.

Δ*stuA* vs. wild type (Glucose)

ID	24 hours	48 hours	96 hours	Gene Product Name
TERG_01917	4.70			O-methyltransferase, putative (*A. benhamiae*)
TERG_00235	4.53			hypothetical protein
TERG_06585	4.16			hypothetical protein
TERG_07357	3.78			hypothetical protein
TERG_01915	3.35			O-methyltransferase (*A. benhamiae*)
TERG_01914	3.20			trichodiene oxygenase (*T. equinum*)
TERG_01668	3.20			DUF396 doamin protein (*T. equinum*)
TERG_00127	2.93			secretory phospholipase A2 (*T. tonsurans*)
TERG_11907	2.93			FAD binding domain containing protein (*T. equinum*)
TERG_11906	2.91			FAD binding domain containing protein (*T. equinum*)
TERG_01913	2.79			cytochrome P450 monooxygenase, putative (*A. benhamiae*)
TERG_04066	2.73			filamentation protein (Rhf1), putative (*T. verrucosum*)
TERG_08029	2.38			HMG box protein, putative (*T. verrucosum*)
TERG_11805	2.37			hypothetical protein
TERG_08289	2.33			Rpp14 family protein (*T. equinum*)
TERG_08180	2.32			oxalate decarboxylase (*T. equinum*)
TERG_01994	2.23			OPT family oligopeptide transporter
TERG_05968	2.17			hypothetical protein
TERG_05124	2.13			gliotoxin biosynthesis protein GliK (*T. verrucosum*)
TERG_03947	2.12			hypothetical protein
TERG_05453	2.08			amino acid permease (*T. equinum*)
TERG_12217	2.08			hypothetical protein
TERG_07220	2.06			hypothetical protein
TERG_04329	2.03			chitosanase, putative (*T. verrucosum*)
TERG_00736	2.02			alpha/beta hydrolase, putative (*A. benhamiae*)
TERG_06547	2.02			glycerophosphoryl diester phosphodiesterase (*T. tonsurans*)
TERG_12337	2.01			hypothetical protein
TERG_12679	2.00			JmjC domain-containing protein (*T. tonsurans*)
TERG_05524	1.95			3-deoxy-7-phosphoheptulonate synthase
TERG_06761	1.95			CAMK protein kinase (*T. tonsurans*)
TERG_08346	1.95			hypothetical protein
TERG_02513	1.94			TAM domain methyltransferase (*T. equinum*)
TERG_12142	1.92			hypothetical protein
TERG_06548	1.91			hypothetical protein
TERG_04863	1.90			hypothetical protein
TERG_08666	1.89			hypothetical protein
TERG_05850	1.87			integral membrane protein (*A. benhamiae*)
TERG_01713	1.86			C6 finger domain protein, putative (*T. verrucosum*)
TERG_12112	1.86			hypothetical protein

TERG_07998	1.85	hypothetical protein
TERG_02099	1.84	uracil permease (*T. equinum*)
TERG_08378	1.82	hypothetical protein
TERG_04615	1.78	hypothetical protein
TERG_05443	1.78	hypothetical protein
TERG_08400	1.78	hypothetical protein
TERG_11529	1.77	hypothetical protein
TERG_07564	1.77	RNA polymerase II mediator complex subunit Srb5, putative (*T. verrucosum*)
TERG_01638	1.77	hypothetical protein
TERG_11903	1.77	hypothetical protein
TERG_06502	1.76	histone chaperone RTT106 (*T. tonsurans*)
TERG_11710	1.74	hypothetical protein
TERG_03179	1.73	NlpC/P60-like cell-wall peptidase, putative (*A. benhamiae*)
TERG_11806	1.73	hypothetical protein
TERG_01947	1.70	hypothetical protein
TERG_06211	1.68	DUF498 domain-containing protein (*T. equinum*)
TERG_00307	1.67	hypothetical protein
TERG_06968	1.66	MIND kinetochore complex component Nnf1 (*T. equinum*)
TERG_11527	1.66	tenascin C (*T. equinum*)
TERG_12214	1.61	hypothetical protein
TERG_00027	1.60	F-box domain protein (*A. benhamiae*)
TERG_01740	1.60	alpha,alpha-trehalose phosphate synthase subunit, putative (*A. benhamiae*)
TERG_11592	1.59	palmitoyl-protein thioesterase (*T. tonsurans*)
TERG_08351	1.59	hypothetical protein
TERG_06745	1.58	hypothetical protein
TERG_04855	1.57	hypothetical protein
TERG_08377	1.56	copper transporter Ctr (*T. tonsurans*)
TERG_08662	1.55	hypothetical protein
TERG_02974	1.55	hypothetical protein
TERG_02378	1.54	hypothetical protein
TERG_01760	1.53	protein-ER retention protein (*T. tonsurans*)
TERG_01594	1.52	hypothetical protein
TERG_02423	1.52	hypothetical protein
TERG_04763	-1.51	fructosyl amine:oxygen oxidoreductase (*T. tonsurans*)
TERG_03340	-1.52	oxidoreductase (*T. equinum*)
TERG_01494	-1.53	hypothetical protein
TERG_11594	-1.53	serine carboxypeptidase (*T. tonsurans*)
TERG_06147	-1.54	pyridine nucleotide-disulfide oxidoreductase, putative (*A. benhamiae*)
TERG_07211	-1.55	NAD binding Rossmann fold oxidoreductase, putative (*A. benhamiae*)
TERG_05418	-1.57	hypothetical protein
TERG_11728	-1.57	C6 transcription factor, putative (*T. verrucosum*)

TERG_05044	-1.57	epoxide hydrolase, putative (*A. benhamiae*)
TERG_06888	-1.57	transulfuration enzyme family protein, putative (*A. benhamiae*)
TERG_05342	-1.58	MFS multidrug transporter, putative (*A. benhamiae*)
TERG_01215	-1.58	chromosome segregation protein (*T. tonsurans*)
TERG_07083	-1.59	hypothetical protein
TERG_05585	-1.59	short-chain dehydrogenase/reductase SDR (*T. equinum*)
TERG_04082	-1.59	DUF89 domain-containing protein (*T. equinum*)
TERG_01197	-1.59	nitrilase (*T. tonsurans*)
TERG_12576	-1.59	jumonji family transcription factor (*T. equinum*)
TERG_00976	-1.61	serine hydroxymethyltransferase, cytosolic
TERG_03672	-1.62	hypothetical protein
TERG_02953	-1.62	hypothetical protein
TERG_01338	-1.62	hydantoinase (*T. equinum*)
TERG_07235	-1.64	fructose-1,6-bisphosphatase (*T. tonsurans*)
TERG_12721	-1.64	transferase (*M. canis*)
TERG_03758	-1.65	hypothetical protein
TERG_06390	-1.68	sulfite reductase flavoprotein alpha-component (*T. equinum*)
TERG_00067	-1.69	hypothetical protein
TERG_08617	-1.71	hypothetical protein
TERG_06810	-1.71	hypothetical protein
TERG_06233	-1.72	hypothetical protein
TERG_01955	-1.73	YjeF C (*T. tonsurans*)
TERG_00820	-1.77	MFS multidrug transporter, putative (*A. benhamiae*)
TERG_00974	-1.77	2,3-bisphosphoglycerate-independent phosphoglycerate mutase
TERG_03671	-1.78	hypothetical protein
TERG_07673	-1.79	hypothetical protein
TERG_03698	-1.79	hypothetical protein
TERG_12281	-1.80	glycosyl hydrolase (*T. tonsurans*)
TERG_08058	-1.80	alpha-1,2-mannosidase family protein (*T. verrucosum*)
TERG_06234	-1.82	2,5-diketo-D-gluconic acid reductase A (*T. equinum*)
TERG_05876	-1.87	hypothetical protein
TERG_03780	-1.88	pyruvate dehydrogenase dihydrolipoamide acetyltransferase component (*M. gypseum*)
TERG_08443	-1.94	adenosylhomocysteinase
TERG_02474	-1.95	hypothetical protein
TERG_06376	-1.96	hypothetical protein
TERG_00324	-2.00	stress response protein ish1 (*M. canis*)
TERG_11963	-2.02	hypothetical protein
TERG_08299	-2.04	multidrug resistance-associated protein 1 transporter (*M. canis*)
TERG_00942	-2.09	hypothetical protein
TERG_11637	-2.14	isocitrate lyase
TERG_07035	-2.16	hypothetical protein
TERG_04521	-2.18	HypA-like protein, putative (*A. benhamiae*)
TERG_01844	-2.23	hypothetical protein
TERG_02009	-2.25	short chain oxidoreductase (CsgA), putative (*T. verrucosum*)

TERG_05843	-2.30			F-box domain-containing protein (*T. equinum*)
TERG_02555	-2.32			DUF636 domain containing protein (*T. equinum*)
TERG_02671	-2.33			hypothetical protein
TERG_06235	-2.41			hypothetical protein
TERG_12096	-2.48			OmtA protein (*M. canis*)
TERG_07280	-2.49			hypothetical protein
TERG_04427	-2.51			serine/threonine protein kinase
TERG_01641	-2.55			FK506 suppressor Sfk1 (*T. equinum*)
TERG_08965	-2.58			ubiquitin-conjugating enzyme E2S (*T. equinum*)
TERG_05816	-2.60			hypothetical protein
TERG_03105	-2.61			hypothetical protein
TERG_00370	-2.63			glutathione S-transferase (*T. tonsurans*)
TERG_00414	-2.66			3-beta hydroxysteroid dehydrogenase/isomerase family protein (*A. benhamiae*)
TERG_04714	-2.70			mitochondrial phosphate carrier protein (*T. tonsurans*)
TERG_00500	-2.75			hypothetical protein
TERG_11650	-3.02			hypothetical protein
TERG_01499	-3.50			NmrA family protein (*T. equinum*)
TERG_08344	-3.83			hypothetical protein
TERG_02841	-4.22			AURR1 aurofusarin regulatory protein (*T. equinum*)
TERG_05428	-5.19			hypothetical protein
TERG_11863	-6.61			hypothetical protein

Gene expression values are expressed in log_2 fold change.

ID	24 hours	48 hours	96 hours	Gene Product Name
TERG_02952		6.49		oxidoreductase, short chain dehydrogenase/reductase family (*A. benhamiae*)
TERG_08229		6.12		hypothetical protein
TERG_11686		5.35		hypothetical protein
TERG_08778		5.11		hypothetical protein
TERG_07011		4.34		conserved fungal protein (*A. benhamiae*)
TERG_05141		4.31		NCS1 nucleoside transporter (*T. equinum*)
TERG_12195		3.49		developmental regulator flbA (*M. gypseum*)
TERG_06785		3.42		cytochrome P450 monooxygenase (*T. equinum*)
TERG_04118		3.42		hypothetical protein
TERG_08262		3.41		C6 transcription factor, putative (*A. benhamiae*)
TERG_08643		3.40		hypothetical protein
TERG_12068		3.38		PLC-E (*T. equinum*)
TERG_08247		3.33		hypothetical protein
TERG_02955		3.22		CP2 transcription factor, putative (*A. benhamiae*)
TERG_12284		3.21		alkaline serine protease (*T. equinum*)
TERG_01802		3.20		hypothetical protein
TERG_11764		3.12		proline-threonine-rich repeat protein (*T. tonsurans*)

TERG_08227	3.09	aldo-keto reductase (YakC), putative (*A. benhamiae*)
TERG_04068	2.96	cytochrome P450 monooxygenase, putative (*A. benhamiae*)
TERG_12599	2.94	hypothetical protein
TERG_03955	2.90	RNA 3'-terminal phosphate cyclase, putative (*T. verrucosum*)
TERG_08116	2.89	hypothetical protein
TERG_03707	2.87	geranylgeranyl diphosphate synthase, putative (*A. benhamiae*)
TERG_01148	2.85	hypothetical protein
TERG_01735	2.77	SAP domain-containing protein (*T. equinum*)
TERG_06065	2.76	conserved glycine-rich protein (*T. verrucosum*)
TERG_03172	2.69	sodium/phosphate symporter (*T. tonsurans*)
TERG_01366	2.65	hypothetical protein
TERG_05363	2.62	hypothetical protein
TERG_00213	2.60	hypothetical protein
TERG_06022	2.58	hypothetical protein
TERG_01821	2.57	cysteine synthase (*T. tonsurans*)
TERG_05497	2.57	C6 zinc finger domain-containing protein (*T. equinum*)
TERG_08230	2.55	hypothetical protein
TERG_12370	2.55	MFS multidrug transporter, putative (*A. benhamiae*)
TERG_02445	2.46	hypothetical protein
TERG_00754	2.43	hypothetical protein
TERG_12438	2.41	gamma-glutamyltranspeptidase (*T. tonsurans*)
TERG_03208	2.41	hypothetical protein
TERG_01357	2.39	hypothetical protein
TERG_03130	2.38	C2H2 transcription factor (Rpn4), putative (*T. verrucosum*)
TERG_01382	2.38	hypothetical protein
TERG_11776	2.37	hypothetical protein
TERG_00528	2.34	hypothetical protein
TERG_04951	2.34	U-box domain-containing protein (*T. equinum*)
TERG_12369	2.33	MFS multidrug transporter (*T. tonsurans*)
TERG_12114	2.32	hypothetical protein
TERG_12521	2.31	hypothetical protein
TERG_08602	2.29	alpha-ketoglutarate-dependent taurine dioxygenase (*T. verrucosum*)
TERG_00513	2.29	hypothetical protein
TERG_12600	2.27	U3 small nucleolar ribonucleoprotein IMP4 (*T. tonsurans*)
TERG_11874	2.27	hypothetical protein
TERG_03732	2.27	hypothetical protein
TERG_07239	2.25	zinc-binding oxidoreductase, putative (*A. benhamiae*)
TERG_03113	2.24	Zn-dependent hydrolase/oxidoreductase family protein, putative (*A. benhamiae*)
TERG_12296	2.24	hypothetical protein
TERG_02572	2.23	hypothetical protein
TERG_04200	2.22	hypothetical protein
TERG_11775	2.19	hypothetical protein
TERG_03849	2.19	HET domain protein (*T. tonsurans*)

TERG_00912	2.19	hypothetical protein
TERG_04766	2.18	calcium/proton exchanger
TERG_12663	2.18	hypothetical protein
TERG_11565	2.15	hypothetical protein
TERG_01820	2.14	MFS transporter, putative (*T. verrucosum*)
TERG_12637	2.12	hypothetical protein
TERG_04481	2.09	RTA1 domain protein, putative (*A. benhamiae*)
TERG_05198	2.09	hypothetical protein
TERG_00535	2.09	calcium-translocating P-type ATPase, PMCA-type
TERG_11975	2.07	integral membrane protein (*T. verrucosum*)
TERG_06515	2.07	tetracycline transporter (*T. equinum*)
TERG_11817	2.04	transcription initiation factor TFIID subunit 7, putative (*T. verrucosum*)
TERG_01623	2.03	MFS transporter (*T. equinum*)
TERG_07636	2.03	dual specificity phosphatase (*T. tonsurans*)
TERG_03415	2.01	serine/threonine protein kinase
TERG_05199	2.01	MFS gliotoxin efflux transporter GliA (*T. verrucosum*)
TERG_12140	2.00	lipase (*T. equinum*)
TERG_08044	2.00	GPI anchored protein, putative (*A. benhamiae*)
TERG_11695	1.99	hypothetical protein
TERG_08821	1.99	ribosome biogenesis (*T. equinum*)
TERG_06047	1.99	hypothetical protein
TERG_08815	1.97	hypothetical protein
TERG_03228	1.95	transcriptional regulator PacG/VIB-1 (*T. verrucosum*)
TERG_05993	1.95	DUF124 domain-containing protein (*T. equinum*)
TERG_03240	1.95	transmembrane efflux protein (*T. tonsurans*)
TERG_02510	1.95	hypothetical protein
TERG_03297	1.94	hypothetical protein
TERG_02425	1.92	RNA binding protein (*A. benhamiae*)
TERG_01645	1.90	mitochondrial carrier protein (*T. tonsurans*)
TERG_02972	1.89	SRF-type transcription factor rlmA (*T. equinum*)
TERG_02562	1.89	chitin synthase C
TERG_00611	1.87	para-aminobenzoate synthase PabaA (*T. verrucosum*)
TERG_02081	1.87	RNA binding protein
TERG_02124	1.87	rho-GTPase-activating protein (*T. tonsurans*)
TERG_08982	1.86	hypothetical protein
TERG_04473	1.86	hypothetical protein
TERG_08619	1.86	siderophore iron transporter mirB (*T. equinum*)
TERG_00196	1.86	hypothetical protein
TERG_03892	1.85	CAMK/CAMKL protein kinase (*T. tonsurans*)
TERG_11824	1.85	hypothetical protein
TERG_07408	1.85	acetyltransferase, GNAT family (*A. benhamiae*)
TERG_07143	1.85	potassium/sodium efflux P-type ATPase, fungal-type
TERG_07551	1.84	hypothetical protein

TERG_04472	1.83	hypothetical protein
TERG_01173	1.82	hypothetical protein
TERG_03343	1.82	fatty acid desaturase (*T. equinum*)
TERG_02591	1.82	D-tyrosyl-tRNA(Tyr) deacylase
TERG_05698	1.82	transesterase (LovD), putative (*T. verrucosum*)
TERG_08357	1.82	polyketide synthase, putative (*A. benhamiae*)
TERG_03793	1.82	elongation factor 3 (*M. gypseum*)
TERG_06792	1.82	hypothetical protein
TERG_02993	1.81	allergenic cerato-platanin Asp F13 (*T. verrucosum*)
TERG_02973	1.81	morphogenesis protein (Msb1), putative (*T. verrucosum*)
TERG_11549	1.81	hypothetical protein
TERG_07072	1.81	ATP-dependent RNA helicase DRS1
TERG_08225	1.81	hypothetical protein
TERG_07817	1.80	endoglucanase (*T. equinum*)
TERG_12350	1.80	hypothetical protein
TERG_05429	1.80	MFS multidrug transporter, putative (*T. verrucosum*)
TERG_12373	1.79	multidrug resistance protein (*T. tonsurans*)
TERG_02041	1.79	glutathione S-transferase (*T. equinum*)
TERG_01150	1.79	hypothetical protein
TERG_02570	1.77	potentail helicase MOV-10 (*T. equinum*)
TERG_06929	1.77	chitinase (*T. equinum*)
TERG_06328	1.77	AdoMet-dependent rRNA methyltransferase spb1
TERG_11976	1.76	integral membrane protein (*T. tonsurans*)
TERG_06118	1.76	hypothetical protein
TERG_01568	1.75	ATP-dependent RNA helicase DED1
TERG_08717	1.75	tRNA methyltransferase (*T. tonsurans*)
TERG_05752	1.74	serine/threonine-protein phosphatase PP1
TERG_01636	1.74	ADP-ribosylglycohydrolase (*M. gypseum*)
TERG_12694	1.74	hypothetical protein
TERG_05988	1.74	solid-state culture expressed protein (Aos23), putative (*T. verrucosum*)
TERG_02797	1.73	WD repeat protein (*T. tonsurans*)
TERG_12351	1.73	hypothetical protein
TERG_12573	1.73	CHD5 domain-containing protein (*T. equinum*)
TERG_04184	1.73	CorA family metal ion transporter, putative (*A. benhamiae*)
TERG_08200	1.73	hypothetical protein
TERG_00514	1.72	hypothetical protein
TERG_00273	1.72	mucin (*M. canis*)
TERG_12125	1.72	hypothetical protein
TERG_01863	1.72	hypothetical protein
TERG_03692	1.72	solid-state culture specific protein, putative (*A. benhamiae*)
TERG_01659	1.71	heat shock protein Hsp30-like, putative (*A. benhamiae*)
TERG_02123	1.70	adenylate-forming enzyme, putative (*A. benhamiae*)
TERG_03278	1.69	hypothetical protein
TERG_05551	1.68	hypothetical protein

TERG_03556	1.68	transcriptional regulator Medusa (*A. benhamiae*)
TERG_03512	1.68	hypothetical protein
TERG_04153	1.68	60S ribosome biogenesis protein Brx1 (*T. tonsurans*)
TERG_07317	1.68	phosphatidylinositol phospholipase C (*T. tonsurans*)
TERG_12198	1.66	hypothetical protein
TERG_04509	1.66	hypothetical protein
TERG_06163	1.66	MYB family conidiophore development protein FlbD (*T. equinum*)
TERG_01734	1.65	hypothetical protein
TERG_06827	1.64	hypothetical protein
TERG_01175	1.64	LovB-like polyketide synthase, putative (*A. benhamiae*)
TERG_07570	1.64	G-protein signaling regulator, putative (*T. verrucosum*)
TERG_12502	1.63	C6 finger domain-containing protein (*T. equinum*)
TERG_05226	1.62	nuclear localization protein, putative (*T. verrucosum*)
TERG_11641	1.62	forkhead transcription factor (*T. tonsurans*)
TERG_08924	1.62	MIZ zinc finger domain protein (*A. benhamiae*)
TERG_07957	1.62	hypothetical protein
TERG_07346	1.61	tRNA ligase (*M. gypseum*)
TERG_05367	1.61	nucleolar complex-associated protein 3 (*T. equinum*)
TERG_12609	1.61	hypothetical protein
TERG_00909	1.61	hypothetical protein
TERG_00139	1.61	MADS box transcription factor (*T. tonsurans*)
TERG_01907	1.61	hypothetical protein
TERG_08216	1.60	ATP-dependent RNA helicase dbp6 (*T. equinum*)
TERG_01930	1.60	hypothetical protein
TERG_12374	1.59	UDP-N-acetylglucosaminyltransferase (*T. equinum*)
TERG_03134	1.59	hypothetical protein
TERG_02400	1.59	amino acid transporter, putative (*A. benhamiae*)
TERG_01954	1.59	hypothetical protein
TERG_02549	1.59	hypothetical protein
TERG_05520	1.59	hypothetical protein
TERG_01129	1.59	cell wall glucanase (Scw4), putative (*A. benhamiae*)
TERG_12307	1.58	autophagy protein (*T. tonsurans*)
TERG_01101	1.58	hypothetical protein
TERG_02101	1.58	amino-acid transporter Arg-13 (*T. equinum*)
TERG_04135	1.57	DNA-directed RNA polymerase III subunit RPC2 (*T. equinum*)
TERG_06164	1.57	MYB family conidiophore development protein FlbD (*T. equinum*)
TERG_05936	1.57	AMID-like mitochondrial oxidoreductase, putative (*A. benhamiae*)
TERG_03429	1.57	DNA directed RNA polymerase II 15 kDa subunit (*T. tonsurans*)
TERG_02643	1.57	60S ribosomal protein L20 (*T. tonsurans*)
TERG_08497	1.57	mRNA turnover protein 4 (*T. equinum*)
TERG_07978	1.56	hypothetical protein

TERG_03522	1.56	purine-cytosine permease, putative (*A. benhamiae*)
TERG_08540	1.55	rRNA-processing protein FCF1 (*T. equinum*)
TERG_12672	1.55	hypothetical protein
TERG_03312	1.55	RTA1 domain protein, putative (*A. benhamiae*)
TERG_03843	1.55	chitin synthase B
TERG_08276	1.55	tRNA splicing endonuclease subunit (Sen54), putative (*A. benhamiae*)
TERG_00162	1.55	MFS multidrug transporter, putative (*A. benhamiae*)
TERG_12065	1.55	hypothetical protein
TERG_03704	1.54	integral membrane protein Pth11-like, putative (*T. verrucosum*)
TERG_05085	1.54	Ndc80 complex component Nuf2 (*T. tonsurans*)
TERG_08096	1.52	RNA splicing factor (*T. tonsurans*)
TERG_01741	1.52	hypothetical protein
TERG_07999	1.52	hypothetical protein
TERG_02508	1.51	ABC multidrug transporter, putative (*A. benhamiae*)
TERG_08686	1.51	hypothetical protein
TERG_04024	1.51	carbamoyl-phosphate synthase arginine-specific small chain
TERG_03602	1.51	Polysaccharide deacetylase (*T. tonsurans*)
TERG_02278	1.51	hypothetical protein
TERG_03790	1.50	DUF1275 domain-containing protein (*T. equinum*)
TERG_06611	1.50	hypothetical protein
TERG_08240	1.50	methyltransferase, putative (*T. verrucosum*)
TERG_07784	-1.50	hypothetical protein
TERG_01177	-1.50	cytochrome P450 51 (*T. equinum*)
TERG_00689	-1.50	AUR protein kinase
TERG_04412	-1.50	DNA replication factor C subunit Rfc3 (*T. equinum*)
TERG_07804	-1.51	PBSP domain-containing protein (*T. tonsurans*)
TERG_01624	-1.52	phosphomannomutase (*T. tonsurans*)
TERG_12626	-1.53	C-5 sterol desaturase (*T. equinum*)
TERG_12305	-1.53	C6 transcription factor, putative (*T. verrucosum*)
TERG_02912	-1.53	MFS transporter, putative (*A. benhamiae*)
TERG_02481	-1.53	F-box domain-containing protein (*M. canis*)
TERG_01091	-1.53	magnesium-dependent phosphatase-1
TERG_05903	-1.54	hypothetical protein
TERG_05809	-1.55	hypothetical protein
TERG_05136	-1.55	cytochrome P450 oxidoreductase GliC (*A. benhamiae*)
TERG_12517	-1.55	phosphotransferase enzyme family protein (*T. equinum*)
TERG_12253	-1.56	RNA polymerase II Mediator complex subunit Sin4 (*T. equinum*)
TERG_02363	-1.56	succinate-semialdehyde dehydrogenase (*T. tonsurans*)
TERG_06598	-1.56	hypothetical protein
TERG_12490	-1.56	hypothetical protein
TERG_01895	-1.58	pheromone processing carboxypeptidase (Sxa2), putative (*T. verrucosum*)
TERG_07691	-1.58	sterol carrier protein (*T. tonsurans*)
TERG_07798	-1.59	hypothetical protein

TERG_02007	-1.59	enoyl-CoA hydratase/isomerase family protein (*T. verrucosum*)
TERG_00441	-1.59	peptidyl-prolyl cis-trans isomerase pin4
TERG_07963	-1.59	hypothetical protein
TERG_05879	-1.60	GPI mannosyltransferase (*T. tonsurans*)
TERG_12291	-1.60	mitochondrial cytochrome b2 (*T. tonsurans*)
TERG_01295	-1.60	oxidoreductase, short-chain dehydrogenase/reductase family (*T. verrucosum*)
TERG_02921	-1.60	hypothetical protein
TERG_01957	-1.60	proteinase T (*M. gypseum*)
TERG_06415	-1.60	hypothetical protein
TERG_07230	-1.60	MFS monosaccharide transporter, putative (*A. benhamiae*)
TERG_07485	-1.60	UDP-galactopyranose mutase (*T. equinum*)
TERG_02004	-1.61	ureidoglycolate hydrolase, putative (*T. verrucosum*)
TERG_11774	-1.61	hypothetical protein
TERG_02249	-1.62	phenylacetyl-CoA ligase (*T. tonsurans*)
TERG_06760	-1.62	ATP binding L-PSP endoribonuclease family protein, putative (*T. verrucosum*)
TERG_00487	-1.63	hypothetical protein
TERG_00824	-1.64	neutral trehalase
TERG_12087	-1.64	hypothetical protein
TERG_04247	-1.65	CMGC/SRPK protein kinase
TERG_02699	-1.65	protein kinase, putative (*T. verrucosum*)
TERG_00199	-1.66	serine/threonine protein kinase
TERG_02988	-1.66	asparaginase (*T. tonsurans*)
TERG_07037	-1.66	hypothetical protein
TERG_03985	-1.66	hypothetical protein
TERG_04769	-1.67	extracellular serine carboxypeptidase, putative (*T. verrucosum*)
TERG_05153	-1.68	MFS transporter, putative (*A. benhamiae*)
TERG_12075	-1.68	hypothetical protein
TERG_00881	-1.69	zinc-containing alcohol dehydrogenase, putative (*T. verrucosum*)
TERG_04857	-1.69	ribose 5-phosphate isomerase
TERG_11816	-1.69	OPT peptide transporter Mtd1, putative (*T. verrucosum*)
TERG_00550	-1.69	hypothetical protein
TERG_11522	-1.69	hypothetical protein
TERG_03102	-1.69	sterol 24-C-methyltransferase (*T. equinum*)
TERG_01259	-1.70	hypothetical protein
TERG_03619	-1.70	transcription factor Tos4 (*T. equinum*)
TERG_06712	-1.71	mitochondrial methylglutaconyl-CoA hydratase (*T. tonsurans*)
TERG_12129	-1.72	hypothetical protein
TERG_01732	-1.72	amidohydrolase (*T. equinum*)
TERG_11807	-1.72	protein kinase subdomain-containing protein (*T. tonsurans*)
TERG_01754	-1.73	oxidoreductase, 2-nitropropane dioxygenase family, putative (*A. benhamiae*)
TERG_12725	-1.74	MFS transporter (*T. equinum*)

TERG_03464	-1.74	stress response protein (*T. equinum*)
TERG_03747	-1.74	phospholipase A2 (*T. equinum*)
TERG_05841	-1.76	AmmeMemoRadiSam system protein B
TERG_05579	-1.77	FAD dependent oxidoreductase, putative (*A. benhamiae*)
TERG_07045	-1.78	hypothetical protein
TERG_06980	-1.78	hypothetical protein
TERG_11814	-1.78	FAD dependent oxidoreductase, putative (*A. benhamiae*)
TERG_06276	-1.79	chromate ion transporter (*T. tonsurans*)
TERG_03318	-1.80	N(5)-glutamine methyltransferase MTQ2 (*T. equinum*)
TERG_06663	-1.80	dienelactone hydrolase (*T. equinum*)
TERG_11808	-1.81	protein kinase subdomain-containing protein (*T. equinum*)
TERG_07597	-1.82	SOK1 (*T. equinum*)
TERG_07412	-1.82	cell division control protein 45
TERG_02381	-1.83	vacuolar protein sorting protein 62 (*M. gypseum*)
TERG_03700	-1.84	hypothetical protein
TERG_07721	-1.85	isopenicillin N-CoA epimerase (*T. equinum*)
TERG_04669	-1.85	hypothetical protein
TERG_04684	-1.86	shugoshin family protein (*A. benhamiae*)
TERG_03808	-1.87	hypothetical protein
TERG_02005	-1.88	catalase (*T. tonsurans*)
TERG_00202	-1.89	hypothetical protein
TERG_00620	-1.90	NAD+ kinase Utr1 (*T. equinum*)
TERG_06879	-1.91	acetamidase (*T. verrucosum*)
TERG_04288	-1.92	hypothetical protein
TERG_00168	-1.93	hypothetical protein
TERG_05137	-1.94	microsomal dipeptidase (*T. tonsurans*)
TERG_08070	-1.95	enoyl-CoA hydratase/isomerase (*T. tonsurans*)
TERG_01384	-1.96	C6 finger domain-containing protein (*T. equinum*)
TERG_01164	-1.97	beta-alanine synthase, putative (*T. verrucosum*)
TERG_07510	-1.98	hypothetical protein
TERG_03541	-2.00	hypothetical protein
TERG_05878	-2.00	translin family protein (*T. tonsurans*)
TERG_11601	-2.00	hypothetical protein
TERG_12511	-2.00	MFS multidrug transporter (*T. equinum*)
TERG_01606	-2.02	peptidase, putative (*A. benhamiae*)
TERG_01950	-2.02	serine/threonine protein kinase (*T. tonsurans*)
TERG_06638	-2.05	endochitinase (*T. equinum*)
TERG_05138	-2.06	PE-PGRS family protein (*T. equinum*)
TERG_01367	-2.07	hypothetical protein
TERG_12518	-2.09	phosphotransferase enzyme family protein (*T. equinum*)
TERG_07300	-2.09	hypothetical protein
TERG_00609	-2.09	ankyrin repeat protein (*T. equinum*)
TERG_06160	-2.10	nitrite reductase, copper-containing
TERG_06285	-2.15	hypothetical protein

TERG_02264	-2.15	hypothetical protein
TERG_02588	-2.19	hypothetical protein
TERG_02078	-2.24	thiamine biosynthesis protein Nmt1 (*T. equinum*)
TERG_05424	-2.26	hypothetical protein
TERG_11813	-2.28	FAD dependent oxidoreductase, putative (*A. benhamiae*)
TERG_08861	-2.33	hypothetical protein
TERG_03027	-2.38	hypothetical protein
TERG_04213	-2.39	TAM domain methyltransferase (*T. equinum*)
TERG_02746	-2.40	hypothetical protein
TERG_07960	-2.41	hypothetical protein
TERG_00466	-2.42	SirB protein (*T. equinum*)
TERG_00429	-2.45	pisatin demethylase (*M. gypseum*)
TERG_04791	-2.47	hypothetical protein
TERG_06522	-2.49	FMN dependent dehydrogenase, putative (*T. verrucosum*)
TERG_06053	-2.51	catalase (*T. tonsurans*)
TERG_02589	-2.61	formamidase (*T. equinum*)
TERG_08158	-2.78	hypothetical protein
TERG_11852	-2.86	glyoxalase family protein (*T. verrucosum*)
TERG_06883	-2.87	NAD dependent epimerase/dehydratase family protein (*T. verrucosum*)
TERG_05799	-2.87	chlorophyll synthesis pathway protein BchC
TERG_07769	-2.93	hypothetical protein
TERG_08283	-3.00	acetamidase (*T. tonsurans*)
TERG_07231	-3.00	hemolysin-III family protein (*T. equinum*)
TERG_11822	-3.06	peroxisome biosynthesis protein (*T. tonsurans*)
TERG_01480	-3.14	MFS multidrug transporter, putative (*A. benhamiae*)
TERG_04764	-3.62	MFS transporter (*T. tonsurans*)
TERG_05409	-3.64	FAD dependent oxidoreductase (*T. equinum*)

Gene expression values are expressed in log_2 fold change.

ID	24 hours	48 hours	96 hours	Gene Product Name
TERG_04464			5.98	hypothetical protein
TERG_04551			5.13	hypothetical protein
TERG_03919			4.59	phytoene dehydrogenase (*T. equinum*)
TERG_12042			3.70	RNA helicase/RNAse III, putative (*A. benhamiae*)
TERG_04254			3.55	oxidoreductase (*T. equinum*)
TERG_00016			3.45	methionine-R-sulfoxide reductase
TERG_01291			3.30	Rad21/Rec8 N terminal domain protein (*A. benhamiae*)
TERG_08585			3.25	ankyrin repeat protein (*A. benhamiae*)
TERG_12604			2.94	hypothetical protein
TERG_04960			2.80	glutathione S-transferase Ure2-like, putative (*A. benhamiae*)
TERG_01153			2.76	hypothetical protein

TERG_04724	2.67	hypothetical protein
TERG_11792	2.62	hypothetical protein
TERG_08163	2.61	hypothetical protein
TERG_08576	2.59	carboxylesterase (*T. tonsurans*)
TERG_04907	2.57	glyoxalase (*T. tonsurans*)
TERG_00386	2.56	hypothetical protein
TERG_01155	2.53	hypothetical protein
TERG_12078	2.47	hypothetical protein
TERG_03599	2.47	metalloproteinase (*T. equinum*)
TERG_04784	2.46	hypothetical protein
TERG_03722	2.45	hypothetical protein
TERG_00490	2.40	erythromycin esterase (*T. tonsurans*)
TERG_01445	2.31	hypothetical protein
TERG_12469	2.26	hypothetical protein
TERG_05650	2.23	blasticidin-resistance protein (*T. tonsurans*)
TERG_02024	2.22	hypothetical protein
TERG_07031	2.19	hypothetical protein
TERG_08076	2.17	integral membrane protein (*T. equinum*)
TERG_07040	2.16	MFS drug efflux transporter (*T. equinum*)
TERG_12653	2.11	hypothetical protein
TERG_06128	2.10	phosphoribosylglycinamide formyltransferase
TERG_06872	2.05	hypothetical protein
TERG_02012	2.01	adenosine deaminase family protein (*A. benhamiae*)
TERG_07818	2.00	hypothetical protein
TERG_12070	2.00	hypothetical protein
TERG_01319	2.00	hypothetical protein
TERG_01920	1.99	hypothetical protein
TERG_12487	1.95	nonribosomal brevianamide peptide synthase FtmA (*T. verrucosum*)
TERG_03877	1.94	cytochrome P450 monooxygenase, putative (*T. verrucosum*)
TERG_00910	1.90	phosphotransferase enzyme family protein (*T. equinum*)
TERG_04126	1.88	dual specificity phosphatase (*T. equinum*)
TERG_04750	1.87	hypothetical protein
TERG_08211	1.85	acetyltransferase, GNAT family, putative (*A. benhamiae*)
TERG_12069	1.85	hypothetical protein
TERG_00031	1.83	hypothetical protein
TERG_04931	1.82	allantoate permease (*T. equinum*)
TERG_06540	1.82	glutathione transferase (*T. tonsurans*)
TERG_05266	1.82	integral membrane protein (*T. equinum*)
TERG_03004	1.81	3-methyl-2-oxobutanoate hydroxymethyltransferase
TERG_01619	1.80	toxin biosynthesis protein (Tri7), putative (*T. verrucosum*)
TERG_11974	1.80	hypothetical protein
TERG_05938	1.80	tetratricopeptide repeat domain-containing protein (*T. equinum*)
TERG_07170	1.78	MFS transporter, putative (*A. benhamiae*)
TERG_06838	1.76	oxalate/formate antiporter, putative (*T. verrucosum*)

TERG_02100	1.75	cytidine deaminase
TERG_08756	1.73	cercosporin toxin biosynthesis protein (*T. equinum*)
TERG_08591	1.73	multidrug resistance protein (*T. tonsurans*)
TERG_12652	1.72	hypothetical protein
TERG_02306	1.71	integral membrane protein (*T. equinum*)
TERG_06048	1.71	tryptophan dimethylallyltransferase (*T. tonsurans*)
TERG_04224	1.70	ABC transporter
TERG_07786	1.70	NAP family protein (*T. equinum*)
TERG_00316	1.69	hypothetical protein
TERG_12010	1.67	alpha/beta hydrolase (*T. equinum*)
TERG_08951	1.65	hypothetical protein
TERG_05652	1.64	leucine aminopeptidase 1
TERG_05814	1.64	hypothetical protein
TERG_04060	1.64	C2H2 type zinc finger domain-containing protein (*T. equinum*)
TERG_06622	1.63	hydrolase (*T. tonsurans*)
TERG_03857	1.61	phosphotransferase enzyme family protein (*T. equinum*)
TERG_00257	1.61	conserved predicted protein (*A. benhamiae*)
TERG_07758	1.61	hypothetical protein
TERG_12334	1.59	hypothetical protein
TERG_07386	1.57	vacuolar membrane PQ loop repeat protein (*T. tonsurans*)
TERG_07627	1.55	hypothetical protein
TERG_05561	1.55	histone acetyltransferase GCN5 (*T. tonsurans*)
TERG_02031	1.54	methylenetetrahydrofolate dehydrogenase (*T. equinum*)
TERG_06997	-1.51	C6 transcription factor (*T. tonsurans*)
TERG_08101	-1.51	TAM domain methyltransferase (*T. equinum*)
TERG_01026	-1.51	kelch-domain-containing protein (*T. tonsurans*)
TERG_07547	-1.51	RGS domain-containing protein (*T. tonsurans*)
TERG_05744	-1.52	GTP-binding protein EsdC (*T. equinum*)
TERG_08141	-1.52	bZIP transcription factor (LziP), putative (*A. benhamiae*)
TERG_01126	-1.53	1,3-beta-glucanosyltransferase, putative (*T. verrucosum*)
TERG_03032	-1.54	autophagy protein Atg13, putative (*A. benhamiae*)
TERG_07197	-1.55	disintegrin and metalloproteinase domain-containing protein B
TERG_02311	-1.56	hypothetical protein
TERG_00786	-1.56	hypothetical protein
TERG_03322	-1.56	hypothetical protein
TERG_00146	-1.56	phospholipid-translocating P-type ATPase domain-containing protein (*T. equinum*)
TERG_02565	-1.56	sulfate transporter (*T. equinum*)
TERG_01127	-1.56	1,3-beta-glucan synthase component FKS1
TERG_02116	-1.57	PX domain-containing protein (*T. tonsurans*)
TERG_04743	-1.57	C6 finger domain protein, putative (*A. benhamiae*)
TERG_11541	-1.57	hypothetical protein
TERG_03492	-1.59	oxalate decarboxylase, putative (*A. benhamiae*)
TERG_11836	-1.59	choline transporter Hnm1, putative (*T. verrucosum*)

TERG_11845	-1.60	RNA polymerase II mediator complex component Srb8, putative (*T. verrucosum*)
TERG_03187	-1.60	aspartate aminotransferase (*T. tonsurans*)
TERG_05576	-1.61	cell wall glucanase (Scw11), putative (*A. benhamiae*)
TERG_06998	-1.61	3-ketoacyl-acyl carrier protein reductase (*T. tonsurans*)
TERG_11567	-1.62	hypothetical protein
TERG_11647	-1.62	phosphatidylserine decarboxylase
TERG_11730	-1.62	hypothetical protein
TERG_04594	-1.63	porin (*T. tonsurans*)
TERG_02385	-1.63	hypothetical protein
TERG_11955	-1.64	long-chain-fatty-acid-CoA ligase 1 (*M. gypseum*)
TERG_02566	-1.64	hypothetical protein
TERG_08938	-1.65	thermoresistant gluconokinase (*T. tonsurans*)
TERG_02567	-1.65	phytanoyl-CoA dioxygenase (*T. tonsurans*)
TERG_06994	-1.65	hypothetical protein
TERG_07266	-1.65	zinc finger protein 273 (*M. gypseum*)
TERG_11566	-1.65	hypothetical protein
TERG_08824	-1.65	hypothetical protein
TERG_00330	-1.67	hypothetical protein
TERG_02694	-1.67	sulfite efflux pump SSU1
TERG_03234	-1.67	DUF907 domain-containing protein (*T. equinum*)
TERG_00616	-1.67	potassium/sodium efflux P-type ATPase, fungal-type
TERG_03069	-1.67	hypothetical protein
TERG_01988	-1.67	F-box domain protein (*A. benhamiae*)
TERG_04206	-1.67	AT DNA binding protein (*T. equinum*)
TERG_07121	-1.68	hypothetical protein
TERG_06366	-1.68	CAMK protein kinase
TERG_05604	-1.68	C6 finger domain-containing protein (*T. equinum*)
TERG_04582	-1.68	extracellular cellulase CelA/allergen Asp F7-like, putative (*T. verrucosum*)
TERG_00730	-1.69	phospholipid metabolism enzyme regulator, putative (*T. verrucosum*)
TERG_02059	-1.69	AbpA protein (*T. equinum*)
TERG_03969	-1.69	hypothetical protein
TERG_06695	-1.70	hypothetical protein
TERG_01758	-1.70	C2 domain-containing protein (*T. tonsurans*)
TERG_00613	-1.70	hypothetical protein
TERG_11595	-1.72	LipA and NB-ARC domain-containing protein (*M. canis*)
TERG_07820	-1.72	phospholipase (*T. tonsurans*)
TERG_06779	-1.72	hypothetical protein
TERG_00853	-1.72	choline transporter (*T. equinum*)
TERG_00917	-1.72	aureobasidin A resistance protein (*T. equinum*)
TERG_03059	-1.72	hypothetical protein
TERG_06383	-1.75	homeobox domain-containing protein (*T. equinum*)
TERG_12724	-1.75	nonribosomal peptide synthase GliP (*A. benhamiae*)
TERG_01049	-1.75	hypothetical protein

TERG_06990	-1.75	hypothetical protein
TERG_07958	-1.75	myosin class II heavy chain (MHC), putative (*T. verrucosum*)
TERG_00863	-1.75	NsdD (*T. equinum*)
TERG_07469	-1.77	SUN domain-containing protein (*T. equinum*)
TERG_06290	-1.77	hypothetical protein
TERG_11648	-1.78	phosphatidylserine decarboxylase (*T. tonsurans*)
TERG_01397	-1.78	C2H2 finger domain-containing protein (*T. equinum*)
TERG_07391	-1.79	hypothetical protein
TERG_07720	-1.79	copper-transporting ATPase (*T. equinum*)
TERG_07681	-1.79	sexual development activator VeA (*T. verrucosum*)
TERG_12086	-1.80	hypothetical protein
TERG_05977	-1.80	hypothetical protein
TERG_01934	-1.80	C2H2 finger domain-containing protein (*T. equinum*)
TERG_01967	-1.81	GPI anchored protein, putative (*T. verrucosum*)
TERG_04324	-1.81	extracellular metalloproteinase 4
TERG_01898	-1.83	hypothetical protein
TERG_05885	-1.84	DnaJ domain-containing protein (*T. equinum*)
TERG_08825	-1.84	bZIP transcription factor JlbA/IDI-4 (*T. verrucosum*)
TERG_01012	-1.84	hypothetical protein
TERG_01749	-1.85	hypothetical protein
TERG_08140	-1.86	methyltransferase type 11 (*M. gypseum*)
TERG_06359	-1.87	hypothetical protein
TERG_02584	-1.87	hypothetical protein
TERG_01313	-1.87	C2H2 finger domain-containing protein (*T. equinum*)
TERG_02468	-1.88	ubiquitin thiolesterase (OtuB1), putative (*T. verrucosum*)
TERG_12645	-1.88	hypothetical protein
TERG_02185	-1.88	hypothetical protein
TERG_08405	-1.88	leucine aminopeptidase 2
TERG_00315	-1.88	RAN protein kinase
TERG_00694	-1.88	glutamate 5-kinase
TERG_11729	-1.89	hypothetical protein
TERG_03211	-1.90	actin cytoskeleton protein (*T. tonsurans*)
TERG_05626	-1.90	chitinase (*T. equinum*)
TERG_02891	-1.90	hypothetical protein
TERG_03334	-1.92	sensor histidine kinase/response regulator (*T. tonsurans*)
TERG_02593	-1.93	C2H2 finger domain protein, putative (*T. verrucosum*)
TERG_01851	-1.93	Pfs, NACHT and WD domain protein (*A. benhamiae*)
TERG_12126	-1.94	hypothetical protein
TERG_00981	-1.95	hypothetical protein
TERG_04925	-1.95	hypothetical protein
TERG_04278	-1.95	hypothetical protein
TERG_05824	-1.97	hypothetical protein
TERG_12640	-1.97	choriogenin Hminor (*M. canis*)

TERG_07931	-1.97	vesicle-mediated transport protein Vid24 (*T. equinum*)
TERG_06778	-1.98	PAP2 domain protein (*T. verrucosum*)
TERG_08298	-1.99	RTA1 domain-containing protein (*T. equinum*)
TERG_07968	-1.99	hypothetical protein
TERG_01985	-1.99	hypothetical protein
TERG_08191	-1.99	glucooligosaccharide oxidase (*T. equinum*)
TERG_03253	-2.00	hypothetical protein
TERG_01433	-2.01	WD domain, G-beta repeat protein (*A. benhamiae*)
TERG_06144	-2.01	cell wall serine-threonine-rich galactomannoprotein Mp1 (*A. benhamiae*)
TERG_08959	-2.01	hypothetical protein
TERG_00084	-2.01	chromosome segregation protein (Pcs1), putative (*T. verrucosum*)
TERG_03110	-2.02	GTPase activating protein (Gyp3), putative (*A. benhamiae*)
TERG_05492	-2.02	hypothetical protein
TERG_12646	-2.02	hypothetical protein
TERG_04877	-2.03	pH response protein PalF (*T. tonsurans*)
TERG_02800	-2.06	conidiation-specific protein 10
TERG_04862	-2.07	C6 transcription factor RosA (*A. benhamiae*)
TERG_05303	-2.09	phospholipase PldA, putative (*T. verrucosum*)
TERG_12371	-2.11	hypothetical protein
TERG_11506	-2.16	phosphoenolpyruvate carboxykinase [ATP]
TERG_08987	-2.17	hypothetical protein
TERG_08801	-2.19	hypothetical protein
TERG_00967	-2.19	hypothetical protein
TERG_01900	-2.21	aquaglyceroporin, putative (*A. benhamiae*)
TERG_02569	-2.22	conserved serine proline-rich protein (*A. benhamiae*)
TERG_08777	-2.23	hypothetical protein
TERG_04930	-2.25	glucose 1-dehydrogenase 2 (*M. gypseum*)
TERG_04146	-2.26	FAD/FMN-containing isoamyl alcohol oxidase MreA-like, putative (*A. benhamiae*)
TERG_00811	-2.26	C-x8-C-x5-C-x3-H type zinc finger protein (*T. equinum*)
TERG_12639	-2.26	choriogenin Hminor (*M. canis*)
TERG_04942	-2.27	hypothetical protein
TERG_06355	-2.28	acetyltransferase, GNAT family (*A. benhamiae*)
TERG_05490	-2.28	hypothetical protein
TERG_01869	-2.29	TOS1 (*T. equinum*)
TERG_04281	-2.40	woronin body major protein
TERG_02179	-2.41	keylime pathogenicity protein (*M. canis*)
TERG_00453	-2.42	PXA domain-containing protein (*T. tonsurans*)
TERG_11505	-2.43	phosphoenolpyruvate carboxykinase [ATP]
TERG_08503	-2.49	secalin (*M. canis*)
TERG_02814	-2.49	hypothetical protein
TERG_00517	-2.50	hypothetical protein
TERG_03771	-2.52	ankyrin repeat protein (*T. verrucosum*)
TERG_02384	-2.53	hypothetical protein

TERG_03966			-2.54	hypothetical protein
TERG_00765			-2.58	hypothetical protein
TERG_07199			-2.65	hypothetical protein
TERG_01578			-2.65	Pfs, NB-ARC and Ankyrin domain protein (*T. verrucosum*)
TERG_02208			-2.69	hypothetical protein
TERG_04183			-2.69	yqcI protein (*T. equinum*)
TERG_08709			-2.72	SesA protein (*T. equinum*)
TERG_04107			-2.74	FunK1 protein kinase (*M. canis*)
TERG_04228			-2.76	hypothetical protein
TERG_08961			-2.76	hypothetical protein
TERG_07282			-2.77	cytochrome P450 monooxygenase, putative (*T. verrucosum*)
TERG_05906			-2.79	serine/threonine protein kinase
TERG_04959			-2.85	hypothetical protein
TERG_03070			-2.93	flotillin domain-containing protein (*T. equinum*)
TERG_07234			-2.96	hypothetical protein
TERG_08121			-3.19	protein kinase subdomain-containing protein (*M. gypseum*)
TERG_03304			-3.33	AAA family ATPase, putative (*T. verrucosum*)
TERG_04610			-3.43	hypothetical protein
TERG_07486			-3.45	hypothetical protein
TERG_02650			-3.54	NmrA family protein (*T. equinum*)
TERG_02449			-3.55	phosphoadenosine phosphosulfate reductase
TERG_03251			-3.64	hypothetical protein
TERG_01956			-3.76	C2H2 finger domain protein, putative (*A. benhamiae*)
TERG_07042			-4.17	viral protein TPX (*M. canis*)
TERG_01853			-4.29	hypothetical protein
TERG_03252			-4.30	hypothetical protein

Gene expression values are expressed in log_2 fold change.

ID	24 hours	48 hours	96 hours	Gene Product Name
TERG_00598	5.25	4.93		hypothetical protein
TERG_04115	4.92	5.55		aldo-keto reductase, putative (*T. verrucosum*)
TERG_07166	4.50	3.79		hypothetical protein
TERG_08358	4.23	8.43		dimethylallyl tryptophan synthase GliD1 (*T. equinum*)
TERG_02820	4.15	2.76		polyketide synthase (*T. tonsurans*)
TERG_08700	4.07	2.69		DUF895 domain membrane protein (*T. equinum*)
TERG_06839	3.89	3.30		extracellular proline-rich protein (*T. verrucosum*)
TERG_01192	3.76	3.58		hypothetical protein
TERG_06673	3.58	3.49		pachytene checkpoint component Pch2 (*T. tonsurans*)
TERG_05259	3.31	1.80		cytochrome P450, putative (*A. benhamiae*)
TERG_02511	3.18	2.70		salicylate hydroxylase, putative (*A. benhamiae*)
TERG_07466	3.13	3.79		hypothetical protein
TERG_11582	3.09	3.52		hypothetical protein

TERG_08359	3.08	2.63	FAD-dependent monooxygenase (*T. tonsurans*)
TERG_12532	2.92	1.71	hypothetical protein
TERG_01053	2.88	2.88	MFS monocarboxylate transporter, putative (*T. verrucosum*)
TERG_05260	2.84	1.89	general amidase GmdB (*A. benhamiae*)
TERG_11866	2.80	2.30	hypothetical protein
TERG_11905	2.78	3.46	hypothetical protein
TERG_04732	2.75	2.48	hypothetical protein
TERG_08002	2.69	2.42	hypothetical protein
TERG_11660	2.64	2.22	mitochondrial 40S ribosomal protein (*T. tonsurans*)
TERG_07295	2.61	3.05	C6 finger domain protein, putative (*T. verrucosum*)
TERG_01443	2.57	2.67	ABC multidrug transporter (*T. tonsurans*)
TERG_05317	2.56	3.10	lipase, putative (*T. verrucosum*)
TERG_05442	2.49	2.96	hypothetical protein
TERG_11940	2.46	2.84	hypothetical protein
TERG_04461	2.39	1.52	P-type ATPase (*T. equinum*)
TERG_12222	2.38	3.01	polyketide synthase (*T. equinum*)
TERG_06933	2.37	2.19	hypothetical protein
TERG_06105	2.36	2.85	hypothetical protein
TERG_03916	2.35	1.82	geranylgeranyl pyrophosphate synthetase (*T. tonsurans*)
TERG_02307	2.35	1.63	mitotic spindle checkpoint protein (Mad2B), putative (*T. verrucosum*)
TERG_12306	2.31	1.66	autophagy protein (*T. tonsurans*)
TERG_03956	2.29	2.15	hypothetical protein
TERG_07959	2.23	1.94	GPI-anchored cell wall beta-1,3-endoglucanase EglC (*A. benhamiae*)
TERG_11881	2.21	1.80	G-patch domain-containing protein (*T. equinum*)
TERG_01444	2.21	1.69	nonribosomal peptide synthase Pes1 (*A. benhamiae*)
TERG_08614	2.20	3.65	hypothetical protein
TERG_03054	2.20	2.66	glycosyl hydrolase (*T. tonsurans*)
TERG_00914	2.17	2.58	hypothetical protein
TERG_03815	2.17	2.39	subtilisin-like protease 3
TERG_02118	2.15	2.48	GPI anchored protein, putative (*A. benhamiae*)
TERG_02469	2.15	1.68	hypothetical protein
TERG_03248	2.14	1.75	extracellular metalloproteinase 3
TERG_11655	2.13	3.24	hypothetical protein
TERG_11949	2.13	2.45	hypothetical protein
TERG_03614	2.12	1.57	hypothetical protein
TERG_02512	2.11	2.90	hypothetical protein
TERG_03706	2.10	1.97	trichodiene oxygenase (*T. equinum*)
TERG_00702	2.09	1.82	hypothetical protein
TERG_08208	2.09	1.55	glutathione S-transferase (*T. verrucosum*)
TERG_08913	2.09	1.69	cytochrome P450 (*T. equinum*)
TERG_03702	2.08	1.98	hypothetical protein
TERG_01092	2.07	2.92	leucine rich repeat protein (*T. equinum*)
TERG_11757	2.06	2.70	hypothetical protein

TERG_00520	2.06	2.57	hypothetical protein
TERG_04164	2.05	1.51	hypothetical protein
TERG_11832	2.03	2.35	cytosolic Fe-S cluster assembling factor NBP35 (*T. tonsurans*)
TERG_07761	1.99	2.23	hypothetical protein
TERG_08354	1.98	1.68	hypothetical protein
TERG_08115	1.97	3.67	hypothetical protein
TERG_11802	1.97	2.43	mucin (*M. canis*)
TERG_00828	1.97	2.55	hypothetical protein
TERG_00710	1.96	1.61	hypothetical protein
TERG_12540	1.95	1.95	hypothetical protein
TERG_00072	1.95	1.77	hypothetical protein
TERG_03893	1.95	2.70	C6 transcription factor, putative (*A. benhamiae*)
TERG_06106	1.94	1.83	sulfate permease 2 (*T. tonsurans*)
TERG_12368	1.94	2.21	hypothetical protein
TERG_02092	1.94	1.95	hypothetical protein
TERG_01223	1.93	3.48	F-box domain protein (*T. verrucosum*)
TERG_01923	1.91	2.14	hypothetical protein
TERG_03886	1.91	2.57	proline permease, putative (*T. verrucosum*)
TERG_02661	1.90	2.07	hypothetical protein
TERG_07399	1.89	1.94	fibronectin type III domain-containing protein (*M. gypseum*)
TERG_08135	1.88	1.84	GTP-binding protein GTR2 (*M. gypseum*)
TERG_06377	1.88	2.38	metalloproteinase, putative (*A. benhamiae*)
TERG_02405	1.87	1.55	mating locus protein, putative (*T. verrucosum*)
TERG_06512	1.87	3.22	hypothetical protein
TERG_04956	1.86	1.92	hypothetical protein
TERG_06073	1.82	1.75	MFS monocarboxylate transporter (*T. equinum*)
TERG_08114	1.76	3.03	anaphase-promoting complex component Cut20/Apc4 (*T. tonsurans*)
TERG_01768	1.76	1.81	hypothetical protein
TERG_02487	1.75	1.59	NADPH oxidase isoform 2 (*T. equinum*)
TERG_06691	1.71	1.61	hypothetical protein
TERG_08334	1.70	2.19	proline oxidase Put1, putative (*A. benhamiae*)
TERG_04187	1.70	1.52	hypothetical protein
TERG_11694	1.69	2.08	hypothetical protein
TERG_08049	1.69	1.52	cation diffusion facilitator, putative (*T. verrucosum*)
TERG_03705	1.68	2.16	cytochrome P450 monooxygenase, putative (*A. benhamiae*)
TERG_03554	1.68	1.78	hypothetical protein
TERG_03024	1.67	2.26	hypothetical protein
TERG_01609	1.66	1.62	Na/K ATPase alpha 1 subunit (*T. equinum*)
TERG_03052	1.65	2.41	glycosyl hydrolase (*T. tonsurans*)
TERG_12603	1.64	1.99	hypothetical protein
TERG_05117	1.61	1.82	transcription factor TFIIH subunit Tfb4 (*T. tonsurans*)
TERG_06565	1.60	1.55	protein kinase subdomain-containing protein (*T. equinum*)
TERG_03949	1.55	1.96	equisetin synthetase (*T. equinum*)

TERG_02422	1.53	2.03	rho GTPase activator (*T. equinum*)
TERG_03846	1.52	1.67	F-box domain-containing protein (*T. equinum*)
TERG_05130	1.51	1.85	thioredoxin reductase GliT (*A. benhamiae*)
TERG_01510	1.51	2.16	proline-specific permease (*T. tonsurans*)
TERG_07552	1.51	1.57	hypothetical protein
TERG_00831	-1.53	-2.04	gamma-glutamyltransferase
TERG_01843	-1.58	-1.99	inositol-3-phosphate synthase (*T. tonsurans*)
TERG_00956	-1.59	-3.10	extracellular thaumatin domain-containing protein (*T. equinum*)
TERG_04666	-1.63	-1.94	hypothetical protein
TERG_03352	-1.64	-1.73	tyrosine decarboxylase (*T. equinum*)
TERG_07923	-1.66	-1.75	neutral amino acid permease (*T. equinum*)
TERG_02454	-1.67	-1.79	deoxyribose-phosphate aldolase
TERG_07417	-1.67	-1.92	hypothetical protein
TERG_00070	-1.68	-2.00	maleylacetoacetate isomerase
TERG_12027	-1.68	-1.55	hypothetical protein
TERG_01518	-1.69	-1.67	hypothetical protein
TERG_01190	-1.71	-1.74	RNA binding protein (*T. equinum*)
TERG_06975	-1.72	-1.92	S-formylglutathione hydrolase
TERG_12397	-1.73	-1.61	hypothetical protein
TERG_07751	-1.74	-1.82	serine/threonine protein kinase (*T. equinum*)
TERG_12681	-1.74	-1.84	alpha-mannosidase (*T. tonsurans*)
TERG_05201	-1.76	-1.80	integral membrane protein (*T. equinum*)
TERG_01138	-1.77	-2.30	monocarboxylate transporter 4 (*M. gypseum*)
TERG_01383	-1.78	-1.96	dienelactone hydrolase (*T. tonsurans*)
TERG_04680	-1.81	-1.65	hypothetical protein
TERG_07927	-1.81	-2.16	hypothetical protein
TERG_11786	-1.82	-2.27	oligosaccharyl transferase stt3 subunit (*T. equinum*)
TERG_06651	-1.82	-1.62	cytochrome P450, putative (*T. verrucosum*)
TERG_11564	-1.83	-1.87	beta-lactamase (*T. equinum*)
TERG_08787	-1.84	-2.72	aminotransferase (*T. tonsurans*)
TERG_00342	-1.84	-1.75	phosphoglucomutase
TERG_04715	-1.85	-1.96	hypothetical protein
TERG_04021	-1.86	-2.08	GNAT family acetyltransferase, putative (*A. benhamiae*)
TERG_03291	-1.88	-1.67	SNF2 family helicase, putative (*T. verrucosum*)
TERG_00878	-1.92	-2.43	hypothetical protein
TERG_04721	-1.92	-1.92	glutamate carboxypeptidase (*T. equinum*)
TERG_11942	-1.98	-1.58	MFS maltose permease (*T. tonsurans*)
TERG_00385	-1.99	-2.58	oxidoreductase (*T. equinum*)
TERG_07777	-2.00	-2.62	O-acetylhomoserine (thiol)-lyase
TERG_00510	-2.01	-1.66	hypothetical protein
TERG_06466	-2.01	-1.90	hypothetical protein
TERG_03367	-2.03	-1.98	hypothetical protein
TERG_02001	-2.03	-2.74	oligopeptidase (*T. tonsurans*)
TERG_03174	-2.04	-2.12	MFS siderochrome iron transporter MirB (*T. verrucosum*)

TERG_02493	-2.12	-1.93	glyoxalase family protein (*T. verrucosum*)
TERG_02545	-2.13	-1.95	MFS monocarboxylate transporter (*T. equinum*)
TERG_08757	-2.14	-3.34	hypothetical protein
TERG_02649	-2.19	-2.83	benzoate 4-monooxygenase cytochrome P450 (*T. equinum*)
TERG_02960	-2.23	-2.61	Beta-ketoacyl synthase (*T. tonsurans*)
TERG_11593	-2.24	-2.04	carboxypeptidase Y, putative (*A. benhamiae*)
TERG_06208	-2.27	-2.00	DUF636 domain-containing protein (*M. gypseum*)
TERG_00151	-2.30	-2.02	hypothetical protein
TERG_00995	-2.30	-1.76	hypothetical protein
TERG_00807	-2.32	-1.75	hypothetical protein
TERG_04227	-2.33	-2.26	ABC transporter (*T. tonsurans*)
TERG_07898	-2.43	-1.79	lanthionine synthetase C family protein, putative (*T. verrucosum*)
TERG_05771	-2.43	-2.09	hypothetical protein
TERG_05095	-2.46	-1.80	quinone oxidoreductase (*T. tonsurans*)
TERG_02725	-2.57	-2.61	hypothetical protein
TERG_00499	-2.58	-1.97	hypothetical protein
TERG_01886	-2.62	-2.33	hypothetical protein
TERG_04378	-2.67	-2.83	aldehyde reductase I (ARI), putative (*T. verrucosum*)
TERG_05032	-2.77	-2.20	C6 transcription factor (*T. equinum*)
TERG_00911	-2.86	-2.85	hypothetical protein
TERG_04639	-2.89	-3.17	hypothetical protein
TERG_04879	-3.08	-1.53	hypothetical protein
TERG_03299	-3.30	-1.58	pyridoxal-dependent decarboxylase (*T. equinum*)
TERG_05299	-3.32	-2.75	glutathione S-transferase, putative (*A. benhamiae*)
TERG_02961	-3.46	-3.50	MFS transporter (*T. equinum*)
TERG_04250	-3.75	-2.41	enoyl-CoA hydratase/isomerase family protein (*A. benhamiae*)
TERG_08054	-3.90	-3.46	homoserine acetyltransferase (*T. tonsurans*)
TERG_00026	-4.09	-2.95	flavin-binding monooxygenase, putative (*T. verrucosum*)
TERG_03699	-4.17	-2.91	hypothetical protein
TERG_04310	-4.23	-2.89	alcohol dehydrogenase (*T. tonsurans*)
TERG_07133	-4.25	-3.79	hypothetical protein
TERG_06052	-4.31	-3.26	oxidoreductase, short-chain dehydrogenase/reductase family (*A. benhamiae*)
TERG_07326	-4.77	-2.43	glutathione S-transferase PARB (*T. equinum*)
TERG_03008	-5.19	-2.46	zinc-containing alcohol dehydrogenase, putative (*A. benhamiae*)
TERG_06051	-5.77	-6.03	4-dimethylallyltryptophan methyltransferase (*T. tonsurans*)
TERG_04738	-8.64	-4.98	hypothetical protein

Gene expression values are expressed in $\log_2$ fold change.

ID	24 hours	48 hours	96 hours	Gene Product Name
TERG_07456	3.21		2.31	cell wall protein PhiA (*T. equinum*)
TERG_05733	3.02		-6.75	salivary glue protein Sgs-3 (*M. canis*)

TERG_05965	2.88	3.56	hypothetical protein
TERG_05323	2.39	2.24	hypothetical protein
TERG_01178	2.12	2.04	hypothetical protein
TERG_04156	2.11	2.90	hypothetical protein
TERG_03647	2.08	1.92	hypothetical protein
TERG_04547	2.03	2.05	aquaporin (*T. tonsurans*)
TERG_11709	1.83	2.36	hypothetical protein
TERG_12061	1.74	2.14	cytochrome P450 monooxygenase (*T. equinum*)
TERG_02583	1.56	-3.24	phosphate permease (*A. benhamiae*)
TERG_02447	-1.59	-2.55	sulfate adenylyltransferase
TERG_04508	-1.62	-2.04	AAA ATPase (*T. equinum*)
TERG_11844	-1.67	-1.62	RNA polymerase II mediator complex component Srb8 (*T. equinum*)
TERG_00388	-1.68	-1.85	DnaJ domain-containing protein (*T. equinum*)
TERG_03254	-1.78	-2.70	hypothetical protein
TERG_04130	-1.81	-2.21	plasma membrane ATPase
TERG_08436	-1.81	-1.78	hypothetical protein
TERG_08708	-1.87	-2.52	SesA protein (*T. equinum*)
TERG_02209	-1.94	-2.12	deoxyribodipyrimidine photo-lyase (*T. tonsurans*)
TERG_01685	-2.00	-2.92	glutathione S-transferase, putative (*A. benhamiae*)
TERG_05035	-2.08	-1.72	pentatricopeptide repeat protein (*T. tonsurans*)
TERG_07504	-5.49	-6.15	sialidase (*T. tonsurans*)

Gene expression values are expressed in $\log_2$ fold change.

ID	24 hours	48 hours	96 hours	Gene Product Name
TERG_12391		4.19	4.94	hypothetical protein
TERG_00060		3.63	3.30	GPI anchored cell wall protein, putative (*A. benhamiae*)
TERG_05523		3.29	2.15	hypothetical protein
TERG_04511		3.10	1.70	hypothetical protein
TERG_00221		3.07	2.58	hypothetical protein
TERG_11516		2.65	2.32	hypothetical protein
TERG_06116		2.31	1.77	indoleamine 2,3-dioxygenase (*T. equinum*)
TERG_01405		2.25	2.30	glutathione S-transferase (*T. equinum*)
TERG_12064		2.25	1.99	hypothetical protein
TERG_12028		2.24	2.11	metallothionein family protein (*T. equinum*)
TERG_01841		2.21	2.57	hypothetical protein
TERG_11915		2.20	2.12	nucleolus protein required for cell viability (*T. tonsurans*)
TERG_04504		2.19	-2.01	extracellular protein (*T. equinum*)
TERG_04032		2.17	1.51	DNA repair and transcription factor Ada, putative (*A. benhamiae*)
TERG_01840		2.15	3.01	carboxypeptidase S1 (*T. equinum*)
TERG_12158		1.98	1.74	hypothetical protein
TERG_06035		1.92	4.44	hypothetical protein
TERG_12141		1.88	1.92	Extracellular lipase (*T. tonsurans*)

TERG_06080	1.82	2.00	serine/threonine protein kinase
TERG_12664	1.81	1.94	adenosine deaminase (*T. tonsurans*)
TERG_04778	1.80	2.06	ribosome assembly and transport protein Srp40, putative (*T. verrucosum*)
TERG_12097	1.76	2.00	cytochrome P450 monooxygenase, putative (*A. benhamiae*)
TERG_00471	1.73	-1.89	hypothetical protein
TERG_01757	1.71	2.68	DNA repair protein RAD16 (*T. equinum*)
TERG_08145	1.66	2.12	ADP-ribose 1''-phosphate phosphatase (*T. equinum*)
TERG_12327	1.63	2.37	alkaline serine protease (PR1)/allergen F18, putative (*T. verrucosum*)
TERG_05195	1.62	2.83	radical SAM domain-containing protein (*T. tonsurans*)
TERG_07053	1.61	1.59	hypothetical protein
TERG_03473	1.57	-1.59	hypothetical protein
TERG_04924	1.56	2.27	threonine aldolase, putative (*T. verrucosum*)
TERG_12085	1.56	-2.01	hypothetical protein
TERG_06859	1.51	1.70	exosome complex endonuclease 1/ribosomal RNA processing protein (*T. equinum*)
TERG_06995	-1.52	-2.71	metalloreductase, putative (*T. verrucosum*)
TERG_03116	-1.52	-1.54	zf-PARP-type zinc finger protein (*T. tonsurans*)
TERG_07904	-1.53	-1.71	tubulin beta chain
TERG_00615	-1.53	-1.82	HLH transcription factor, putative (*A. benhamiae*)
TERG_04937	-1.54	-1.57	alpha/beta hydrolase (*T. equinum*)
TERG_11640	-1.55	-2.61	hypothetical protein
TERG_07905	-1.57	-1.73	1-pyrroline-5-carboxylate dehydrogenase
TERG_06177	-1.60	-1.91	mitochondrial carrier protein, putative (*T. verrucosum*)
TERG_01925	-1.60	-2.02	hypothetical protein
TERG_07785	-1.61	-1.56	SNARE domain-containing protein (*T. tonsurans*)
TERG_02353	-1.62	-3.51	UbiD family decarboxylase, putative (*T. verrucosum*)
TERG_01904	-1.63	-1.62	hypothetical protein
TERG_06438	-1.64	-1.87	malate dehydrogenase, NAD-dependent
TERG_05651	-1.64	-1.78	hypothetical protein
TERG_03183	-1.66	-2.26	hypothetical protein
TERG_06207	-1.67	-1.59	proline oxidase PrnD (*T. verrucosum*)
TERG_05516	-1.68	-2.16	RNA interference and gene silencing protein (Qde2), putative (*T. verrucosum*)
TERG_03033	-1.68	-2.03	BTB domain and ankyrin repeat protein (*A. benhamiae*)
TERG_08560	-1.70	-1.55	hypothetical protein
TERG_08459	-1.71	-2.19	cell wall glucanase, putative (*T. verrucosum*)
TERG_02489	-1.72	-1.70	sister chromatid cohesion acetyltransferase Eco1, putative (*A. benhamiae*)
TERG_11815	-1.74	-2.30	OPT family small oligopeptide transporter
TERG_11835	-1.74	-1.53	choline transporter Hnm1, putative (*T. verrucosum*)
TERG_05793	-1.75	-1.63	hypothetical protein
TERG_01733	-1.75	-1.59	alcohol dehydrogenase, putative (*T. verrucosum*)
TERG_05616	-1.77	-2.03	hypothetical protein
TERG_07260	-1.80	-2.15	hypothetical protein

TERG_00724		-1.90	-2.54	C6 finger domain protein, putative (*T. verrucosum*)
TERG_01848		-1.91	-2.92	integral membrane protein (*M. gypseum*)
TERG_01336		-1.91	-2.15	MFS transporter (*T. equinum*)
TERG_05518		-1.92	-2.72	oxidoreductase, short chain dehydrogenase/reductase family (*A. benhamiae*)
TERG_06666		-1.92	-1.78	prolyl aminopeptidase
TERG_06607		-1.92	-2.20	serine-rich protein, putative (*T. verrucosum*)
TERG_07580		-1.95	-1.57	hypothetical protein
TERG_03728		-1.99	-3.16	hypothetical protein
TERG_03929		-1.99	-3.18	hypothetical protein
TERG_05615		-2.00	-5.55	hsp70-like protein (*T. equinum*)
TERG_00951		-2.01	-3.84	hypothetical protein
TERG_01901		-2.10	-2.99	glycerol kinase
TERG_04775		-2.11	-1.75	beta-N-hexosaminidase, putative (*A. benhamiae*)
TERG_01563		-2.15	-1.74	hypothetical protein
TERG_04079		-2.19	-1.69	4-aminobutyrate aminotransferase
TERG_00731		-2.21	-3.21	hypothetical protein
TERG_04867		-2.28	-3.57	SAM and PH domain protein (Boi1), putative (*A. benhamiae*)
TERG_02542		-2.29	-1.81	integral membrane protein Pth11-like, putative (*T. verrucosum*)
TERG_06189		-2.32	-1.99	endo-1,3(4)-beta-glucanase, putative (*T. verrucosum*)
TERG_01281		-2.33	-2.12	malate synthase, glyoxysomal
TERG_00958		-2.38	-1.59	amino acid permease (*T. tonsurans*)
TERG_02798		-2.41	-2.26	DUF895 domain membrane protein (*T. equinum*)
TERG_07294		-2.41	-1.90	glyoxylate reductase (*T. tonsurans*)
TERG_02845		-2.43	-1.85	cercosporin toxin biosynthesis protein (*T. equinum*)
TERG_05895		-2.44	-1.87	MFS transporter (*M. canis*)
TERG_12498		-2.44	-2.10	2-deoxy-D-gluconate 3-dehydrogenase (*T. equinum*)
TERG_08712		-2.48	-2.51	betaine lipid synthase (*T. tonsurans*)
TERG_00481		-2.60	-2.57	beta-glucosidase, putative (*T. verrucosum*)
TERG_05287		-2.61	-2.31	glycerate-and formate-dehydrogenase (*T. equinum*)
TERG_07783		-2.70	-3.35	MFS peptide transporter, putative (*T. verrucosum*)
TERG_03072		-2.73	-1.51	F-box domain-containing protein (*T. equinum*)
TERG_02538		-2.83	-1.64	carboxylesterase (*T. equinum*)
TERG_02528		-2.89	-2.53	ubiE/COQ5 methyltransferase, putative (*T. verrucosum*)
TERG_02380		-4.05	-5.78	hypothetical protein

Gene expression values are expressed in $\log_2$ fold change.

ID	24 hours	48 hours	96 hours	Gene Product Name
TERG_07028	6.46	7.07	6.69	hypothetical protein
TERG_01004	4.30	3.66	3.11	extracellular sialidase/neuraminidase (*T. equinum*)
TERG_12090	4.06	2.83	3.73	hypothetical protein
TERG_08360	3.93	3.45	2.02	metallo-beta-lactamase superfamily protein (*T. equinum*)
TERG_00161	3.58	4.56	3.41	hypothetical protein

TERG_03827	3.43	1.87	2.16	hypothetical protein
TERG_03826	3.42	2.14	2.13	hypothetical protein
TERG_06601	3.26	4.39	3.65	flavin-containing amine oxidasedehydrogenase (*T. equinum*)
TERG_07847	2.62	1.89	1.87	hypothetical protein
TERG_04569	2.42	2.21	1.95	cytosolic Fe-S cluster assembling factor cfd1 (*T. equinum*)
TERG_08077	2.32	1.62	1.99	hypothetical protein
TERG_11517	2.25	3.17	1.95	hypothetical protein
TERG_06076	2.23	2.42	2.73	hypothetical protein
TERG_03620	2.18	2.15	2.82	glycosyl transferase (*T. tonsurans*)
TERG_07364	2.00	2.20	2.32	oxidoreductase, zinc-binding dehydrogenase family superfamily (*T. verrucosum*)
TERG_05374	1.97	1.59	1.66	hypothetical protein
TERG_00371	1.79	2.86	1.51	ferric-chelate reductase (*T. tonsurans*)
TERG_12320	1.78	1.50	1.91	hypothetical protein
TERG_08149	1.71	1.78	1.51	hypothetical protein
TERG_00119	1.70	2.00	1.61	CAMK/CAMKL/GIN4 protein kinase
TERG_00709	1.69	1.88	2.17	MOSC domain-containing protein (*T. equinum*)
TERG_00713	1.60	1.77	2.16	hypothetical protein
TERG_00879	-1.57	-2.23	-2.82	hypothetical protein
TERG_03353	-1.58	-2.95	-4.06	endoglucanase (*T. equinum*)
TERG_07135	-1.58	-1.75	-1.67	hypothetical protein
TERG_05659	-1.58	-2.67	-3.48	hypothetical protein
TERG_01269	-1.58	-1.69	-1.56	2-methylcitrate dehydratase
TERG_03182	-1.58	-1.53	-2.03	serine/threonine protein kinase (*T. equinum*)
TERG_02210	-1.59	-2.62	-2.34	hypothetical protein
TERG_00538	-1.59	-2.24	-1.84	oxidoreductase (*T. tonsurans*)
TERG_00733	-1.64	-2.66	-4.27	hypothetical protein
TERG_00953	-1.65	-1.86	-2.22	OefC protein (*T. equinum*)
TERG_05540	-1.67	-1.83	-3.10	cytochrome P450 monooxygenase, putative (*T. verrucosum*)
TERG_08178	-1.69	-3.22	-2.19	endoglucanase (*T. equinum*)
TERG_03544	-1.70	-1.74	-3.13	CMGC/RCK/MAK protein kinase
TERG_05899	-1.73	-2.85	-2.72	ubiE/COQ5 methyltransferase, putative (*A. benhamiae*)
TERG_06238	-1.76	-2.05	-1.66	dihydroxy-acid dehydratase
TERG_02702	-1.76	-2.42	-2.13	sphingoid long-chain base transporter RSB1 (*T. equinum*)
TERG_02349	-1.77	-1.88	-1.92	hypothetical protein
TERG_07131	-1.81	-1.98	-1.71	phosphotransmitter protein Ypd1 (*T. equinum*)
TERG_00819	-1.83	-2.93	-2.90	PE repeat family protein (*T. verrucosum*)
TERG_02809	-1.85	-2.50	-2.39	hypothetical protein
TERG_03977	-1.87	-2.77	-2.68	hypothetical protein
TERG_01579	-1.88	-2.41	-3.69	hypothetical protein
TERG_07238	-1.89	-2.03	-1.53	zinc-binding oxidoreductase, putative (*A. benhamiae*)
TERG_05780	-1.90	-2.01	-1.76	hypothetical protein
TERG_02043	-1.91	-2.66	-2.98	cysteine desulfurase (*T. equinum*)

TERG_07796	-1.94	-3.95	-1.93	oxidoreductase, short-chain dehydrogenase/reductase family (*A. benhamiae*)
TERG_00855	-1.94	-4.13	-3.61	aromatic amino acid aminotransferase, putative (*A. benhamiae*)
TERG_02587	-1.95	-3.37	-2.24	hypothetical protein
TERG_05063	-1.95	-2.74	-3.28	extracellular serine-rich protein (*T. verrucosum*)
TERG_04487	-1.95	-2.30	-2.13	hypothetical protein
TERG_11981	-1.97	-1.95	-1.98	3-ketosteroid dehydrogenase (*T. tonsurans*)
TERG_05632	-1.97	-2.91	-2.51	MFS transporter, putative (*T. verrucosum*)
TERG_05639	-1.98	-2.44	-2.23	hypothetical protein
TERG_00135	-2.00	-2.38	-1.96	metalloreductase transmembrane component (*T. equinum*)
TERG_01193	-2.00	-2.83	-3.14	PRO41 protein (*T. equinum*)
TERG_02986	-2.00	-1.69	-3.83	hypothetical protein
TERG_02368	-2.02	-2.53	-1.98	extracellular developmental signal biosynthesis protein FluG (*A. benhamiae*)
TERG_01489	-2.03	-2.17	-3.34	MFS transporter (*T. tonsurans*)
TERG_06801	-2.03	-1.80	-2.83	cyclic nucleotide-binding domain-containing protein (*T. tonsurans*)
TERG_02369	-2.04	-2.29	-1.93	MFS transporter (*T. tonsurans*)
TERG_00458	-2.05	-2.58	-2.43	catalytic/hydrolase (*T. equinum*)
TERG_07588	-2.11	-1.76	-1.64	5-methyltetrahydropteroyltriglutamate-homocysteine S-methyltransferase
TERG_03382	-2.12	-1.83	-1.62	ThiJ/PfpI family protein (*A. benhamiae*)
TERG_00854	-2.13	-2.66	-1.89	hypothetical protein
TERG_02615	-2.18	-2.87	-1.92	oxidoreductase, 2OG-Fe(II) oxygenase family, putative (*A. benhamiae*)
TERG_07830	-2.18	-3.07	-2.47	hypothetical protein
TERG_00875	-2.19	-1.79	-1.90	glutamate-cysteine ligase
TERG_12723	-2.20	-2.48	-2.17	nonribosomal peptide synthase GliP (*A. benhamiae*)
TERG_08353	-2.28	-2.83	-2.49	cytochrome P450 55A3 (*T. tonsurans*)
TERG_02199	-2.29	-2.79	-2.05	glutamate carboxypeptidase (*T. equinum*)
TERG_02262	-2.33	-2.17	-1.93	NACHT and WD domain protein (*T. verrucosum*)
TERG_03221	-2.34	-2.52	-1.97	C6 transcription factor, putative (*A. benhamiae*)
TERG_05808	-2.42	-2.66	-2.37	hypothetical protein
TERG_01959	-2.45	-2.69	-4.40	RNA polymerase Rpb1 C-terminal repeat domain-containing protein (*T. equinum*)
TERG_08174	-2.47	-3.00	-1.64	hypothetical protein
TERG_11563	-2.53	-2.36	-1.63	beta-lactamase (*T. equinum*)
TERG_04022	-2.59	-2.63	-2.20	carboxypeptidase S1, putative (*A. benhamiae*)
TERG_04563	-2.60	-1.63	-4.05	phenol monooxygenase (*T. tonsurans*)
TERG_02263	-2.67	-2.71	-1.95	hypothetical protein
TERG_02367	-2.69	-3.50	-3.17	hypothetical protein
TERG_06854	-2.76	-4.90	-3.33	glutamine-serine-proline rich protein, putative (*A. benhamiae*)
TERG_08869	-2.76	-3.63	-4.31	hypothetical protein
TERG_02197	-2.77	-3.10	-2.96	nitrilase, putative (*T. verrucosum*)
TERG_07039	-2.79	-2.54	-2.62	hypothetical protein
TERG_12591	-2.89	-3.07	-1.76	peptidase S8 family protein (*T. equinum*)
TERG_03359	-2.95	-1.64	-1.98	hypothetical protein

TERG_04421	-2.96	-3.51	-2.38	hypothetical protein
TERG_01490	-2.98	-1.64	-3.06	LysR family regulatory protein (*M. gypseum*)
TERG_11964	-3.02	-3.16	-2.20	C6 transcription factor, putative (*T. verrucosum*)
TERG_03160	-3.05	-3.11	-1.73	hypothetical protein
TERG_03148	-3.08	-4.38	-3.23	molybdenum cofactor sulfurase (*T. equinum*)
TERG_08868	-3.09	-3.67	-4.73	FKBP-type peptidyl-prolyl isomerase, putative (*A. benhamiae*)
TERG_03885	-3.15	-4.04	-3.46	hypothetical protein
TERG_04240	-3.18	-3.10	-1.54	cupin 2 domain-containing protein (*T. equinum*)
TERG_12321	-3.19	-2.64	-2.75	hypothetical protein
TERG_01360	-3.21	-2.51	-1.99	C6 transcription factor, putative (*T. verrucosum*)
TERG_12474	-3.26	-3.30	-2.12	ABC transporter (*T. tonsurans*)
TERG_08261	-3.27	-3.53	-3.75	glutamate decarboxylase
TERG_08264	-3.30	-2.57	-1.55	L-xylulose reductase
TERG_12504	-3.45	-3.64	-2.89	MFS multidrug transporter (*T. equinum*)
TERG_02365	-3.50	-3.81	-3.32	hypothetical protein
TERG_01981	-3.58	-3.02	-2.52	hypothetical protein
TERG_05445	-3.58	-3.95	-7.99	DUF323 domain-containing protein (*T. equinum*)
TERG_12503	-3.68	-3.46	-2.79	polyamine transporter 2
TERG_06484	-3.79	-5.51	-4.94	MYB DNA-binding domain-containing protein (*T. tonsurans*)
TERG_11630	-3.83	-3.84	-4.54	hypothetical protein
TERG_00483	-3.89	-3.61	-3.04	hypothetical protein
TERG_02843	-3.93	-3.38	-3.09	C6 transcription factor (*T. equinum*)
TERG_05943	-3.94	-4.47	-4.42	aminotransferase, class III (*T. verrucosum*)
TERG_12475	-4.08	-3.55	-2.49	ABC transporter, putative (*A. benhamiae*)
TERG_00856	-4.14	-5.31	-3.84	phytanoyl-CoA dioxygenase (*T. equinum*)
TERG_06802	-4.34	-4.00	-4.52	hypothetical protein
TERG_03314	-4.46	-5.08	-5.11	TfdA family oxidoreductase, putative (*T. verrucosum*)
TERG_01279	-4.48	-5.65	-5.17	flavin-binding monooxygenase (*T. equinum*)
TERG_01599	-4.51	-5.36	-4.71	hypothetical protein
TERG_02132	-4.53	-5.03	-4.95	5-histidylcysteine sulfoxide synthase
TERG_02133	-4.68	-3.92	-3.91	fluG protein (*T. equinum*)
TERG_00714	-4.73	-4.98	-5.47	cell pattern formation-associated protein (*T. tonsurans*)
TERG_00852	-4.77	-4.55	-3.59	Phytanoyl-CoA dioxygenase PhyH (*T. equinum*)
TERG_06055	-5.01	-4.93	-1.96	NmrA-like family protein (*A. benhamiae*)
TERG_04282	-5.16	-7.00	-5.57	GPI ethanolamine phosphate transferase (*T. tonsurans*)
TERG_12156	-5.27	-4.48	-3.76	hypothetical protein
TERG_02844	-5.43	-4.79	-3.50	major facilitator superfamily transporter (*T. tonsurans*)
TERG_08444	-5.55	-6.11	-2.74	CipC protein (*M. canis*)
TERG_06054	-5.59	-6.14	-2.08	hypothetical protein
TERG_04028	-5.62	-6.11	-4.15	cytochrome P450, putative (*T. verrucosum*)
TERG_03161	-6.30	-7.26	-5.86	hypothetical protein
TERG_06267	-6.52	-8.01	-8.73	hypothetical protein
TERG_02842	-6.86	-5.41	-4.31	6-hydroxy-D-nicotine oxidase (*T. equinum*)

TERG_02134	-7.13	-6.28	-5.49	indoleamine 2,3-dioxygenase (*T. equinum*)
TERG_02412	-7.24	-4.40	-2.49	HHE domain protein (*T. verrucosum*)
TERG_06049	-8.17	-6.83	-5.95	dimethylallyl tryptophan synthase, putative (*T. verrucosum*)

Gene expression values are expressed in log_2 fold change.

Δ*stuA* vs. wild type (Keratin)

ID	24 hours	48 hours	96 hours	Gene Product Name
TERG_05733	6.08			salivary glue protein Sgs-3 (*M. canis*)
TERG_12089	4.72			cytochrome P450, putative (*T. verrucosum*)
TERG_12088	4.24			cytochrome P450 (*T. equinum*)
TERG_01393	3.23			hypothetical protein
TERG_12198	3.15			hypothetical protein
TERG_06839	3.14			extracellular proline-rich protein (*T. verrucosum*)
TERG_05157	3.02			hypothetical protein
TERG_00736	2.89			alpha/beta hydrolase, putative (*A. benhamiae*)
TERG_06499	2.87			hypothetical protein
TERG_05443	2.79			hypothetical protein
TERG_06445	2.53			hypothetical protein
TERG_08837	2.49			hypothetical protein
TERG_07490	2.41			secreted protein (*M. gypseum*)
TERG_00575	2.39			F-box domain protein (*T. verrucosum*)
TERG_03837	2.37			threo-3-hydroxyaspartate ammonia-lyase (*T. equinum*)
TERG_06933	2.35			hypothetical protein
TERG_08077	2.34			hypothetical protein
TERG_03255	2.31			hypothetical protein
TERG_05310	2.28			kynurenine 3-monooxygenase (*T. tonsurans*)
TERG_04951	2.26			U-box domain-containing protein (*T. equinum*)
TERG_05851	2.24			hypothetical protein
TERG_04937	2.23			alpha/beta hydrolase (*T. equinum*)
TERG_08700	2.21			DUF895 domain membrane protein (*T. equinum*)
TERG_07544	2.19			lipase (*T. tonsurans*)
TERG_08211	2.17			acetyltransferase, GNAT family, putative (*A. benhamiae*)
TERG_01594	2.04			hypothetical protein
TERG_04200	2.01			hypothetical protein
TERG_01782	2.00			hypothetical protein
TERG_04580	1.97			NADP-specific glutamate dehydrogenase
TERG_12114	1.96			hypothetical protein
TERG_00967	1.94			hypothetical protein
TERG_02206	1.94			ankyrin repeat-containing protein (*T. equinum*)
TERG_03208	1.91			hypothetical protein
TERG_01790	1.90			meiotic RNA-binding protein 1 (*A. benhamiae*)
TERG_03857	1.88			phosphotransferase enzyme family protein (*T. equinum*)

TERG_04939	1.84	hypothetical protein
TERG_06533	1.84	glyoxalase family protein (*A. benhamiae*)
TERG_03473	1.83	hypothetical protein
TERG_01240	1.80	secreted protein (*T. equinum*)
TERG_04117	1.76	tannase, putative (*A. benhamiae*)
TERG_02511	1.72	salicylate hydroxylase, putative (*A. benhamiae*)
TERG_11849	1.72	hypothetical protein
TERG_08400	1.69	hypothetical protein
TERG_02745	1.66	phosphoglycerate dehydrogenase
TERG_08347	1.66	cytochrome P450 monooxygenase, putative (*A. benhamiae*)
TERG_06509	1.66	glutamate synthase (*T. tonsurans*)
TERG_07479	1.65	hypothetical protein
TERG_07824	1.65	BRCT domain-containing protein (*T. equinum*)
TERG_12368	1.62	hypothetical protein
TERG_02838	1.62	hypothetical protein
TERG_05429	1.60	MFS multidrug transporter, putative (*T. verrucosum*)
TERG_08334	1.59	proline oxidase Put1, putative (*A. benhamiae*)
TERG_07683	1.57	hypothetical protein
TERG_08982	1.57	hypothetical protein
TERG_04146	1.55	FAD/FMN-containing isoamyl alcohol oxidase MreA-like, putative (*A. benhamiae*)
TERG_08502	1.54	peptidyl-prolyl cis-trans isomerase-like 2
TERG_03986	1.54	integral membrane protein (*T. verrucosum*)
TERG_05251	1.54	phosphoglycerate kinase
TERG_03912	1.52	hypothetical protein
TERG_00765	1.52	hypothetical protein
TERG_12515	1.51	DNA repair protein Dds20/Mei5, putative (*A. benhamiae*)
TERG_08877	-1.51	hypothetical protein
TERG_08003	-1.52	fungal specific transcription factor domain-containing protein (*T. equinum*)
TERG_01555	-1.53	L-serine dehydratase, putative (*T. verrucosum*)
TERG_12281	-1.53	glycosyl hydrolase (*T. tonsurans*)
TERG_11699	-1.54	hypothetical protein
TERG_11725	-1.55	C6 transcription factor, putative (*T. verrucosum*)
TERG_07958	-1.55	myosin class II heavy chain (MHC), putative (*T. verrucosum*)
TERG_04234	-1.56	hydrophobin, putative (*T. verrucosum*)
TERG_00855	-1.57	aromatic amino acid aminotransferase, putative (*A. benhamiae*)
TERG_06331	-1.57	increased rDNA silencing protein IRS4 (*T. equinum*)
TERG_04771	-1.57	ribonuclease T2 family, putative (*A. benhamiae*)
TERG_03359	-1.58	hypothetical protein
TERG_00315	-1.60	RAN protein kinase
TERG_01262	-1.61	hypothetical protein
TERG_05270	-1.62	C6 and C2H2 transcription factor RegA-like, putative (*A. benhamiae*)
TERG_03353	-1.62	endoglucanase (*T. equinum*)

TERG_04042	-1.63	serine/threonine protein kinase
TERG_02287	-1.65	calcium dependent mitochondrial carrier protein (*T. tonsurans*)
TERG_01841	-1.65	hypothetical protein
TERG_12232	-1.66	DUF221 domain-containing protein (*T. equinum*)
TERG_12397	-1.67	hypothetical protein
TERG_08095	-1.68	MFS multidrug transporter, putative (*A. benhamiae*)
TERG_00510	-1.70	hypothetical protein
TERG_08761	-1.71	protein bfr2
TERG_06883	-1.72	NAD dependent epimerase/dehydratase family protein (*T. verrucosum*)
TERG_11624	-1.72	hypothetical protein
TERG_02487	-1.73	NADPH oxidase isoform 2 (*T. equinum*)
TERG_12576	-1.73	jumonji family transcription factor (*T. equinum*)
TERG_04240	-1.76	cupin 2 domain-containing protein (*T. equinum*)
TERG_00442	-1.76	hypothetical protein
TERG_00829	-1.76	hypothetical protein
TERG_05651	-1.77	hypothetical protein
TERG_01726	-1.77	beta-lactamase (*T. equinum*)
TERG_05626	-1.78	chitinase (*T. equinum*)
TERG_08185	-1.78	hypothetical protein
TERG_11884	-1.79	ABC bile acid transporter, putative (*T. verrucosum*)
TERG_07723	-1.92	oxidoreductase, short chain dehydrogenase/reductase family superfamily (*T. verrucosum*)
TERG_01138	-1.94	monocarboxylate transporter 4 (*M. gypseum*)
TERG_00766	-2.00	sensor histidine kinase/response regulator, putative (*A. benhamiae*)
TERG_07665	-2.01	hypothetical protein
TERG_08780	-2.01	hypothetical protein
TERG_00520	-2.03	hypothetical protein
TERG_11710	-2.07	hypothetical protein
TERG_03955	-2.22	RNA 3'-terminal phosphate cyclase, putative (*T. verrucosum*)
TERG_07406	-2.33	alpha-mannosidase (*T. tonsurans*)
TERG_12078	-2.36	hypothetical protein
TERG_02133	-2.42	fluG protein (*T. equinum*)
TERG_11574	-2.53	hypothetical protein
TERG_07331	-2.57	integral membrane protein (*T. tonsurans*)
TERG_00088	-2.68	beta-xylosidase, putative (*A. benhamiae*)
TERG_12314	-2.70	hypothetical protein
TERG_03160	-3.31	hypothetical protein
TERG_04250	-3.76	enoyl-CoA hydratase/isomerase family protein (*A. benhamiae*)
TERG_08585	-3.90	ankyrin repeat protein (*A. benhamiae*)
TERG_11948	-3.92	hypothetical protein
TERG_08806	-4.08	hypothetical protein

Gene expression values are expressed in log_2 fold change.

ID	24 hours	48 hours	96 hours	Gene Product Name
TERG_04047		5.70		cysteine-rich secreted protein (*A. benhamiae*)
TERG_03198		5.07		hypothetical protein
TERG_11907		4.95		FAD binding domain containing protein (*T. equinum*)
TERG_05408		4.64		ThiJ/PfpI family protein (*T. tonsurans*)
TERG_08285		4.59		hypothetical protein
TERG_05144		3.73		hypothetical protein
TERG_02715		3.17		beta-lactamase (*T. equinum*)
TERG_07210		3.16		ankyrin repeat protein (*T. equinum*)
TERG_12644		3.12		MFS transporter, putative (*A. benhamiae*)
TERG_08643		3.04		hypothetical protein
TERG_07409		2.96		amino acid permease (*T. equinum*)
TERG_08666		2.84		hypothetical protein
TERG_06784		2.70		transcriptional activator of ethanol catabolism AlcS (*A. benhamiae*)
TERG_01623		2.67		MFS transporter (*T. equinum*)
TERG_08722		2.57		hypothetical protein
TERG_00029		2.55		copper fist DNA binding domain-containing protein (*T. equinum*)
TERG_03893		2.50		C6 transcription factor, putative (*A. benhamiae*)
TERG_01930		2.42		hypothetical protein
TERG_06291		2.40		malate dehydrogenase (*T. verrucosum*)
TERG_03157		2.40		NDT80 / PhoG like DNA-binding family protein (*A. benhamiae*)
TERG_03130		2.32		C2H2 transcription factor (Rpn4), putative (*T. verrucosum*)
TERG_00951		2.25		hypothetical protein
TERG_04732		2.24		hypothetical protein
TERG_12447		2.23		F-box domain-containing protein (*M. gypseum*)
TERG_04791		2.22		hypothetical protein
TERG_02092		2.20		hypothetical protein
TERG_00161		2.19		hypothetical protein
TERG_11767		2.14		amidase family protein, putative (*T. verrucosum*)
TERG_05564		2.11		hypothetical protein
TERG_08262		2.09		C6 transcription factor, putative (*A. benhamiae*)
TERG_08074		2.09		hypothetical protein
TERG_00351		2.08		eukaryotic translation initiation factor eIF-2C4 (*T. tonsurans*)
TERG_11853		2.07		hypothetical protein
TERG_07031		2.06		hypothetical protein
TERG_07594		2.05		cytidylyltransferase (*T. tonsurans*)
TERG_02349		2.05		hypothetical protein
TERG_02122		2.04		adenylate-forming enzyme, putative (*A. benhamiae*)
TERG_11705		2.03		hypothetical protein
TERG_07410		2.02		hypothetical protein
TERG_11582		2.01		hypothetical protein
TERG_02569		1.99		conserved serine proline-rich protein (*A. benhamiae*)

TERG_12462	1.98	hydroxymethylglutaryl-CoA reductase (NADPH)
TERG_06515	1.93	tetracycline transporter (*T. equinum*)
TERG_02076	1.91	hypothetical protein
TERG_12252	1.90	GTP-binding protein (*T. tonsurans*)
TERG_01212	1.89	mRNA binding protein Pumilio 2, putative (*T. verrucosum*)
TERG_11655	1.88	hypothetical protein
TERG_02065	1.88	tetratricopeptide repeat protein (*T. verrucosum*)
TERG_01645	1.86	mitochondrial carrier protein (*T. tonsurans*)
TERG_01510	1.84	proline-specific permease (*T. tonsurans*)
TERG_04875	1.84	monocarboxylate permease (*T. equinum*)
TERG_01725	1.84	galactonate dehydratase (*T. equinum*)
TERG_08990	1.83	hypothetical protein
TERG_11586	1.83	phosphoketolase (*T. tonsurans*)
TERG_08191	1.82	glucooligosaccharide oxidase (*T. equinum*)
TERG_02714	1.81	proteinase, putative (*A. benhamiae*)
TERG_02066	1.81	MGS207 protein (*M. gypseum*)
TERG_00754	1.80	hypothetical protein
TERG_07317	1.80	phosphatidylinositol phospholipase C (*T. tonsurans*)
TERG_07252	1.78	hypothetical protein
TERG_03364	1.78	hypothetical protein
TERG_00912	1.78	hypothetical protein
TERG_01761	1.78	hypothetical protein
TERG_02469	1.77	hypothetical protein
TERG_04901	1.77	TBC1 domain family member 20 (*T. equinum*)
TERG_08049	1.75	cation diffusion facilitator, putative (*T. verrucosum*)
TERG_03695	1.74	pyrroline-5-carboxylate reductase
TERG_07184	1.73	HMG box transcriptional regulator, putative (*A. benhamiae*)
TERG_00895	1.73	hypothetical protein
TERG_01223	1.73	F-box domain protein (*T. verrucosum*)
TERG_12434	1.73	hypothetical protein
TERG_07761	1.72	hypothetical protein
TERG_01916	1.72	TdiA protein (*T. equinum*)
TERG_12531	1.72	hypothetical protein
TERG_08821	1.71	ribosome biogenesis (*T. equinum*)
TERG_11801	1.71	serine/threonine protein kinase (*T. equinum*)
TERG_02867	1.70	ribosome biogenesis ATPase RIX7 (*T. tonsurans*)
TERG_11800	1.69	serine/threonine protein kinase (*T. equinum*)
TERG_02405	1.68	mating locus protein, putative (*T. verrucosum*)
TERG_01394	1.68	cytochrome P450 alkane hydroxylase, putative (*T. verrucosum*)
TERG_02820	1.68	polyketide synthase (*T. tonsurans*)
TERG_00585	1.67	indoleamine 2,3-dioxygenase pyrrole 2,3-dioxygenase (*A. benhamiae*)
TERG_04778	1.67	ribosome assembly and transport protein Srp40, putative (*T. verrucosum*)
TERG_11516	1.66	hypothetical protein

TERG_12533	1.66	hypothetical protein
TERG_04206	1.66	AT DNA binding protein (*T. equinum*)
TERG_07436	1.66	ATP-dependent RNA helicase mss116 (*T. equinum*)
TERG_05142	1.66	dimeric dihydrodiol dehydrogenase, putative (*T. verrucosum*)
TERG_08216	1.65	ATP-dependent RNA helicase dbp6 (*T. equinum*)
TERG_07577	1.64	KOW motif domain protein (*T. verrucosum*)
TERG_07222	1.64	carbonic anhydrase family protein (*T. verrucosum*)
TERG_03026	1.64	WD repeat protein (*T. tonsurans*)
TERG_08047	1.63	hypothetical protein
TERG_05023	1.62	calcium/proton exchanger
TERG_01907	1.62	hypothetical protein
TERG_04949	1.61	siderophore iron transporter mirC
TERG_07760	1.61	integral membrane protein (*T. verrucosum*)
TERG_04731	1.61	STE/STE20 protein kinase (*T. equinum*)
TERG_04359	1.61	hypothetical protein
TERG_11757	1.61	hypothetical protein
TERG_11694	1.61	hypothetical protein
TERG_00192	1.61	ATP-dependent rRNA helicase spb4 (*T. tonsurans*)
TERG_00253	1.60	HNRNP arginine N-methyltransferase (*T. tonsurans*)
TERG_01368	1.59	Ribosomal protein L7Ae/L30e/S12e/Gadd45 family protein (*T. equinum*)
TERG_08298	1.59	RTA1 domain-containing protein (*T. equinum*)
TERG_01210	1.59	hypothetical protein
TERG_05724	1.59	hypothetical protein
TERG_04493	1.58	meiotic PUF family protein 1 (*A. benhamiae*)
TERG_08342	1.58	CMGC/SRPK protein kinase (*T. tonsurans*)
TERG_05384	1.58	hypothetical protein
TERG_05557	1.58	cobW domain-containing protein (*T. equinum*)
TERG_01811	1.58	triacylglycerol lipase, putative (*A. benhamiae*)
TERG_02562	1.58	chitin synthase C
TERG_01968	1.57	RNA binding protein, putative (*T. verrucosum*)
TERG_03704	1.56	integral membrane protein Pth11-like, putative (*T. verrucosum*)
TERG_08333	1.56	1-pyrroline-5-carboxylate dehydrogenase
TERG_12058	1.55	transporter smf2 (*T. tonsurans*)
TERG_05435	1.55	hypothetical protein
TERG_08142	1.54	tRNA-dihydrouridine synthase (*T. equinum*)
TERG_04309	1.54	multidrug resistance protein (*T. equinum*)
TERG_12292	1.53	hypothetical protein
TERG_02406	1.53	alpha-box mating type protein (*T. tonsurans*)
TERG_00159	1.53	hypothetical protein
TERG_08004	1.53	hypothetical protein
TERG_05388	1.52	ribosome biogenesis protein (*T. tonsurans*)
TERG_07408	1.52	acetyltransferase, GNAT family (*A. benhamiae*)
TERG_02257	1.52	DNA-directed RNA polymerase

TERG_12461	1.52	HMG-CoA reductase (*A. benhamiae*)
TERG_05401	1.52	rRNA processing protein Bystin (*T. tonsurans*)
TERG_00631	1.52	urate oxidase
TERG_12408	1.52	purine nucleoside phosphorylase I (*T. tonsurans*)
TERG_02672	1.52	ATP-dependent RNA helicase has1
TERG_06328	1.51	AdoMet-dependent rRNA methyltransferase spb1
TERG_12251	1.51	GTP-binding protein (*T. tonsurans*)
TERG_06433	1.51	mitochondrial folate carrier protein (*T. tonsurans*)
TERG_03793	1.51	elongation factor 3 (*M. gypseum*)
TERG_00196	1.51	hypothetical protein
TERG_00508	-1.50	hypothetical protein
TERG_00227	-1.51	glutamine amidotransferase subunit pdxT (*T. equinum*)
TERG_01754	-1.51	oxidoreductase, 2-nitropropane dioxygenase family, putative (*A. benhamiae*)
TERG_02131	-1.51	hypothetical protein
TERG_08961	-1.52	hypothetical protein
TERG_01052	-1.52	coenzyme A transferase, putative (*A. benhamiae*)
TERG_03753	-1.53	hypothetical protein
TERG_07678	-1.53	endoribonuclease L-PSP (*T. tonsurans*)
TERG_01143	-1.53	CMGC protein kinase (*T. tonsurans*)
TERG_08854	-1.53	mitochondrial hypoxia responsive domain-containing protein (*T. tonsurans*)
TERG_07334	-1.54	HIT domain-containing protein (*T. equinum*)
TERG_08057	-1.55	hypothetical protein
TERG_02767	-1.55	pyroglutamyl peptidase type I (*T. equinum*)
TERG_07287	-1.55	hypothetical protein
TERG_01172	-1.56	hypothetical protein
TERG_05579	-1.56	FAD dependent oxidoreductase, putative (*A. benhamiae*)
TERG_00642	-1.56	HHE domain-containing protein (*T. equinum*)
TERG_01079	-1.56	prenyltransferase (*T. equinum*)
TERG_00197	-1.57	aldose 1-epimerase family protein, putative (*T. verrucosum*)
TERG_00204	-1.59	mannitol-1-phosphate 5-dehydrogenase
TERG_05363	-1.59	hypothetical protein
TERG_07089	-1.59	GPI-anchored wall transfer protein 1 (*T. tonsurans*)
TERG_06521	-1.60	hypothetical protein
TERG_01509	-1.62	hypothetical protein
TERG_05643	-1.64	hypothetical protein
TERG_06838	-1.64	oxalate/formate antiporter, putative (*T. verrucosum*)
TERG_04521	-1.65	HypA-like protein, putative (*A. benhamiae*)
TERG_02041	-1.65	glutathione S-transferase (*T. equinum*)
TERG_03780	-1.67	pyruvate dehydrogenase dihydrolipoamide acetyltransferase component (*M. gypseum*)
TERG_08240	-1.68	methyltransferase, putative (*T. verrucosum*)
TERG_07291	-1.68	hypothetical protein
TERG_03154	-1.68	2-nitropropane dioxygenase (*M. gypseum*)
TERG_06178	-1.69	calpain-like protein (*T. verrucosum*)

TERG_12323	-1.70	hypothetical protein
TERG_06402	-1.73	Ser/Thr protein phosphatase family protein (*T. verrucosum*)
TERG_00563	-1.73	GNAT family N-acetyltransferase, putative (*A. benhamiae*)
TERG_05339	-1.73	endonuclease/exonuclease/phosphatase (*T. equinum*)
TERG_07139	-1.74	hypothetical protein
TERG_11792	-1.75	hypothetical protein
TERG_06908	-1.77	raffinose synthase protein Sip1, putative (*T. verrucosum*)
TERG_07876	-1.77	hypothetical protein
TERG_05601	-1.77	MFS transporter (*T. equinum*)
TERG_07964	-1.78	vacuolar endopolyphosphatase, putative (*T. verrucosum*)
TERG_05452	-1.78	GABA permease (*T. tonsurans*)
TERG_05290	-1.80	C6 finger domain-containing protein (*T. tonsurans*)
TERG_02851	-1.83	hypothetical protein
TERG_02546	-1.85	acetyltransferase (*T. tonsurans*)
TERG_06594	-1.86	DUF1183 domain-containing protein (*T. equinum*)
TERG_03218	-1.86	extracellular salicylate hydroxylase/monooxygenase, putative (*T. verrucosum*)
TERG_12188	-1.86	aldo/keto reductase/Endoribonuclease L-PSP (*M. canis*)
TERG_01895	-1.86	pheromone processing carboxypeptidase (Sxa2), putative (*T. verrucosum*)
TERG_05201	-1.86	integral membrane protein (*T. equinum*)
TERG_06332	-1.86	AGC/AKT protein kinase
TERG_11986	-1.86	GABA permease, putative (*A. benhamiae*)
TERG_12721	-1.87	transferase (*M. canis*)
TERG_00538	-1.87	oxidoreductase (*T. tonsurans*)
TERG_01177	-1.90	cytochrome P450 51 (*T. equinum*)
TERG_12032	-1.91	hypothetical protein
TERG_05650	-1.91	blasticidin-resistance protein (*T. tonsurans*)
TERG_07983	-1.93	hypothetical protein
TERG_07280	-1.94	hypothetical protein
TERG_03422	-1.96	hypothetical protein
TERG_04741	-1.98	galactose-1-phosphate uridylyltransferase
TERG_02242	-2.01	yeast phase specific protein (*M. gypseum*)
TERG_05486	-2.03	Atg28p (*T. equinum*)
TERG_01190	-2.04	RNA binding protein (*T. equinum*)
TERG_12598	-2.08	hypothetical protein
TERG_03533	-2.13	DRAP deaminase, putative (*A. benhamiae*)
TERG_00097	-2.14	hypothetical protein
TERG_06062	-2.15	hypothetical protein
TERG_02610	-2.17	WD repeat protein (*T. verrucosum*)
TERG_05876	-2.21	hypothetical protein
TERG_07743	-2.25	hypothetical protein
TERG_12715	-2.25	integral membrane protein (*M. canis*)
TERG_05409	-2.29	FAD dependent oxidoreductase (*T. equinum*)

TERG_00540	-2.35	oxidoreductase, zinc-binding, putative (*T. verrucosum*)
TERG_06205	-2.41	integral membrane protein (*T. tonsurans*)
TERG_07310	-2.42	hypothetical protein
TERG_07734	-2.53	O-methyltransferase, putative (*T. verrucosum*)
TERG_03848	-2.69	hypothetical protein
TERG_08756	-2.70	cercosporin toxin biosynthesis protein (*T. equinum*)
TERG_01741	-2.72	hypothetical protein
TERG_05032	-2.90	C6 transcription factor (*T. equinum*)
TERG_06312	-2.94	hypothetical protein
TERG_06466	-3.02	hypothetical protein
TERG_08744	-3.14	hypothetical protein
TERG_01280	-3.25	IgE-binding protein (*A. benhamiae*)
TERG_01995	-3.37	hypothetical protein
TERG_00026	-5.05	flavin-binding monooxygenase, putative (*T. verrucosum*)

Gene expression values are expressed in log_2 fold change.

ID	24 hours	48 hours	96 hours	Gene Product Name
TERG_01659			5.83	heat shock protein Hsp30-like, putative (*A. benhamiae*)
TERG_12507			5.14	hsp98-like protein (*T. equinum*)
TERG_07949			4.69	heat shock protein 78 (*T. tonsurans*)
TERG_01731			4.27	hypothetical protein
TERG_04087			4.12	DnaJ domain-containing protein (*T. tonsurans*)
TERG_07658			4.07	hsp88-like protein (*T. tonsurans*)
TERG_06505			3.99	hsp70-like protein
TERG_01661			3.82	fumarylacetoacetate hydrolase domain-containing protein 2A (*T. equinum*)
TERG_06963			3.72	hsp90-like protein
TERG_05922			3.61	acyl-CoA desaturase
TERG_04141			3.42	hsp60-like protein
TERG_04032			3.39	DNA repair and transcription factor Ada, putative (*A. benhamiae*)
TERG_06634			3.37	hsp70-like protein (*T. equinum*)
TERG_02590			3.37	peptidyl-prolyl cis-trans isomerase D
TERG_03677			3.19	protein phosphatase regulatory subunit Gac1, putative (*T. verrucosum*)
TERG_05655			3.19	AN1 zinc finger protein (*T. equinum*)
TERG_00669			3.11	C-5 sterol desaturase (*T. tonsurans*)
TERG_01683			3.05	hypothetical protein
TERG_12448			3.02	hypothetical protein
TERG_07959			3.01	GPI-anchored cell wall beta-1,3-endoglucanase EglC (*A. benhamiae*)
TERG_03105			3.00	hypothetical protein
TERG_03102			3.00	sterol 24-C-methyltransferase (*T. equinum*)
TERG_01893			2.92	hypothetical protein
TERG_12618			2.91	hypothetical protein

TERG_07570	2.89	G-protein signaling regulator, putative (*T. verrucosum*)
TERG_06756	2.88	DnaJ domain protein (*A. benhamiae*)
TERG_06104	2.85	chaperone DnaJ
TERG_08533	2.83	poly(A) polymerase Cid1 (*T. equinum*)
TERG_00243	2.80	pathogenesis associated protein Cap20, putative (*T. verrucosum*)
TERG_06496	2.77	coproporphyrinogen III oxidase (*T. tonsurans*)
TERG_08528	2.73	fatty acid synthase beta subunit dehydratase (*T. tonsurans*)
TERG_12519	2.72	hypothetical protein
TERG_02166	2.72	oxidoreductase (*T. equinum*)
TERG_05717	2.71	squalene epoxidase (*T. equinum*)
TERG_01843	2.70	inositol-3-phosphate synthase (*T. tonsurans*)
TERG_12038	2.70	protein phosphatase regulatory subunit Gac1, putative (*T. verrucosum*)
TERG_08312	2.68	mitochondrial heat shock protein Hsp10 (*A. benhamiae*)
TERG_03506	2.65	hypothetical protein
TERG_04956	2.65	hypothetical protein
TERG_00668	2.64	L-galactose dehydrogenase (*T. tonsurans*)
TERG_06398	2.61	Hsp90 co-chaperone Cdc37 (*T. tonsurans*)
TERG_12619	2.59	hypothetical protein
TERG_12701	2.58	fatty acid synthase subunit alpha
TERG_07274	2.57	hypothetical protein
TERG_00339	2.56	hydroxymethylglutaryl-CoA synthase
TERG_02186	2.55	ABC multidrug transporter, putative (*A. benhamiae*)
TERG_08090	2.51	plasma membrane iron permease (*T. tonsurans*)
TERG_12700	2.42	fatty acid synthase subunit alpha
TERG_03065	2.37	NADP-dependent malic enzyme MaeA (*T. tonsurans*)
TERG_01609	2.36	Na/K ATPase alpha 1 subunit (*T. equinum*)
TERG_01364	2.36	hypothetical protein
TERG_07477	2.35	copper-sulfate regulated protein 1 (*T. equinum*)
TERG_01379	2.34	serine/threonine-protein phosphatase 2A activator 2 (*T. equinum*)
TERG_00066	2.34	4-hydroxyphenylpyruvate dioxygenase
TERG_08845	2.31	pyridoxamine phosphate oxidase (*T. tonsurans*)
TERG_03380	2.29	FK506-binding protein 1A (*T. equinum*)
TERG_02809	2.24	hypothetical protein
TERG_11969	2.23	NAD-specific glutamate dehydrogenase (*M. gypseum*)
TERG_05575	2.20	MFS multidrug transporter (*T. tonsurans*)
TERG_06693	2.19	integral membrane protein (*T. equinum*)
TERG_00890	2.17	cell surface protein, putative (*T. verrucosum*)
TERG_04964	2.16	RNA-binding protein (*A. benhamiae*)
TERG_07652	2.16	Delta(12) fatty acid desaturase (*T. equinum*)
TERG_08228	2.13	sphinganine hydroxylase (*T. tonsurans*)
TERG_03384	2.13	aspartate aminotransferase, putative (*A. benhamiae*)
TERG_00574	2.12	MFS multidrug transporter, putative (*T. verrucosum*)

TERG_05021	2.12	C6 transcription factor, putative (*A. benhamiae*)
TERG_07083	2.10	hypothetical protein
TERG_01411	2.09	Hsp90 binding co-chaperone (Sba1), putative (*T. verrucosum*)
TERG_04382	2.08	c-14 sterol reductase (*T. tonsurans*)
TERG_06805	2.08	bax Inhibitor family protein (*T. equinum*)
TERG_05810	2.07	MFS maltose permease, putative (*T. verrucosum*)
TERG_11621	2.06	hypothetical protein
TERG_06504	2.06	hypothetical protein
TERG_06167	2.03	hypothetical protein
TERG_04688	2.03	salicylate hydroxylase, putative (*T. verrucosum*)
TERG_06399	2.02	ATP-dependent permease MDL2 (*T. equinum*)
TERG_06070	2.01	polysaccharide synthase Cps1 (*T. tonsurans*)
TERG_03052	1.99	glycosyl hydrolase (*T. tonsurans*)
TERG_01419	1.99	protein pyrABCN
TERG_12107	1.98	1,4-alpha-glucan-branching enzyme
TERG_07552	1.97	hypothetical protein
TERG_06869	1.97	DNA-directed RNA polymerase III subunit RPC6 (*T. equinum*)
TERG_07320	1.97	hypothetical protein
TERG_11968	1.96	hypothetical protein
TERG_06308	1.96	XPG I-region protein (*M. gypseum*)
TERG_05172	1.96	pyridine nucleotide-disulphide oxidoreductase family protein (*A. benhamiae*)
TERG_08405	1.95	leucine aminopeptidase 2
TERG_07138	1.94	polyubiquitin
TERG_06938	1.94	zinc metallopeptidase (*T. tonsurans*)
TERG_12610	1.93	CCAAT-box-binding transcription factor (*T. equinum*)
TERG_03054	1.93	glycosyl hydrolase (*T. tonsurans*)
TERG_02529	1.93	hypothetical protein
TERG_03514	1.92	mitochondrial co-chaperone GrpE (*T. tonsurans*)
TERG_04043	1.91	hypothetical protein
TERG_03775	1.91	fructose-2,6-bisphosphatase (*T. tonsurans*)
TERG_00302	1.91	GTP-binding protein rho2
TERG_08139	1.90	NAD dependent epimerase/dehydratase (*M. gypseum*)
TERG_07950	1.90	translation elongation factor 1 subunit Eef1-beta (*T. equinum*)
TERG_01787	1.90	integral membrane protein (*T. equinum*)
TERG_03414	1.90	acetyl-CoA carboxylase (*T. equinum*)
TERG_00067	1.90	hypothetical protein
TERG_03654	1.90	NADH-cytochrome b5 reductase 1
TERG_06475	1.90	zinc knuckle domain-containing protein (*T. tonsurans*)
TERG_11740	1.89	hypothetical protein
TERG_07639	1.89	atypical/ABC1/ABC1-B protein kinase (*T. tonsurans*)
TERG_04153	1.88	60S ribosome biogenesis protein Brx1 (*T. tonsurans*)
TERG_08091	1.88	conidial pigment biosynthesis oxidase Abr1/brown (*T. verrucosum*)
TERG_01785	1.87	HIT finger domain-containing protein (*T. equinum*)

TERG_05155	1.86	Leucine carboxyl methyltransferase family (*A. benhamiae*)
TERG_12108	1.86	1,4-alpha-glucan-branching enzyme
TERG_00881	1.85	zinc-containing alcohol dehydrogenase, putative (*T. verrucosum*)
TERG_03336	1.83	Sec14 cytosolic factor (*T. equinum*)
TERG_02327	1.82	rRNA biogenesis protein RRP5 (*T. tonsurans*)
TERG_02528	1.82	ubiE/COQ5 methyltransferase, putative (*T. verrucosum*)
TERG_07720	1.82	copper-transporting ATPase (*T. equinum*)
TERG_00437	1.81	ribosome biogenesis protein Kri1 (*T. equinum*)
TERG_01881	1.80	hypothetical protein
TERG_01612	1.80	enolase
TERG_07444	1.79	nuclear segregation protein (*T. tonsurans*)
TERG_11642	1.78	forkhead transcription factor (*T. tonsurans*)
TERG_01883	1.77	hsp75-like protein
TERG_02230	1.76	mitochondrial ribosomal protein DAP3, putative (*A. benhamiae*)
TERG_05367	1.75	nucleolar complex-associated protein 3 (*T. equinum*)
TERG_05194	1.75	elongation factor Tu, mitochondrial
TERG_05956	1.75	rRNA processing protein Ebp2p (*T. equinum*)
TERG_02979	1.75	Delta(24(24(1)))-sterol reductase (*T. tonsurans*)
TERG_12345	1.74	kinesin family protein (*T. tonsurans*)
TERG_00231	1.74	glutamate decarboxylase (*T. equinum*)
TERG_07295	1.74	C6 finger domain protein, putative (*T. verrucosum*)
TERG_06621	1.73	60S ribosomal subunit assembly/export protein loc1 (*T. tonsurans*)
TERG_00285	1.72	hypothetical protein
TERG_07053	1.71	hypothetical protein
TERG_05811	1.71	40S ribosomal protein S8 (*T. tonsurans*)
TERG_01523	1.70	hypothetical protein
TERG_11940	1.70	hypothetical protein
TERG_00359	1.68	MeaB protein (*T. equinum*)
TERG_05110	1.68	37S ribosomal protein S5 (*T. tonsurans*)
TERG_05739	1.68	mitochondrial exoribonuclease Cyt-4, putative (*T. verrucosum*)
TERG_07337	1.67	54S ribosomal protein RML2 (*T. equinum*)
TERG_02231	1.67	ribosome biogenesis protein (*T. tonsurans*)
TERG_08483	1.67	hypothetical protein
TERG_02385	1.67	hypothetical protein
TERG_00677	1.67	hypothetical protein
TERG_00976	1.67	serine hydroxymethyltransferase, cytosolic
TERG_12344	1.67	kinesin family protein (*T. tonsurans*)
TERG_04998	1.65	mitochondrial ATP-dependent RNA helicase (*T. tonsurans*)
TERG_01619	1.65	toxin biosynthesis protein (Tri7), putative (*T. verrucosum*)
TERG_08590	1.65	tRNA (adenine-N(1)-)-methyltransferase catalytic subunit trm61 (*T. tonsurans*)
TERG_03569	1.63	RING finger domain-containing protein (*T. equinum*)

TERG_12045	1.63	hypothetical protein
TERG_08991	1.63	ATPase 2 nuclear control (*M. gypseum*)
TERG_01206	1.63	pre-mRNA-splicing factor ATP-dependent RNA helicase PRP43 (*T. equinum*)
TERG_04173	1.63	hypothetical protein
TERG_05942	1.62	integral membrane protein (*T. equinum*)
TERG_00213	1.62	hypothetical protein
TERG_08122	1.62	hypothetical protein
TERG_06619	1.62	DNA-directed RNA polymerase I and III polypeptide (*T. equinum*)
TERG_06390	1.61	sulfite reductase flavoprotein alpha-component (*T. equinum*)
TERG_12054	1.61	HEAT repeat protein (*A. benhamiae*)
TERG_05307	1.61	hypothetical protein
TERG_05734	1.61	ubiquitin fusion degradation protein (*T. tonsurans*)
TERG_04058	1.60	STE/STE11 protein kinase
TERG_06516	1.60	hypothetical protein
TERG_04767	1.60	30S ribosomal protein S7 (*T. tonsurans*)
TERG_00784	1.59	succinate dehydrogenase assembly factor 2, mitochondrial
TERG_00705	1.59	Ras family protein (*T. tonsurans*)
TERG_06032	1.59	ribosomal small subunit assembly protein, putative (*A. benhamiae*)
TERG_03139	1.59	hypothetical protein
TERG_00684	1.59	ubiquinol-cytochrome C chaperone (*T. equinum*)
TERG_08511	1.58	hypothetical protein
TERG_06967	1.58	rnapii degradation factor def1 (*T. tonsurans*)
TERG_00123	1.58	ribonuclease P complex subunit Pop1 (*T. tonsurans*)
TERG_06739	1.58	hypothetical protein
TERG_07125	1.58	peptidyl-tRNA hydrolase
TERG_06578	1.57	glutathione S-transferase, putative (*A. benhamiae*)
TERG_08655	1.57	ADP,ATP carrier protein
TERG_00931	1.56	hypothetical protein
TERG_00456	1.56	cysteine dioxygenase, putative (*A. benhamiae*)
TERG_05786	1.55	hypothetical protein
TERG_06907	1.55	tyrosine-tRNA ligase
TERG_06641	1.54	mitochondrial mRNA processing protein PET127 (*T. tonsurans*)
TERG_08038	1.54	polysaccharide deacetylase family protein (*T. equinum*)
TERG_00455	1.54	ribosome-interacting GTPase 1
TERG_00249	1.53	ATP-dependent rRNA helicase RRP3
TERG_00810	1.52	37S ribosomal protein Rsm24 (*T. equinum*)
TERG_01447	1.52	bcp1 (*T. equinum*)
TERG_02420	1.52	hypothetical protein
TERG_06557	1.52	ribosomal protein L7/L12
TERG_08212	1.51	NOL1/NOP2/sun domain protein, putative (*T. verrucosum*)
TERG_07111	1.51	tRNA-dihydrouridine synthase 3
TERG_03175	1.51	DUF833 domain protein (*A. benhamiae*)

TERG_00241	1.51	hypothetical protein
TERG_08540	1.51	rRNA-processing protein FCF1 (*T. equinum*)
TERG_06577	1.51	37S ribosomal protein S9 (*T. verrucosum*)
TERG_08258	1.50	hypothetical protein
TERG_02363	-1.50	succinate-semialdehyde dehydrogenase (*T. tonsurans*)
TERG_07236	-1.50	hypothetical protein
TERG_04100	-1.51	dolichyl-phosphate beta-glucosyltransferase (*T. tonsurans*)
TERG_03772	-1.51	hypothetical protein
TERG_02112	-1.51	hypothetical protein
TERG_07285	-1.51	hypothetical protein
TERG_06378	-1.51	hypothetical protein
TERG_02849	-1.51	flavin-containing monooxygenase (*T. equinum*)
TERG_05617	-1.52	hypothetical protein
TERG_02001	-1.52	oligopeptidase (*T. tonsurans*)
TERG_04763	-1.52	fructosyl amine:oxygen oxidoreductase (*T. tonsurans*)
TERG_05841	-1.53	AmmeMemoRadiSam system protein B
TERG_00969	-1.53	hypothetical protein
TERG_01559	-1.53	amino acid transporter, putative (*A. benhamiae*)
TERG_06766	-1.53	high affinity methionine permease (*T. tonsurans*)
TERG_08048	-1.53	hypothetical protein
TERG_01511	-1.53	hypothetical protein
TERG_07158	-1.54	cell cycle regulatory protein, putative (*T. verrucosum*)
TERG_07888	-1.54	acyl CoA binding protein (*T. tonsurans*)
TERG_01404	-1.54	aldehyde dehydrogenase
TERG_01270	-1.54	AMP dependent ligase (*T. tonsurans*)
TERG_04341	-1.55	C6 transcription factor (*T. equinum*)
TERG_06131	-1.55	hypothetical protein
TERG_01943	-1.56	1-aminocyclopropane-1-carboxylate deaminase
TERG_05309	-1.56	major facilitator superfamily protein (*T. equinum*)
TERG_06879	-1.57	acetamidase (*T. verrucosum*)
TERG_00553	-1.57	amino acid permease (*T. equinum*)
TERG_00845	-1.57	aldolase (*T. tonsurans*)
TERG_05465	-1.57	GTP-binding protein yptV5 (*T. tonsurans*)
TERG_00292	-1.57	hypothetical protein
TERG_12659	-1.58	transferase family protein (*A. benhamiae*)
TERG_02038	-1.58	peroxisomal targeting signal receptor (*T. equinum*)
TERG_08518	-1.58	DUF614 domain protein (*A. benhamiae*)
TERG_08130	-1.58	ABC ATPase (*T. equinum*)
TERG_01851	-1.59	Pfs, NACHT and WD domain protein (*A. benhamiae*)
TERG_07838	-1.59	YjeF family domain-containing protein
TERG_00844	-1.60	hypothetical protein
TERG_06732	-1.60	peroxisomal membrane protein receptor Pex19, putative (*A. benhamiae*)
TERG_12436	-1.60	monocarboxylate transporter (*M. canis*)

TERG_03790	-1.60	DUF1275 domain-containing protein (*T. equinum*)
TERG_08103	-1.61	anaphase promoting complex subunit 10 (APC10), putative (*A. benhamiae*)
TERG_05500	-1.62	pantetheine-phosphate adenylyltransferase family protein (*T. verrucosum*)
TERG_07816	-1.62	nitrate reductase (*T. tonsurans*)
TERG_01164	-1.62	beta-alanine synthase, putative (*T. verrucosum*)
TERG_05580	-1.62	hypothetical protein
TERG_05173	-1.63	hypothetical protein
TERG_05405	-1.64	methionine permease (*T. equinum*)
TERG_07715	-1.64	hypothetical protein
TERG_11942	-1.64	MFS maltose permease (*T. tonsurans*)
TERG_00340	-1.65	hypothetical protein
TERG_08264	-1.66	L-xylulose reductase
TERG_08055	-1.66	ToxD-like zinc binding oxidoreductase, putative (*A. benhamiae*)
TERG_03032	-1.66	autophagy protein Atg13, putative (*A. benhamiae*)
TERG_06888	-1.67	transulfuration enzyme family protein, putative (*A. benhamiae*)
TERG_00487	-1.67	hypothetical protein
TERG_00246	-1.67	formyl-coenzyme A transferase (*T. equinum*)
TERG_08042	-1.68	hypothetical protein
TERG_08252	-1.68	LysM domain protein, putative (*A. benhamiae*)
TERG_05156	-1.69	Pps1 dual specificity phosphatase (*T. tonsurans*)
TERG_06155	-1.69	phosphotransferase (*T. tonsurans*)
TERG_05299	-1.69	glutathione S-transferase, putative (*A. benhamiae*)
TERG_05624	-1.70	C6 transcription factor, putative (*T. verrucosum*)
TERG_03461	-1.70	voltage-gated chloride channel (*T. tonsurans*)
TERG_02064	-1.70	peroxisomal biogenesis factor 6 (*T. tonsurans*)
TERG_07102	-1.71	mitochondrial distribution and morphology protein 12
TERG_11656	-1.71	class V chitinase (*T. tonsurans*)
TERG_04134	-1.72	hypothetical protein
TERG_01732	-1.74	amidohydrolase (*T. equinum*)
TERG_00032	-1.74	mitochondrial dicarboxylate transporter (*T. tonsurans*)
TERG_05205	-1.75	hypothetical protein
TERG_04807	-1.75	aminotransferase (*T. tonsurans*)
TERG_02423	-1.75	hypothetical protein
TERG_07282	-1.75	cytochrome P450 monooxygenase, putative (*T. verrucosum*)
TERG_01171	-1.76	autophagy regulatory protein Atg2, putative (*T. verrucosum*)
TERG_00701	-1.76	cytochrome b5, putative (*T. verrucosum*)
TERG_06240	-1.76	peroxisomal biogenesis factor 2 (*T. tonsurans*)
TERG_12373	-1.77	multidrug resistance protein (*T. tonsurans*)
TERG_06806	-1.77	hypothetical protein
TERG_05918	-1.78	glutamyl-tRNA(Gln) amidotransferase subunit A (*T. equinum*)
TERG_00989	-1.79	prenylcysteine lyase (*T. tonsurans*)
TERG_02197	-1.79	nitrilase, putative (*T. verrucosum*)
TERG_00878	-1.80	hypothetical protein

TERG_07750	-1.80	hypothetical protein
TERG_08235	-1.80	long chain fatty alcohol oxidase (*T. tonsurans*)
TERG_03467	-1.80	peroxisomal membrane protein Pex17 (*T. equinum*)
TERG_11702	-1.80	hypothetical protein
TERG_05837	-1.80	plasma membrane ammonium transporter (Ato3), putative (*A. benhamiae*)
TERG_05044	-1.81	epoxide hydrolase, putative (*A. benhamiae*)
TERG_08051	-1.82	3-oxoacyl-(acyl-carrier-protein) reductase (*T. tonsurans*)
TERG_01049	-1.83	hypothetical protein
TERG_05537	-1.83	MFS multidrug transporter (*T. equinum*)
TERG_02587	-1.83	hypothetical protein
TERG_01926	-1.84	5'/3'-nucleotidase SurE
TERG_08296	-1.84	fatty acid synthase beta subunit dehydratase (*M. canis*)
TERG_12586	-1.84	chromatin assembly factor 1 subunit A (*T. tonsurans*)
TERG_06997	-1.84	C6 transcription factor (*T. tonsurans*)
TERG_05967	-1.84	hypothetical protein
TERG_04620	-1.84	peroxin 20 (*T. equinum*)
TERG_05615	-1.84	hsp70-like protein (*T. equinum*)
TERG_12614	-1.85	succinyl-CoA synthetase beta subunit, putative (*T. verrucosum*)
TERG_06625	-1.86	serine protease, putative (*A. benhamiae*)
TERG_08557	-1.86	carboxypeptidase S1, putative (*A. benhamiae*)
TERG_00284	-1.86	peroxin 14 (*T. tonsurans*)
TERG_05258	-1.87	MFS transporter (*T. equinum*)
TERG_00620	-1.87	NAD+ kinase Utr1 (*T. equinum*)
TERG_03066	-1.87	MFS peptide transporter, putative (*T. verrucosum*)
TERG_02043	-1.88	cysteine desulfurase (*T. equinum*)
TERG_08868	-1.89	FKBP-type peptidyl-prolyl isomerase, putative (*A. benhamiae*)
TERG_00084	-1.91	chromosome segregation protein (Pcs1), putative (*T. verrucosum*)
TERG_08869	-1.91	hypothetical protein
TERG_03334	-1.91	sensor histidine kinase/response regulator (*T. tonsurans*)
TERG_05747	-1.93	alcohol dehydrogenase (*T. tonsurans*)
TERG_00009	-1.93	peroxisomal biogenesis factor (*T. tonsurans*)
TERG_08062	-1.96	alpha-glucosidase AgdA, putative (*A. benhamiae*)
TERG_02338	-1.97	hypothetical protein
TERG_03940	-1.97	hypothetical protein
TERG_03111	-1.98	conserved proline-rich protein (*A. benhamiae*)
TERG_00129	-1.98	hypothetical protein
TERG_01853	-1.98	hypothetical protein
TERG_06681	-2.00	glycogen debranching enzyme
TERG_07234	-2.01	hypothetical protein
TERG_02735	-2.01	fatty acid oxygenase PpoC, putative (*A. benhamiae*)
TERG_08054	-2.01	homoserine acetyltransferase (*T. tonsurans*)
TERG_07524	-2.01	hypothetical protein

TERG_04962	-2.02	hypothetical protein
TERG_03161	-2.04	hypothetical protein
TERG_05771	-2.04	hypothetical protein
TERG_01833	-2.05	peroxisomal membrane protein (*T. tonsurans*)
TERG_01281	-2.07	malate synthase, glyoxysomal
TERG_03541	-2.07	hypothetical protein
TERG_00481	-2.07	beta-glucosidase, putative (*T. verrucosum*)
TERG_03855	-2.08	WW domain-containing protein (*M. canis*)
TERG_01674	-2.08	hypothetical protein
TERG_04810	-2.09	bifunctional fatty acid transporter/acyl-CoA synthetase (FAT1), putative (*A. benhamiae*)
TERG_08437	-2.12	C2H2 transcription factor (Egr2), putative (*T. verrucosum*)
TERG_05331	-2.12	peroxisome biosynthesis protein (*T. tonsurans*)
TERG_07482	-2.12	ribonuclease H1, putative (*A. benhamiae*)
TERG_08070	-2.13	enoyl-CoA hydratase/isomerase (*T. tonsurans*)
TERG_05374	-2.14	hypothetical protein
TERG_04099	-2.14	hypothetical protein
TERG_12698	-2.16	serine/threonine protein kinase (*T. equinum*)
TERG_05608	-2.20	hypothetical protein
TERG_12681	-2.20	alpha-mannosidase (*T. tonsurans*)
TERG_00309	-2.21	esterase, putative (*A. benhamiae*)
TERG_12658	-2.22	transferase family protein (*A. benhamiae*)
TERG_08050	-2.22	acetamidase (*T. equinum*)
TERG_01637	-2.23	hypothetical protein
TERG_12600	-2.23	U3 small nucleolar ribonucleoprotein IMP4 (*T. tonsurans*)
TERG_03947	-2.24	hypothetical protein
TERG_01936	-2.25	COP9 signalosome complex subunit 1 (*T. equinum*)
TERG_02929	-2.26	carboxylesterase, putative (*T. verrucosum*)
TERG_00621	-2.26	FAD dependent oxidoreductase (*T. tonsurans*)
TERG_05780	-2.27	hypothetical protein
TERG_06053	-2.32	catalase (*T. tonsurans*)
TERG_03008	-2.35	zinc-containing alcohol dehydrogenase, putative (*A. benhamiae*)
TERG_08058	-2.37	alpha-1,2-mannosidase family protein (*T. verrucosum*)
TERG_07659	-2.38	peroxisomal 3-ketoacyl-coA thiolase (Kat1), putative (*A. benhamiae*)
TERG_00659	-2.39	3-deoxy-7-phosphoheptulonate synthase
TERG_01489	-2.39	MFS transporter (*T. tonsurans*)
TERG_11753	-2.43	MFS transporter (*T. tonsurans*)
TERG_05150	-2.46	tRNA wybutosine-synthesizing protein 2 (*T. equinum*)
TERG_04288	-2.49	hypothetical protein
TERG_00775	-2.50	hypothetical protein
TERG_01004	-2.50	extracellular sialidase/neuraminidase (*T. equinum*)
TERG_03698	-2.57	hypothetical protein
TERG_04878	-2.60	hypothetical protein
TERG_01277	-2.60	nexin-1 (*T. equinum*)

TERG_00348			-2.62	sugar transporter (*T. tonsurans*)
TERG_07451			-2.63	acetoacetate-CoA ligase
TERG_12580			-2.69	dihydrodipicolinate synthetase family protein (*T. verrucosum*)
TERG_01512			-2.74	mitochondrial tricarboxylate transporter (Ctp), putative (*T. verrucosum*)
TERG_11881			-2.86	G-patch domain-containing protein (*T. equinum*)
TERG_02335			-2.99	hypothetical protein
TERG_05948			-3.11	protein kinase subdomain-containing protein (*T. equinum*)
TERG_11677			-3.16	solute carrier family 25 member 45 (*M. gypseum*)
TERG_03252			-3.17	hypothetical protein
TERG_03895			-3.18	lipase/thioesterase family protein (*A. benhamiae*)
TERG_05890			-3.20	stress response protein Rds1 (*T. tonsurans*)
TERG_03357			-3.27	nonribosomal peptide synthetase (*T. equinum*)
TERG_02474			-3.30	hypothetical protein
TERG_05347			-3.32	epoxide hydrolase, putative (*T. verrucosum*)
TERG_05611			-5.20	hypothetical protein
TERG_07504			-5.70	sialidase (*T. tonsurans*)

Gene expression values are expressed in log_2 fold change.

**ID**	**24 hours**	**48 hours**	**96 hours**	**Gene Product Name**
TERG_01913	6.14	4.99		cytochrome P450 monooxygenase, putative (*A. benhamiae*)
TERG_07466	2.92	3.39		hypothetical protein
TERG_03312	2.92	2.75		RTA1 domain protein, putative (*A. benhamiae*)
TERG_03705	2.90	3.21		cytochrome P450 monooxygenase, putative (*A. benhamiae*)
TERG_02307	2.82	1.83		mitotic spindle checkpoint protein (Mad2B), putative (*T. verrucosum*)
TERG_03095	2.74	3.13		methyltransferase, putative (*A. benhamiae*)
TERG_06190	2.61	2.79		hypothetical protein
TERG_08360	2.57	2.09		metallo-beta-lactamase superfamily protein (*T. equinum*)
TERG_05451	2.55	2.12		integral membrane protein (*T. equinum*)
TERG_08359	2.46	1.50		FAD-dependent monooxygenase (*T. tonsurans*)
TERG_12296	2.44	1.55		hypothetical protein
TERG_03622	2.41	2.34		hypothetical protein
TERG_01994	2.39	1.75		OPT family oligopeptide transporter
TERG_11975	2.36	2.86		integral membrane protein (*T. verrucosum*)
TERG_12284	2.33	2.14		alkaline serine protease (*T. equinum*)
TERG_08190	2.32	3.50		Mrsp1 (*T. equinum*)
TERG_02298	2.32	1.63		GPI anchored cell wall protein, putative (*T. verrucosum*)
TERG_06073	2.31	2.04		MFS monocarboxylate transporter (*T. equinum*)
TERG_00735	2.22	2.83		hypothetical protein
TERG_11976	2.21	2.73		integral membrane protein (*T. tonsurans*)
TERG_00379	2.18	1.52		integral membrane protein (*T. equinum*)
TERG_03826	2.18	1.62		hypothetical protein

TERG_03827	2.18	2.13	hypothetical protein
TERG_03800	2.12	2.76	hypothetical protein
TERG_02513	2.09	1.70	TAM domain methyltransferase (*T. equinum*)
TERG_01848	2.04	2.08	integral membrane protein (*M. gypseum*)
TERG_00119	2.03	3.95	CAMK/CAMKL/GIN4 protein kinase
TERG_00734	1.99	2.07	dipeptidase (*T. tonsurans*)
TERG_03847	1.99	2.45	methyltransferase SirN-like, putative (*T. verrucosum*)
TERG_01357	1.96	2.62	hypothetical protein
TERG_07116	1.93	2.37	aldehyde dehydrogenase (*T. tonsurans*)
TERG_08114	1.92	2.69	anaphase-promoting complex component Cut20/Apc4 (*T. tonsurans*)
TERG_02842	1.91	1.63	6-hydroxy-D-nicotine oxidase (*T. equinum*)
TERG_01436	1.88	1.56	oxidoreductase (*T. equinum*)
TERG_08029	1.82	2.30	HMG box protein, putative (*T. verrucosum*)
TERG_03343	1.81	2.91	fatty acid desaturase (*T. equinum*)
TERG_02510	1.79	2.80	hypothetical protein
TERG_05259	1.79	1.52	cytochrome P450, putative (*A. benhamiae*)
TERG_03719	1.78	1.83	MFS sugar transporter (*T. tonsurans*)
TERG_03820	1.74	2.19	hypothetical protein
TERG_02286	1.71	1.79	cell wall organization protein/glutathione transferase (Gto3), putative (*T. verrucosum*)
TERG_03024	1.71	1.51	hypothetical protein
TERG_03854	1.70	1.93	laccase (*T. tonsurans*)
TERG_07059	1.69	1.58	hypothetical protein
TERG_00828	1.69	1.58	hypothetical protein
TERG_06748	1.62	2.16	hypothetical protein
TERG_03253	1.61	1.77	hypothetical protein
TERG_08115	1.59	1.55	hypothetical protein
TERG_05430	1.57	1.97	hypothetical protein
TERG_00599	1.51	1.99	hypothetical protein
TERG_12059	1.51	2.56	transporter smf2 (*T. tonsurans*)
TERG_06397	-1.50	-1.56	alpha-1,2-mannosyltransferase (*T. equinum*)
TERG_01271	-1.52	-1.52	isocitrate lyase
TERG_12200	-1.53	-1.50	hypothetical protein
TERG_01156	-1.54	-1.72	integral membrane protein (*T. equinum*)
TERG_01325	-1.58	-2.50	hypothetical protein
TERG_06603	-1.59	-1.77	hypothetical protein
TERG_06889	-1.61	-1.72	cystathionine gamma-synthase (*T. tonsurans*)
TERG_03597	-1.63	-1.77	integral membrane protein (*T. tonsurans*)
TERG_03279	-1.64	-2.38	C6 finger domain-containing protein (*T. tonsurans*)
TERG_07501	-1.65	-2.42	nitrate reductase (*T. tonsurans*)
TERG_02545	-1.68	-2.30	MFS monocarboxylate transporter (*T. equinum*)
TERG_04339	-1.69	-2.06	signal transduction protein Syg1, putative (*A. benhamiae*)
TERG_00041	-1.74	-1.74	hypothetical protein
TERG_01299	-1.79	-1.81	DUF1613 domain-containing protein (*T. equinum*)

TERG_06801	-1.83	-1.83		cyclic nucleotide-binding domain-containing protein (*T. tonsurans*)
TERG_07161	-1.85	-1.86		hypothetical protein
TERG_01018	-1.86	-2.63		monooxygenase, putative (*A. benhamiae*)
TERG_01727	-1.91	-1.97		short-chain dehydrogenase (*T. equinum*)
TERG_07716	-1.92	-1.76		hypothetical protein
TERG_03226	-1.94	-2.08		glucosamine-6-phosphate deaminase
TERG_08059	-1.94	-1.86		sugar transporter (*T. equinum*)
TERG_08436	-2.00	-1.50		hypothetical protein
TERG_03593	-2.06	-1.96		hypothetical protein
TERG_02705	-2.11	-2.98		class III chitinase (*T. tonsurans*)
TERG_08787	-2.22	-3.03		aminotransferase (*T. tonsurans*)
TERG_07984	-2.27	-2.78		hypothetical protein
TERG_03221	-2.30	-2.18		C6 transcription factor, putative (*A. benhamiae*)
TERG_11958	-2.39	-3.07		superoxide dismutase copper chaperone Lys7 (*T. tonsurans*)
TERG_11964	-2.43	-2.59		C6 transcription factor, putative (*T. verrucosum*)
TERG_00479	-2.54	-3.13		hypothetical protein
TERG_12233	-2.57	-2.71		hypothetical protein
TERG_06276	-2.58	-2.36		chromate ion transporter (*T. tonsurans*)
TERG_01480	-2.93	-2.07		MFS multidrug transporter, putative (*A. benhamiae*)
TERG_01581	-3.03	-3.26		MFS multidrug transporter (*T. equinum*)
TERG_00172	-3.06	-3.01		hypothetical protein
TERG_03223	-3.10	-3.04		N-acetylglucosamine-6-phosphate deacetylase
TERG_05652	-3.31	-3.15		leucine aminopeptidase 1
TERG_12566	-3.60	-3.64		hypothetical protein

Gene expression values are expressed in log_2 fold change.

ID	24 hours	48 hours	96 hours	Gene Product Name
TERG_07028	7.30		2.55	hypothetical protein
TERG_08503	3.35		2.49	secalin (*M. canis*)
TERG_08353	3.20		2.41	cytochrome P450 55A3 (*T. tonsurans*)
TERG_02583	3.13		1.57	phosphate permease (*A. benhamiae*)
TERG_05091	2.59		2.79	DUF636 domain-containing protein (*T. equinum*)
TERG_06690	2.46		2.32	RTA1 domain protein, putative (*A. benhamiae*)
TERG_01979	2.35		1.74	pirin domain protein, putative (*A. benhamiae*)
TERG_02384	1.95		2.31	hypothetical protein
TERG_03179	1.89		-1.78	NlpC/P60-like cell-wall peptidase, putative (*A. benhamiae*)
TERG_06870	1.87		3.20	heat shock protein (*T. tonsurans*)
TERG_01352	1.78		1.95	benzoate 4-monooxygenase cytochrome P450 (*T. equinum*)
TERG_03070	1.77		1.85	flotillin domain-containing protein (*T. equinum*)
TERG_06675	1.75		1.62	GliK protein (*T. equinum*)
TERG_07973	1.74		3.76	CORD and CS domain-containing protein (*T. tonsurans*)

TERG_03730	1.63	1.70	RTA1 domain-containing protein (*T. equinum*)
TERG_07831	1.57	1.77	GNAT family acetyltransferase, putative (*T. verrucosum*)
TERG_00162	1.56	1.56	MFS multidrug transporter, putative (*A. benhamiae*)
TERG_04402	1.54	2.92	glyceraldehyde-3-phosphate dehydrogenase
TERG_04744	1.52	3.90	DNA damage-inducible protein 1 (*T. equinum*)
TERG_07417	-1.55	-1.98	hypothetical protein
TERG_08261	-1.57	-3.83	glutamate decarboxylase
TERG_02886	-1.59	-1.94	hypothetical protein
TERG_00608	-1.60	-1.59	hypothetical protein
TERG_12126	-1.62	-2.01	hypothetical protein
TERG_00471	-1.67	-1.67	hypothetical protein
TERG_08363	-1.68	-2.25	tyrosinase (*T. verrucosum*)
TERG_03948	-1.71	-2.77	hypothetical protein
TERG_00517	-1.71	-1.71	hypothetical protein
TERG_06986	-1.73	-2.12	exo-beta-1,3-glucanase, putative (*A. benhamiae*)
TERG_07798	-1.74	-1.62	hypothetical protein
TERG_01518	-1.81	-1.86	hypothetical protein
TERG_03544	-1.84	-1.55	CMGC/RCK/MAK protein kinase
TERG_07094	-1.85	-1.52	hypothetical protein
TERG_06290	-1.93	-1.87	hypothetical protein
TERG_00037	-1.98	-2.06	CHY zinc finger domain containing protein (*T. equinum*)
TERG_06295	-2.02	-3.19	C2H2 finger domain-containing protein (*T. tonsurans*)
TERG_01749	-2.06	-2.15	hypothetical protein
TERG_02132	-2.18	-1.77	5-histidylcysteine sulfoxide synthase
TERG_00569	-2.30	-1.78	C2H2 finger domain protein, putative (*A. benhamiae*)
TERG_02371	-2.36	-2.19	hypothetical protein
TERG_02134	-2.40	-3.48	indoleamine 2,3-dioxygenase (*T. equinum*)
TERG_01435	-2.45	-2.57	flavin containing polyamine oxidase, putative (*A. benhamiae*)
TERG_01360	-2.71	-1.59	C6 transcription factor, putative (*T. verrucosum*)
TERG_01490	-2.82	-3.42	LysR family regulatory protein (*M. gypseum*)
TERG_06242	-3.67	-3.14	glucanase, putative (*T. verrucosum*)

Gene expression values are expressed in log_2 fold change.

**ID**	**24 hours**	**48 hours**	**96 hours**	**Gene Product Name**
TERG_07357		7.63	4.69	hypothetical protein
TERG_03060		4.05	3.25	hypothetical protein
TERG_05139		3.03	2.56	non ribosomal peptide synthase (*T. equinum*)
TERG_03313		2.74	2.22	fucose-specific lectin FleA (*A. benhamiae*)
TERG_01802		2.56	2.53	hypothetical protein
TERG_11506		2.56	1.93	phosphoenolpyruvate carboxykinase [ATP]
TERG_03174		2.49	3.29	MFS siderochrome iron transporter MirB (*T. verrucosum*)
TERG_01879		2.44	2.76	hypothetical protein
TERG_11916		2.39	2.15	nucleolus protein required for cell viability (*T. tonsurans*)

TERG_12604	2.34	2.01	hypothetical protein
TERG_11915	2.31	2.13	nucleolus protein required for cell viability (*T. tonsurans*)
TERG_11505	2.27	1.87	phosphoenolpyruvate carboxykinase [ATP]
TERG_11646	2.23	2.67	hypothetical protein
TERG_00499	2.21	5.11	hypothetical protein
TERG_12369	2.13	1.57	MFS multidrug transporter (*T. tonsurans*)
TERG_04310	2.12	3.71	alcohol dehydrogenase (*T. tonsurans*)
TERG_12370	2.01	1.71	MFS multidrug transporter, putative (*A. benhamiae*)
TERG_12601	2.00	3.49	4-coumarate-CoA ligase (*T. tonsurans*)
TERG_00535	1.97	1.98	calcium-translocating P-type ATPase, PMCA-type
TERG_03602	1.96	1.58	Polysaccharide deacetylase (*T. tonsurans*)
TERG_11941	1.94	3.68	MCAK-like kinesin (*T. equinum*)
TERG_04994	1.92	1.86	pre-rRNA-processing protein ESF1 (*T. equinum*)
TERG_03528	1.89	2.05	cytochrome c
TERG_01273	1.85	2.43	alpha-1,2-mannosidase, putative subfamily (*T. verrucosum*)
TERG_02611	1.81	1.61	WD repeat protein (*A. benhamiae*)
TERG_00611	1.77	1.70	para-aminobenzoate synthase PabaA (*T. verrucosum*)
TERG_05242	1.76	1.83	PUF6 (*M. gypseum*)
TERG_07329	1.74	1.99	TPR domain-containing protein (*T. tonsurans*)
TERG_07072	1.73	1.52	ATP-dependent RNA helicase DRS1
TERG_11773	1.70	1.98	hypothetical protein
TERG_05434	1.69	1.75	ribosome associated DnaJ chaperone Zuotin (*T. equinum*)
TERG_00615	1.69	2.05	HLH transcription factor, putative (*A. benhamiae*)
TERG_02105	1.67	2.74	hypothetical protein
TERG_07595	1.66	2.24	hypothetical protein
TERG_01446	1.64	2.01	GMP synthase [glutamine-hydrolyzing]
TERG_01703	1.61	3.19	cytochrome P450 51 (*T. equinum*)
TERG_02956	1.60	1.61	ATP-dependent RNA helicase MAK5 (*T. tonsurans*)
TERG_06917	1.60	1.68	elongation factor G, mitochondrial
TERG_02107	1.59	1.89	protein phosphatase type 1 complex subunit Hex2/Reg1, putative (*T. verrucosum*)
TERG_02278	1.59	1.98	hypothetical protein
TERG_03172	1.56	1.88	sodium/phosphate symporter (*T. tonsurans*)
TERG_00385	1.56	1.77	oxidoreductase (*T. equinum*)
TERG_02788	1.55	1.62	DNA-directed RNA polymerase I (*T. tonsurans*)
TERG_07934	1.55	2.81	hypothetical protein
TERG_07403	1.55	1.57	U3 small nucleolar RNA-associated protein Utp11 (*T. tonsurans*)
TERG_08545	1.53	3.44	C-4 methyl sterol oxidase Erg25 (*T. equinum*)
TERG_08104	1.53	2.31	potassium/sodium efflux P-type ATPase, fungal-type
TERG_04425	1.53	1.86	deoxyhypusine synthase
TERG_03206	1.53	1.78	hsp7-like protein
TERG_07012	1.52	1.62	rRNA 2'-O-methyltransferase fibrillarin
TERG_12664	1.52	1.78	adenosine deaminase (*T. tonsurans*)

TERG_08066	1.51	1.84	STE/STE7 protein kinase
TERG_11641	1.50	1.58	forkhead transcription factor (*T. tonsurans*)
TERG_05909	-1.50	-1.65	hypothetical protein
TERG_03714	-1.50	-1.59	NCS1 nucleoside transporter (*T. tonsurans*)
TERG_12524	-1.51	-2.37	fatty acid oxygenase (*T. tonsurans*)
TERG_06199	-1.52	-1.91	lipase (*T. tonsurans*)
TERG_01197	-1.52	-1.56	nitrilase (*T. tonsurans*)
TERG_06177	-1.52	-2.37	mitochondrial carrier protein, putative (*T. verrucosum*)
TERG_01551	-1.53	-1.51	hypothetical protein
TERG_04605	-1.53	-1.84	progesterone binding protein (*T. tonsurans*)
TERG_00702	-1.54	-2.46	hypothetical protein
TERG_12613	-1.55	-2.30	succinyl-CoA synthetase beta subunit, putative (*A. benhamiae*)
TERG_12498	-1.56	-1.74	2-deoxy-D-gluconate 3-dehydrogenase (*T. equinum*)
TERG_02481	-1.56	-1.55	F-box domain-containing protein (*M. canis*)
TERG_02336	-1.56	-2.40	hypothetical protein
TERG_06944	-1.57	-1.65	guanyl-specific ribonuclease Th1
TERG_04232	-1.58	-2.86	2-nitropropane dioxygenase (*T. equinum*)
TERG_05895	-1.58	-2.30	MFS transporter (*M. canis*)
TERG_06234	-1.59	-1.61	2,5-diketo-D-gluconic acid reductase A (*T. equinum*)
TERG_04389	-1.60	-2.53	carnitinyl-CoA dehydratase (*T. tonsurans*)
TERG_02249	-1.62	-1.69	phenylacetyl-CoA ligase (*T. tonsurans*)
TERG_03483	-1.62	-2.79	carnitine acetyl transferase (*T. tonsurans*)
TERG_03229	-1.65	-1.77	hexokinase (*T. tonsurans*)
TERG_00449	-1.65	-2.34	mandelate racemase/muconate lactonizing enzyme family protein (*T. equinum*)
TERG_01259	-1.66	-1.65	hypothetical protein
TERG_03510	-1.67	-2.06	peroxisomal membrane anchor protein, putative (*A. benhamiae*)
TERG_08908	-1.68	-2.03	hypothetical protein
TERG_02380	-1.69	-1.69	hypothetical protein
TERG_08951	-1.70	-1.89	hypothetical protein
TERG_07691	-1.70	-1.86	sterol carrier protein (*T. tonsurans*)
TERG_04837	-1.71	-1.97	thioesterase (*T. equinum*)
TERG_07214	-1.72	-2.10	DlpA domain-containing protein (*T. equinum*)
TERG_00823	-1.74	-2.87	rhamnolipids biosynthesis 3-oxoacyl-[acyl-carrier-protein] reductase (*T. equinum*)
TERG_11678	-1.74	-1.96	solute carrier family 25 member 45 (*M. gypseum*)
TERG_04038	-1.74	-2.98	acyl-CoA dehydrogenase (*T. tonsurans*)
TERG_01937	-1.76	-2.24	short chain dehydrogenase (*T. tonsurans*)
TERG_11593	-1.77	-1.51	carboxypeptidase Y, putative (*A. benhamiae*)
TERG_02993	-1.77	-2.13	allergenic cerato-platanin Asp F13 (*T. verrucosum*)
TERG_02615	-1.78	-2.75	oxidoreductase, 2OG-Fe(II) oxygenase family, putative (*A. benhamiae*)
TERG_05224	-1.78	-1.70	hypothetical protein
TERG_01759	-1.78	-1.89	peroxisome assembly protein 10 (*T. equinum*)

TERG_05621	-1.80	-2.89	short chain dehydrogenase/reductase family oxidoreductase, putative (*A. benhamiae*)
TERG_12697	-1.82	-2.25	serine/threonine protein kinase (*T. tonsurans*)
TERG_05446	-1.82	-1.96	sarcosine oxidase (*T. equinum*)
TERG_03832	-1.82	-2.41	enoyl-CoA hydratase/isomerase (*T. equinum*)
TERG_04427	-1.83	-1.66	serine/threonine protein kinase
TERG_04213	-1.85	-2.90	TAM domain methyltransferase (*T. equinum*)
TERG_07607	-1.85	-3.17	hypothetical protein
TERG_01134	-1.87	-1.63	metalloprotease MEP1 (*A. benhamiae*)
TERG_03212	-1.88	-2.00	hypothetical protein
TERG_01742	-1.89	-2.38	hypothetical protein
TERG_02350	-1.89	-1.52	endochitinase (*T. equinum*)
TERG_04303	-1.89	-2.65	hypothetical protein
TERG_06114	-1.90	-1.74	hypothetical protein
TERG_11639	-1.90	-2.50	isocitrate lyase
TERG_00254	-1.91	-2.29	peroxisomal dehydratase (*T. equinum*)
TERG_03881	-1.92	-3.00	epoxide hydrolase (*T. tonsurans*)
TERG_07874	-1.94	-2.13	Ser/Thr protein phosphatase (*M. canis*)
TERG_02004	-1.95	-1.99	ureidoglycolate hydrolase, putative (*T. verrucosum*)
TERG_04279	-1.95	-2.46	YhhN domain-containing protein (*T. tonsurans*)
TERG_02902	-1.96	-1.61	hypothetical protein
TERG_04588	-1.98	-2.04	phosphoribulokinase/uridine kinase (*T. tonsurans*)
TERG_07088	-1.98	-1.66	glutamyl-tRNA(Gln) amidotransferase subunit A, putative (*T. verrucosum*)
TERG_01430	-1.98	-2.52	hypothetical protein
TERG_01463	-1.99	-2.06	cytochrome c peroxidase (*T. tonsurans*)
TERG_06779	-1.99	-1.96	hypothetical protein
TERG_07352	-2.02	-2.85	aldehyde reductase (AKR1), putative (*T. verrucosum*)
TERG_01784	-2.04	-2.94	oxidoreductase, short-chain dehydrogenase/reductase family, putative (*T. verrucosum*)
TERG_06726	-2.06	-1.84	O-methylsterigmatocystin oxidoreductase, putative (*A. benhamiae*)
TERG_02728	-2.10	-1.68	3-hydroxyisobutyrate dehydrogenase
TERG_01233	-2.12	-3.00	3,2-trans-enoyl-CoA isomerase (*T. equinum*)
TERG_02912	-2.12	-1.91	MFS transporter, putative (*A. benhamiae*)
TERG_05484	-2.15	-1.94	acyl-CoA dehydrogenase (*T. tonsurans*)
TERG_01184	-2.16	-2.40	fatty acid-binding protein (*T. equinum*)
TERG_11638	-2.17	-2.34	isocitrate lyase
TERG_01504	-2.17	-1.57	hypothetical protein
TERG_00570	-2.17	-2.41	autophagy protein (*T. equinum*)
TERG_04610	-2.18	-3.34	hypothetical protein
TERG_05111	-2.18	-1.75	hypothetical protein
TERG_03991	-2.18	-2.85	phosphotransferase enzyme family protein (*T. verrucosum*)
TERG_00587	-2.19	-2.23	nuclear pore complex protein sonA (*T. equinum*)
TERG_04003	-2.22	-2.77	AMP dependent CoA ligase (*T. tonsurans*)

TERG_07215		-2.22	-1.78	N-acetyltransferase family protein, putative (*A. benhamiae*)
TERG_00127		-2.23	-3.78	secretory phospholipase A2 (*T. tonsurans*)
TERG_12028		-2.25	-1.92	metallothionein family protein (*T. equinum*)
TERG_02909		-2.29	-3.83	acyl-CoA oxidase, putative (*T. verrucosum*)
TERG_12298		-2.33	-2.24	hypothetical protein
TERG_07721		-2.39	-2.97	isopenicillin N-CoA epimerase (*T. equinum*)
TERG_04414		-2.41	-3.33	isoflavone reductase family protein (CipA), putative (*A. benhamiae*)
TERG_07982		-2.42	-2.44	hypothetical protein
TERG_05843		-2.46	-2.40	F-box domain-containing protein (*T. equinum*)
TERG_11637		-2.48	-2.50	isocitrate lyase
TERG_08336		-2.49	-2.04	MFS multidrug transporter, putative (*A. benhamiae*)
TERG_12035		-2.51	-2.15	NB-ARC and TPR domain protein (*A. benhamiae*)
TERG_04721		-2.54	-1.80	glutamate carboxypeptidase (*T. equinum*)
TERG_03254		-2.56	-4.78	hypothetical protein
TERG_00801		-2.56	-3.45	hypothetical protein
TERG_07796		-2.56	-3.08	oxidoreductase, short-chain dehydrogenase/reductase family (*A. benhamiae*)
TERG_12029		-2.58	-2.62	hypothetical protein
TERG_02722		-2.62	-1.59	WSC domain containing protein (*T. equinum*)
TERG_03305		-2.67	-3.03	C-4 methylsterol oxidase (*T. tonsurans*)
TERG_02199		-2.74	-1.79	glutamate carboxypeptidase (*T. equinum*)
TERG_06679		-2.79	-1.72	MFS transporter, putative (*A. benhamiae*)
TERG_05816		-2.81	-2.07	hypothetical protein
TERG_08523		-2.84	-2.81	xylosidase, putative (*A. benhamiae*)
TERG_05239		-2.87	-1.94	DNA polymerase POL4, putative (*T. verrucosum*)
TERG_04066		-2.93	-3.60	filamentation protein (Rhf1), putative (*T. verrucosum*)
TERG_08614		-2.96	-4.74	hypothetical protein
TERG_03448		-3.13	-2.87	hypothetical protein
TERG_11892		-3.29	-3.95	hypothetical protein
TERG_00233		-3.84	-2.40	glyoxalase (*T. tonsurans*)
TERG_02449		-4.01	-2.26	phosphoadenosine phosphosulfate reductase
TERG_00060		-5.16	-4.32	GPI anchored cell wall protein, putative (*A. benhamiae*)
TERG_00235		-6.67	-8.18	hypothetical protein

Gene expression values are expressed in log_2 fold change.

ID	24 hours	48 hours	96 hours	Gene Product Name
TERG_06585	8.28	6.71	4.87	hypothetical protein
TERG_01915	6.06	4.95	2.99	O-methyltransferase (*A. benhamiae*)
TERG_12594	5.01	5.33	3.34	oxidoreductase, short-chain dehydrogenase/reductase family (*T. verrucosum*)
TERG_01917	4.57	3.66	2.30	O-methyltransferase, putative (*A. benhamiae*)
TERG_05442	4.56	3.05	2.09	hypothetical protein
TERG_11528	4.55	3.97	3.23	tenascin C (*T. equinum*)

TERG_01914	4.44	4.10	2.53	trichodiene oxygenase (*T. equinum*)
TERG_11527	4.39	3.55	3.46	tenascin C (*T. equinum*)
TERG_03345	4.17	6.11	3.13	hypothetical protein
TERG_11526	4.08	3.70	3.53	tenascin C (*T. equinum*)
TERG_00371	3.99	3.68	3.83	ferric-chelate reductase (*T. tonsurans*)
TERG_11747	3.74	3.30	3.53	hypothetical protein
TERG_03707	3.69	2.54	2.59	geranylgeranyl diphosphate synthase, putative (*A. benhamiae*)
TERG_08046	3.58	4.18	2.66	beta-lactamase (*M. gypseum*)
TERG_06076	3.24	3.04	2.32	hypothetical protein
TERG_01923	3.23	3.29	3.04	hypothetical protein
TERG_00713	3.21	2.48	2.48	hypothetical protein
TERG_03706	3.20	2.09	2.20	trichodiene oxygenase (*T. equinum*)
TERG_01912	3.19	2.70	4.02	MFS transporter (*T. equinum*)
TERG_11711	3.18	3.51	2.94	hypothetical protein
TERG_07847	2.93	2.26	1.94	hypothetical protein
TERG_02412	2.91	2.22	2.98	HHE domain protein (*T. verrucosum*)
TERG_03274	2.74	2.84	2.28	ammonium transporter MepA (*A. benhamiae*)
TERG_12090	2.71	2.43	2.50	hypothetical protein
TERG_06022	2.67	3.28	1.64	hypothetical protein
TERG_04169	2.63	2.96	2.60	DUF829 domain protein (PaxU), putative (*A. benhamiae*)
TERG_06767	2.62	2.52	1.62	aminopeptidase (*T. tonsurans*)
TERG_05141	2.53	4.96	4.16	NCS1 nucleoside transporter (*T. equinum*)
TERG_11866	2.49	2.18	2.17	hypothetical protein
TERG_07364	2.47	2.85	2.36	oxidoreductase, zinc-binding dehydrogenase family superfamily (*T. verrucosum*)
TERG_12532	2.40	3.45	2.20	hypothetical protein
TERG_04960	2.20	3.95	3.24	glutathione S-transferase Ure2-like, putative (*A. benhamiae*)
TERG_12627	2.18	2.34	1.99	C-5 sterol desaturase (*T. equinum*)
TERG_12125	2.15	1.91	2.22	hypothetical protein
TERG_12626	2.12	2.42	1.99	C-5 sterol desaturase (*T. equinum*)
TERG_00694	2.07	1.87	2.85	glutamate 5-kinase
TERG_03390	2.07	2.10	4.25	glutation S-transferase (*T. equinum*)
TERG_05850	2.04	1.95	1.93	integral membrane protein (*A. benhamiae*)
TERG_07237	2.03	1.59	3.03	hsp90-like protein (*T. equinum*)
TERG_03791	2.03	2.83	3.95	NADH-ubiquinone oxidoreductase 20 kDa subunit (*T. equinum*)
TERG_07220	2.02	2.71	1.81	hypothetical protein
TERG_05260	2.02	1.66	1.99	general amidase GmdB (*A. benhamiae*)
TERG_01901	2.00	2.68	2.13	glycerol kinase
TERG_07795	1.94	1.75	1.92	WSC domain protein, putative (*A. benhamiae*)
TERG_05124	1.93	2.55	2.44	gliotoxin biosynthesis protein GliK (*T. verrucosum*)
TERG_11517	1.85	2.46	1.73	hypothetical protein
TERG_01911	1.85	1.83	2.45	NmrA family protein (*T. equinum*)
TERG_12623	1.83	2.10	3.53	glutaredoxin domain-containing protein (*T. equinum*)

TERG_11814	1.80	1.56	1.56	FAD dependent oxidoreductase, putative (*A. benhamiae*)
TERG_12602	1.72	2.60	2.31	4-coumarate-CoA ligase (*T. equinum*)
TERG_06755	1.66	1.74	3.38	C-8 sterol isomerase (Erg-1), putative (*T. verrucosum*)
TERG_12392	1.66	1.57	1.84	hypothetical protein
TERG_01900	1.66	3.07	2.47	aquaglyceroporin, putative (*A. benhamiae*)
TERG_00583	1.62	1.70	1.81	hypothetical protein
TERG_04516	1.61	2.33	1.77	hypothetical protein
TERG_07099	1.58	3.17	4.17	hypothetical protein
TERG_00205	1.56	1.88	2.21	alpha-tubulin suppressor protein Aats1 (*T. tonsurans*)
TERG_07661	-1.52	-1.83	-1.91	mannosylphosphate transferase (*T. equinum*)
TERG_03367	-1.53	-2.99	-2.71	hypothetical protein
TERG_02368	-1.54	-1.95	-3.15	extracellular developmental signal biosynthesis protein FluG (*A. benhamiae*)
TERG_01272	-1.54	-1.85	-1.58	2-methylcitrate synthase, mitochondrial
TERG_12167	-1.55	-2.04	-2.35	deoxycytidylate deaminase (*T. equinum*)
TERG_05151	-1.58	-1.55	-2.12	hypothetical protein
TERG_06355	-1.58	-3.16	-1.87	acetyltransferase, GNAT family (*A. benhamiae*)
TERG_12166	-1.59	-2.04	-2.49	deoxycytidylate deaminase (*T. equinum*)
TERG_05439	-1.59	-1.61	-1.98	IMP-specific 5'-nucleotidase (*T. equinum*)
TERG_06196	-1.63	-1.71	-1.80	hypothetical protein
TERG_05706	-1.65	-1.67	-1.56	hypothetical protein
TERG_03148	-1.66	-1.91	-1.67	molybdenum cofactor sulfurase (*T. equinum*)
TERG_06233	-1.67	-3.15	-1.57	hypothetical protein
TERG_02609	-1.67	-2.40	-1.62	actin patches distal protein 1 (*T. equinum*)
TERG_04793	-1.67	-1.51	-1.60	cyclin (*T. tonsurans*)
TERG_05899	-1.69	-2.84	-2.83	ubiE/COQ5 methyltransferase, putative (*A. benhamiae*)
TERG_01503	-1.71	-2.24	-1.76	DUF895 domain membrane protein (*T. verrucosum*)
TERG_05456	-1.71	-2.54	-2.29	hypothetical protein
TERG_08909	-1.72	-2.19	-2.31	hypothetical protein
TERG_06992	-1.75	-1.90	-1.92	pyridine nucleotide-disulphide oxidoreductase (*T. tonsurans*)
TERG_04289	-1.76	-1.81	-1.59	hypothetical protein
TERG_07238	-1.76	-1.73	-2.34	zinc-binding oxidoreductase, putative (*A. benhamiae*)
TERG_08173	-1.78	-1.64	-2.30	C6 finger domain protein, putative (*A. benhamiae*)
TERG_02913	-1.78	-2.17	-1.66	LIM domain protein (*T. verrucosum*)
TERG_11852	-1.79	-1.91	-1.99	glyoxalase family protein (*T. verrucosum*)
TERG_07963	-1.79	-2.02	-2.65	hypothetical protein
TERG_12372	-1.81	-1.59	-1.96	multidrug resistance protein (*T. tonsurans*)
TERG_06261	-1.81	-2.13	-2.40	phosphoesterase (*T. equinum*)
TERG_07326	-1.83	-3.51	-5.62	glutathione S-transferase PARB (*T. equinum*)
TERG_02262	-1.83	-1.67	-1.78	NACHT and WD domain protein (*T. verrucosum*)
TERG_01194	-1.83	-3.67	-3.59	uracil DNA glycosylase (*T. tonsurans*)
TERG_07780	-1.84	-2.57	-2.22	hypothetical protein
TERG_08709	-1.86	-3.06	-4.28	SesA protein (*T. equinum*)
TERG_01433	-1.87	-1.94	-2.57	WD domain, G-beta repeat protein (*A. benhamiae*)
TERG_00527	-1.87	-2.28	-1.94	hypothetical protein

TERG_05669	-1.88	-1.72	-2.66	homeobox transcription factor, putative (*T. verrucosum*)
TERG_05627	-1.88	-2.61	-1.64	LysM domain-containing protein (*M. canis*)
TERG_11541	-1.88	-2.10	-1.97	hypothetical protein
TERG_08708	-1.91	-1.68	-3.05	SesA protein (*T. equinum*)
TERG_05744	-1.93	-1.77	-2.19	GTP-binding protein EsdC (*T. equinum*)
TERG_02589	-1.96	-1.58	-2.60	formamidase (*T. equinum*)
TERG_05809	-1.97	-1.67	-3.27	hypothetical protein
TERG_01657	-1.97	-2.27	-2.93	heat shock protein 30 (*T. equinum*)
TERG_01733	-1.98	-2.37	-2.23	alcohol dehydrogenase, putative (*T. verrucosum*)
TERG_03537	-1.99	-2.02	-1.90	alpha/beta hydrolase (*T. equinum*)
TERG_00995	-1.99	-1.83	-1.79	hypothetical protein
TERG_08182	-2.00	-5.14	-4.92	hypothetical protein
TERG_00349	-2.02	-2.48	-1.53	hypothetical protein
TERG_03699	-2.02	-2.64	-1.68	hypothetical protein
TERG_00483	-2.03	-2.94	-3.65	hypothetical protein
TERG_06389	-2.04	-1.82	-1.73	AhpA protein (*T. equinum*)
TERG_07597	-2.05	-2.18	-1.76	SOK1 (*T. equinum*)
TERG_12525	-2.06	-1.70	-3.39	fatty acid oxygenase (*T. tonsurans*)
TERG_08178	-2.08	-1.99	-3.17	endoglucanase (*T. equinum*)
TERG_03397	-2.08	-1.89	-1.96	hypothetical protein
TERG_01902	-2.09	-1.94	-2.31	phosphotransferase (*M. canis*)
TERG_03808	-2.12	-2.20	-2.57	hypothetical protein
TERG_02650	-2.13	-2.03	-3.05	NmrA family protein (*T. equinum*)
TERG_08174	-2.14	-2.19	-2.74	hypothetical protein
TERG_00820	-2.14	-2.68	-1.93	MFS multidrug transporter, putative (*A. benhamiae*)
TERG_04421	-2.15	-2.12	-2.07	hypothetical protein
TERG_02953	-2.16	-1.86	-2.02	hypothetical protein
TERG_11511	-2.16	-2.66	-2.28	hypothetical protein
TERG_01275	-2.18	-2.58	-1.60	hypothetical protein
TERG_03662	-2.19	-2.67	-3.32	hypothetical protein
TERG_03358	-2.22	-2.12	-3.87	hypothetical protein
TERG_06285	-2.24	-1.58	-1.70	hypothetical protein
TERG_12504	-2.25	-2.17	-2.18	MFS multidrug transporter (*T. equinum*)
TERG_12654	-2.28	-2.53	-2.12	hypothetical protein
TERG_05700	-2.28	-3.51	-2.68	hypothetical protein
TERG_11806	-2.30	-3.51	-3.00	hypothetical protein
TERG_01985	-2.31	-2.16	-3.31	hypothetical protein
TERG_07039	-2.32	-2.09	-1.74	hypothetical protein
TERG_02646	-2.34	-1.77	-3.19	hypothetical protein
TERG_07960	-2.37	-2.52	-3.51	hypothetical protein
TERG_12578	-2.38	-2.18	-4.69	PEP phosphonomutase (*T. equinum*)
TERG_12503	-2.40	-2.25	-1.76	polyamine transporter 2
TERG_02483	-2.41	-3.03	-2.84	hypothetical protein

TERG_04775	-2.45	-2.80	-1.99	beta-N-hexosaminidase, putative (*A. benhamiae*)
TERG_00916	-2.45	-3.66	-2.44	MFS sugar permease, putative (*A. benhamiae*)
TERG_02649	-2.46	-2.65	-4.10	benzoate 4-monooxygenase cytochrome P450 (*T. equinum*)
TERG_03019	-2.50	-2.24	-3.07	hypothetical protein
TERG_02725	-2.50	-2.71	-3.87	hypothetical protein
TERG_02493	-2.51	-2.42	-3.26	glyoxalase family protein (*T. verrucosum*)
TERG_00958	-2.51	-2.73	-2.91	amino acid permease (*T. tonsurans*)
TERG_05625	-2.51	-2.13	-1.60	class V chitinase, putative (*T. verrucosum*)
TERG_02263	-2.58	-2.44	-2.89	hypothetical protein
TERG_02588	-2.58	-1.87	-2.37	hypothetical protein
TERG_04867	-2.58	-1.77	-1.63	SAM and PH domain protein (Boi1), putative (*A. benhamiae*)
TERG_08969	-2.58	-2.33	-3.17	cytosolic Cu/Zn superoxide dismutase, putative (*A. benhamiae*)
TERG_07907	-2.61	-2.46	-1.84	hypothetical protein
TERG_02986	-2.62	-2.09	-3.69	hypothetical protein
TERG_06929	-2.63	-3.78	-2.11	chitinase (*T. equinum*)
TERG_11805	-2.63	-3.34	-2.42	hypothetical protein
TERG_03885	-2.65	-3.88	-2.57	hypothetical protein
TERG_02283	-2.66	-2.89	-2.42	MFS transporter, putative (*T. verrucosum*)
TERG_07135	-2.68	-2.69	-2.82	hypothetical protein
TERG_04680	-2.68	-1.72	-2.03	hypothetical protein
TERG_12022	-2.69	-1.74	-2.77	hypothetical protein
TERG_01840	-2.72	-2.27	-2.15	carboxypeptidase S1 (*T. equinum*)
TERG_01904	-2.77	-2.64	-2.77	hypothetical protein
TERG_05808	-2.77	-2.09	-4.24	hypothetical protein
TERG_03661	-2.79	-2.11	-2.49	hypothetical protein
TERG_06780	-2.82	-3.63	-5.06	hypothetical protein
TERG_07673	-2.83	-2.73	-2.29	hypothetical protein
TERG_02367	-2.88	-2.93	-2.54	hypothetical protein
TERG_02169	-2.89	-4.65	-4.54	carboxylesterase, putative (*A. benhamiae*)
TERG_07669	-2.91	-2.10	-1.66	carnitine acetyl transferase (*T. tonsurans*)
TERG_01959	-2.91	-2.76	-4.14	RNA polymerase Rpb1 C-terminal repeat domain-containing protein (*T. equinum*)
TERG_05943	-2.92	-5.81	-6.54	aminotransferase, class III (*T. verrucosum*)
TERG_03728	-2.92	-2.70	-3.89	hypothetical protein
TERG_08757	-2.93	-3.99	-3.60	hypothetical protein
TERG_08444	-2.95	-5.59	-5.20	CipC protein (*M. canis*)
TERG_06484	-2.97	-2.41	-2.93	MYB DNA-binding domain-containing protein (*T. tonsurans*)
TERG_03732	-2.97	-4.89	-5.09	hypothetical protein
TERG_04879	-3.02	-3.19	-5.68	hypothetical protein
TERG_07927	-3.05	-2.39	-3.31	hypothetical protein
TERG_03311	-3.11	-4.04	-5.63	hypothetical protein
TERG_07909	-3.11	-2.73	-4.17	isochorismatase family hydrolase, putative (*A. benhamiae*)
TERG_00819	-3.12	-2.77	-4.64	PE repeat family protein (*T. verrucosum*)
TERG_02369	-3.13	-2.78	-3.03	MFS transporter (*T. tonsurans*)
TERG_11640	-3.16	-3.43	-2.75	hypothetical protein

TERG_11630	-3.17	-3.13	-2.48	hypothetical protein
TERG_04742	-3.18	-3.44	-4.15	hypothetical protein
TERG_01950	-3.19	-3.95	-3.52	serine/threonine protein kinase (*T. tonsurans*)
TERG_01336	-3.20	-3.21	-3.06	MFS transporter (*T. equinum*)
TERG_06890	-3.29	-2.66	-2.50	DUF636 domain protein (*T. verrucosum*)
TERG_07923	-3.34	-2.78	-2.76	neutral amino acid permease (*T. equinum*)
TERG_01981	-3.35	-4.49	-4.35	hypothetical protein
TERG_06296	-3.41	-2.65	-3.34	hypothetical protein
TERG_12321	-3.49	-4.10	-3.74	hypothetical protein
TERG_00135	-3.49	-2.96	-3.16	metalloreductase transmembrane component (*T. equinum*)
TERG_01193	-3.50	-3.03	-2.59	PRO41 protein (*T. equinum*)
TERG_07079	-3.51	-3.47	-4.14	C6 transcription factor, putative (*T. verrucosum*)
TERG_05659	-3.64	-3.98	-3.97	hypothetical protein
TERG_11895	-3.67	-3.65	-5.07	polyketide synthase (*T. tonsurans*)
TERG_06267	-3.81	-2.45	-3.19	hypothetical protein
TERG_06807	-3.85	-4.62	-4.81	cutinase, putative (*A. benhamiae*)
TERG_04543	-3.85	-3.24	-2.51	aminotransferase GliI (*T. equinum*)
TERG_00911	-3.90	-4.46	-4.79	hypothetical protein
TERG_04282	-3.99	-4.79	-4.05	GPI ethanolamine phosphate transferase (*T. tonsurans*)
TERG_03459	-3.99	-3.05	-3.01	GDSL Lipase/Acylhydrolase family protein (*A. benhamiae*)
TERG_01279	-4.01	-4.28	-4.11	flavin-binding monooxygenase (*T. equinum*)
TERG_06854	-4.05	-4.88	-3.90	glutamine-serine-proline rich protein, putative (*A. benhamiae*)
TERG_06160	-4.10	-3.98	-3.94	nitrite reductase, copper-containing
TERG_02365	-4.13	-4.45	-2.81	hypothetical protein
TERG_00714	-4.19	-4.07	-4.67	cell pattern formation-associated protein (*T. tonsurans*)
TERG_01599	-4.42	-4.41	-4.41	hypothetical protein
TERG_06651	-4.59	-4.75	-3.29	cytochrome P450, putative (*T. verrucosum*)
TERG_06414	-5.02	-7.31	-7.38	DUF1338 domain-containing protein (*T. equinum*)
TERG_04738	-5.34	-5.00	-4.71	hypothetical protein
TERG_06802	-5.48	-4.85	-5.08	hypothetical protein

Gene expression values are expressed in $\log_2$ fold change.

Supplementary Table S6. The GO terms overrepresented in differentially expressed genes obtained from the comparison between Δ*stuA* versus wild-type (*p-value* < 0.05).

24 hours (Glucose)

Go term	Description	p-value	Class	Regulation	Genes
GO:0055114	oxidation-reduction process	0	BP	Down	39
GO:0055085	transmembrane transport	0	BP	Down	22
GO:0006357	regulation of transcription by RNA polymerase II	0.01	BP	Down	12
GO:0032259	methylation	0.02	BP	Down	12
GO:0008152	metabolic process	0.02	BP	Down	7
GO:0019752	carboxylic acid metabolic process	0	BP	Down	5
GO:0006807	nitrogen compound metabolic process	0	BP	Down	4
GO:0009820	alkaloid metabolic process	0.01	BP	Down	3
GO:0019441	tryptophan catabolic process to kynurenine	0	BP	Down	3
GO:0035835	indole alkaloid biosynthetic process	0	BP	Down	3
GO:0006536	glutamate metabolic process	0	BP	Down	2
GO:0006730	one-carbon metabolic process	0	BP	Down	2
GO:0008654	phospholipid biosynthetic process	0.01	BP	Down	2
GO:0009072	aromatic amino acid family metabolic process	0.01	BP	Down	2
GO:0009086	methionine biosynthetic process	0.02	BP	Down	2
GO:0019748	secondary metabolic process	0	BP	Down	2
GO:0043386	mycotoxin biosynthetic process	0.02	BP	Down	2
GO:0070941	eisosome assembly	0.01	BP	Down	2
GO:0000096	sulfur amino acid metabolic process	0.03	BP	Down	1
GO:0005976	polysaccharide metabolic process	0.01	BP	Down	1
GO:0005986	sucrose biosynthetic process	0.02	BP	Down	1
GO:0006002	fructose 6-phosphate metabolic process	0.03	BP	Down	1
GO:0006013	mannose metabolic process	0.02	BP	Down	1
GO:0006021	inositol biosynthetic process	0.02	BP	Down	1
GO:0006542	glutamine biosynthetic process	0.03	BP	Down	1
GO:0006571	tyrosine biosynthetic process	0.02	BP	Down	1
GO:0009094	L-phenylalanine biosynthetic process	0.04	BP	Down	1
GO:0009264	deoxyribonucleotide catabolic process	0.03	BP	Down	1
GO:0015976	carbon utilization	0.02	BP	Down	1
GO:0018105	peptidyl-serine phosphorylation	0.03	BP	Down	1
GO:0018107	peptidyl-threonine phosphorylation	0.01	BP	Down	1
GO:0019381	atrazine catabolic process	0.04	BP	Down	1
GO:0019499	cyanide metabolic process	0.01	BP	Down	1

GO:0019500	cyanide catabolic process	0.03	BP	Down	1
GO:0019510	S-adenosylhomocysteine catabolic process	0.03	BP	Down	1
GO:0019629	propionate catabolic process, 2-methylcitrate cycle	0.02	BP	Down	1
GO:0030261	chromosome condensation	0.04	BP	Down	1
GO:0030388	fructose 1,6-bisphosphate metabolic process	0.01	BP	Down	1
GO:0031124	mRNA 3'-end processing	0.02	BP	Down	1
GO:0035837	ergot alkaloid biosynthetic process	0.02	BP	Down	1
GO:0042276	error-prone translesion synthesis	0.02	BP	Down	1
GO:0042744	hydrogen peroxide catabolic process	0.01	BP	Down	1
GO:0044262	cellular carbohydrate metabolic process	0.04	BP	Down	1
GO:0045122	aflatoxin biosynthetic process	0.02	BP	Down	1
GO:0045461	sterigmatocystin biosynthetic process	0.01	BP	Down	1
GO:0046294	formaldehyde catabolic process	0.02	BP	Down	1
GO:0046386	deoxyribose phosphate catabolic process	0.01	BP	Down	1
GO:0046496	nicotinamide nucleotide metabolic process	0.01	BP	Down	1
GO:0046938	phytochelatin biosynthetic process	0.01	BP	Down	1
GO:0051289	protein homotetramerization	0.02	BP	Down	1
GO:0070814	hydrogen sulfide biosynthetic process	0.02	BP	Down	1
GO:0120029	proton export across plasma membrane	0.04	BP	Down	1
GO:0055085	transmembrane transport	0	BP	Up	17
GO:0008152	metabolic process	0	BP	Up	6
GO:0009405	pathogenesis	0.03	BP	Up	5
GO:0006865	amino acid transport	0	BP	Up	3
GO:0031505	fungal-type cell wall organization	0	BP	Up	3
GO:0006037	cell wall chitin metabolic process	0	BP	Up	2
GO:0016226	iron-sulfur cluster assembly	0	BP	Up	2
GO:0016573	histone acetylation	0.01	BP	Up	2
GO:0043386	mycotoxin biosynthetic process	0.01	BP	Up	2
GO:0044550	secondary metabolite biosynthetic process	0	BP	Up	2
GO:0071555	cell wall organization	0	BP	Up	2
GO:0002084	protein depalmitoylation	0.01	BP	Up	1
GO:0005992	trehalose biosynthetic process	0.03	BP	Up	1
GO:0006560	proline metabolic process	0.01	BP	Up	1
GO:0006562	proline catabolic process	0	BP	Up	1
GO:0006689	ganglioside catabolic process	0.03	BP	Up	1
GO:0006772	thiamine metabolic process	0.01	BP	Up	1
GO:0009229	thiamine diphosphate biosynthetic process	0.04	BP	Up	1

GO:0009313	oligosaccharide catabolic process	0.01	BP	Up	1
GO:0009423	chorismate biosynthetic process	0.04	BP	Up	1
GO:0009820	alkaloid metabolic process	0.04	BP	Up	1
GO:0016070	RNA metabolic process	0.04	BP	Up	1
GO:0017001	antibiotic catabolic process	0.04	BP	Up	1
GO:0030435	sporulation resulting in formation of a cellular spore	0.04	BP	Up	1
GO:0032509	endosome transport via multivesicular body sorting pathway	0.03	BP	Up	1
GO:0032981	mitochondrial respiratory chain complex I assembly	0.03	BP	Up	1
GO:0033609	oxalate metabolic process	0.02	BP	Up	1
GO:0034501	protein localization to kinetochore	0.01	BP	Up	1
GO:0035434	copper ion transmembrane transport	0.01	BP	Up	1
GO:0050482	arachidonic acid secretion	0	BP	Up	1
GO:0090481	pyrimidine nucleotide-sugar transmembrane transport	0.04	BP	Up	1
GO:0099116	tRNA 5'-end processing	0.01	BP	Up	1
GO:1902358	sulfate transmembrane transport	0.02	BP	Up	1

48 hours (Glucose)

Go term	Description	p-value	Class	Regulation	Genes
GO:0055114	oxidation-reduction process	0	BP	Down	64
GO:0055085	transmembrane transport	0	BP	Down	33
GO:0032259	methylation	0	BP	Down	18
GO:0008152	metabolic process	0.01	BP	Down	9
GO:0006807	nitrogen compound metabolic process	0.01	BP	Down	4
GO:0009820	alkaloid metabolic process	0	BP	Down	4
GO:0019752	carboxylic acid metabolic process	0.01	BP	Down	4
GO:0035835	indole alkaloid biosynthetic process	0	BP	Down	4
GO:0006979	response to oxidative stress	0.02	BP	Down	3
GO:0042744	hydrogen peroxide catabolic process	0	BP	Down	3
GO:0007062	sister chromatid cohesion	0.01	BP	Down	2
GO:0009072	aromatic amino acid family metabolic process	0.03	BP	Down	2
GO:0019441	tryptophan catabolic process to kynurenine	0.03	BP	Down	2
GO:0019748	secondary metabolic process	0.01	BP	Down	2
GO:0030245	cellulose catabolic process	0.02	BP	Down	2
GO:0045132	meiotic chromosome segregation	0	BP	Down	2
GO:0000070	mitotic sister chromatid segregation	0.04	BP	Down	1
GO:0000302	response to reactive oxygen species	0.04	BP	Down	1

GO:0005976	polysaccharide metabolic process	0.03	BP	Down	1
GO:0005991	trehalose metabolic process	0.04	BP	Down	1
GO:0005993	trehalose catabolic process	0.03	BP	Down	1
GO:0006021	inositol biosynthetic process	0.04	BP	Down	1
GO:0006097	glyoxylate cycle	0.04	BP	Down	1
GO:0006562	proline catabolic process	0.03	BP	Down	1
GO:0009168	purine ribonucleoside monophosphate biosynthetic process	0.02	BP	Down	1
GO:0009264	deoxyribonucleotide catabolic process	0.01	BP	Down	1
GO:0018107	peptidyl-threonine phosphorylation	0.03	BP	Down	1
GO:0019500	cyanide catabolic process	0.02	BP	Down	1
GO:0032264	IMP salvage	0.03	BP	Down	1
GO:0035672	oligopeptide transmembrane transport	0.02	BP	Down	1
GO:0035837	ergot alkaloid biosynthetic process	0.03	BP	Down	1
GO:0045461	sterigmatocystin biosynthetic process	0.04	BP	Down	1
GO:0046294	formaldehyde catabolic process	0.03	BP	Down	1
GO:0046386	deoxyribose phosphate catabolic process	0.02	BP	Down	1
GO:0048312	intracellular distribution of mitochondria	0.04	BP	Down	1
GO:0051289	protein homotetramerization	0.04	BP	Down	1
GO:0098863	nuclear migration by microtubule mediated pushing forces	0.03	BP	Down	1
GO:0055085	transmembrane transport	0	BP	Up	31
GO:0008152	metabolic process	0.01	BP	Up	8
GO:0009405	pathogenesis	0.02	BP	Up	6
GO:0042254	ribosome biogenesis	0.03	BP	Up	5
GO:0006812	cation transport	0	BP	Up	4
GO:0006865	amino acid transport	0.02	BP	Up	4
GO:0000272	polysaccharide catabolic process	0	BP	Up	3
GO:0006367	transcription initiation from RNA polymerase II promoter	0	BP	Up	3
GO:0016042	lipid catabolic process	0	BP	Up	3
GO:0031505	fungal-type cell wall organization	0	BP	Up	3
GO:0000027	ribosomal large subunit assembly	0.01	BP	Up	2
GO:0006031	chitin biosynthetic process	0	BP	Up	2
GO:0006032	chitin catabolic process	0.02	BP	Up	2
GO:0006037	cell wall chitin metabolic process	0	BP	Up	2
GO:0006816	calcium ion transport	0	BP	Up	2
GO:0009820	alkaloid metabolic process	0	BP	Up	2

GO:0015074	DNA integration	0.01	BP	Up	2
GO:0016226	iron-sulfur cluster assembly	0	BP	Up	2
GO:0019441	tryptophan catabolic process to kynurenine	0	BP	Up	2
GO:0043386	mycotoxin biosynthetic process	0.04	BP	Up	2
GO:0044550	secondary metabolite biosynthetic process	0	BP	Up	2
GO:0070588	calcium ion transmembrane transport	0	BP	Up	2
GO:0071555	cell wall organization	0.03	BP	Up	2
GO:0098655	cation transmembrane transport	0.02	BP	Up	2
GO:0099132	ATP hydrolysis coupled cation transmembrane transport	0.04	BP	Up	2
GO:0106074	aminoacyl-tRNA metabolism involved in translational fidelity	0.01	BP	Up	2
GO:0000447	endonucleolytic cleavage in ITS1 to separate SSU-rRNA from 5.8S rRNA and LSU-rRNA from tricistronic rRNA transcript (SSU-rRNA, 5.8S rRNA, LSU-rRNA)	0.04	BP	Up	1
GO:0000472	endonucleolytic cleavage to generate mature 5'-end of SSU-rRNA from (SSU-rRNA, 5.8S rRNA, LSU-rRNA)	0.03	BP	Up	1
GO:0000480	endonucleolytic cleavage in 5'-ETS of tricistronic rRNA transcript (SSU-rRNA, 5.8S rRNA, LSU-rRNA)	0.04	BP	Up	1
GO:0000917	division septum assembly	0.01	BP	Up	1
GO:0001172	transcription, RNA-templated	0.04	BP	Up	1
GO:0001510	RNA methylation	0.01	BP	Up	1
GO:0005976	polysaccharide metabolic process	0	BP	Up	1
GO:0006283	transcription-coupled nucleotide-excision repair	0	BP	Up	1
GO:0006400	tRNA modification	0.04	BP	Up	1
GO:0006429	leucyl-tRNA aminoacylation	0.02	BP	Up	1
GO:0006560	proline metabolic process	0.01	BP	Up	1
GO:0006562	proline catabolic process	0.01	BP	Up	1
GO:0006725	cellular aromatic compound metabolic process	0.02	BP	Up	1
GO:0006749	glutathione metabolic process	0.04	BP	Up	1
GO:0006750	glutathione biosynthetic process	0.01	BP	Up	1
GO:0006751	glutathione catabolic process	0.04	BP	Up	1
GO:0006817	phosphate ion transport	0.01	BP	Up	1
GO:0006874	cellular calcium ion homeostasis	0.04	BP	Up	1
GO:0007155	cell adhesion	0.02	BP	Up	1
GO:0007229	integrin-mediated signaling pathway	0.02	BP	Up	1
GO:0008360	regulation of cell shape	0	BP	Up	1
GO:0009098	leucine biosynthetic process	0.02	BP	Up	1

GO:0009229	thiamine diphosphate biosynthetic process	0	BP	Up	1
GO:0009272	fungal-type cell wall biogenesis	0.01	BP	Up	1
GO:0009313	oligosaccharide catabolic process	0.02	BP	Up	1
GO:0009712	catechol-containing compound metabolic process	0.04	BP	Up	1
GO:0016598	protein arginylation	0	BP	Up	1
GO:0031167	rRNA methylation	0.02	BP	Up	1
GO:0032220	plasma membrane fusion involved in cytogamy	0.02	BP	Up	1
GO:0032774	RNA biosynthetic process	0.01	BP	Up	1
GO:0043666	regulation of phosphoprotein phosphatase activity	0.04	BP	Up	1
GO:0045048	protein insertion into ER membrane	0.01	BP	Up	1
GO:0051607	defense response to virus	0	BP	Up	1
GO:0051666	actin cortical patch localization	0.01	BP	Up	1
GO:0071467	cellular response to pH	0.01	BP	Up	1
GO:0071816	tail-anchored membrane protein insertion into ER membrane	0	BP	Up	1
GO:0090481	pyrimidine nucleotide-sugar transmembrane transport	0.02	BP	Up	1
GO:0098703	calcium ion import across plasma membrane	0.02	BP	Up	1
GO:0099116	tRNA 5'-end processing	0.01	BP	Up	1
GO:1902358	sulfate transmembrane transport	0	BP	Up	1

96 hours (Glucose)

Go term	Description	p-value	Class	Regulation	Genes
GO:0055085	transmembrane transport	0.03	BP	Down	25
GO:0006355	regulation of transcription, DNA-templated	0	BP	Down	24
GO:0006357	regulation of transcription by RNA polymerase II	0	BP	Down	19
GO:0005975	carbohydrate metabolic process	0.01	BP	Down	11
GO:0008152	metabolic process	0	BP	Down	9
GO:0000272	polysaccharide catabolic process	0.01	BP	Down	4
GO:0043386	mycotoxin biosynthetic process	0	BP	Down	4
GO:0006807	nitrogen compound metabolic process	0.04	BP	Down	3
GO:0006865	amino acid transport	0.03	BP	Down	3
GO:0008654	phospholipid biosynthetic process	0.01	BP	Down	3
GO:0030245	cellulose catabolic process	0	BP	Down	3
GO:0070941	eisosome assembly	0	BP	Down	3
GO:0006094	gluconeogenesis	0.04	BP	Down	2
GO:0006646	phosphatidylethanolamine biosynthetic process	0	BP	Down	2

GO:0016540	protein autoprocessing	0	BP	Down	2
GO:0019344	cysteine biosynthetic process	0.03	BP	Down	2
GO:0019441	tryptophan catabolic process to kynurenine	0.01	BP	Down	2
GO:0019748	secondary metabolic process	0	BP	Down	2
GO:0000070	mitotic sister chromatid segregation	0.02	BP	Down	1
GO:0000096	sulfur amino acid metabolic process	0.03	BP	Down	1
GO:0005976	polysaccharide metabolic process	0.04	BP	Down	1
GO:0006075	(1->3)-beta-D-glucan biosynthetic process	0.02	BP	Down	1
GO:0006097	glyoxylate cycle	0.02	BP	Down	1
GO:0006108	malate metabolic process	0.02	BP	Down	1
GO:0006542	glutamine biosynthetic process	0.03	BP	Down	1
GO:0006560	proline metabolic process	0.02	BP	Down	1
GO:0006561	proline biosynthetic process	0.03	BP	Down	1
GO:0006562	proline catabolic process	0.03	BP	Down	1
GO:0006654	phosphatidic acid biosynthetic process	0.03	BP	Down	1
GO:0006857	oligopeptide transport	0.01	BP	Down	1
GO:0007229	integrin-mediated signaling pathway	0	BP	Down	1
GO:0009094	L-phenylalanine biosynthetic process	0.03	BP	Down	1
GO:0009168	purine ribonucleoside monophosphate biosynthetic process	0.02	BP	Down	1
GO:0015689	molybdate ion transport	0.02	BP	Down	1
GO:0019379	sulfate assimilation, phosphoadenylyl sulfate reduction by phosphoadenylyl-sulfate reductase (thioredoxin)	0.04	BP	Down	1
GO:0019500	cyanide catabolic process	0.02	BP	Down	1
GO:0019629	propionate catabolic process, 2-methylcitrate cycle	0.04	BP	Down	1
GO:0033609	oxalate metabolic process	0.02	BP	Down	1
GO:0035835	indole alkaloid biosynthetic process	0.04	BP	Down	1
GO:0045132	meiotic chromosome segregation	0.04	BP	Down	1
GO:0045901	positive regulation of translational elongation	0.03	BP	Down	1
GO:0048017	inositol lipid-mediated signaling	0.04	BP	Down	1
GO:0048312	intracellular distribution of mitochondria	0.01	BP	Down	1
GO:0051208	sequestering of calcium ion	0.02	BP	Down	1
GO:0051259	protein complex oligomerization	0.02	BP	Down	1
GO:0071596	ubiquitin-dependent protein catabolic process via the N-end rule pathway	0.04	BP	Down	1
GO:0071963	establishment or maintenance of cell polarity regulating cell shape	0.04	BP	Down	1
GO:0097623	potassium ion export across plasma membrane	0.03	BP	Down	1

GO:0098656	anion transmembrane transport	0.03	BP	Down	1
GO:0098863	nuclear migration by microtubule mediated pushing forces	0.03	BP	Down	1
GO:0120029	proton export across plasma membrane	0.02	BP	Down	1
GO:1903087	mitotic spindle pole body duplication	0.03	BP	Down	1
GO:1903474	anchoring of the mitotic actomyosin contractile ring to the plasma membrane	0.04	BP	Down	1
GO:0006508	proteolysis	0.02	BP	Up	7
GO:0008150	biological_process	0.01	BP	Up	7
GO:0009405	pathogenesis	0.02	BP	Up	3
GO:0043386	mycotoxin biosynthetic process	0	BP	Up	3
GO:0009116	nucleoside metabolic process	0.02	BP	Up	2
GO:0046677	response to antibiotic	0	BP	Up	2
GO:0051607	defense response to virus	0	BP	Up	2
GO:0006048	UDP-N-acetylglucosamine biosynthetic process	0.03	BP	Up	1
GO:0006139	nucleobase-containing compound metabolic process	0.03	BP	Up	1
GO:0006189	'de novo' IMP biosynthetic process	0.04	BP	Up	1
GO:0006334	nucleosome assembly	0.03	BP	Up	1
GO:0006438	valyl-tRNA aminoacylation	0	BP	Up	1
GO:0006526	arginine biosynthetic process	0.04	BP	Up	1
GO:0006689	ganglioside catabolic process	0.01	BP	Up	1
GO:0007131	reciprocal meiotic recombination	0	BP	Up	1
GO:0009313	oligosaccharide catabolic process	0.02	BP	Up	1
GO:0009820	alkaloid metabolic process	0.02	BP	Up	1
GO:0009972	cytidine deamination	0	BP	Up	1
GO:0015940	pantothenate biosynthetic process	0.02	BP	Up	1
GO:0016117	carotenoid biosynthetic process	0.03	BP	Up	1
GO:0017001	antibiotic catabolic process	0.01	BP	Up	1
GO:0019441	tryptophan catabolic process to kynurenine	0.01	BP	Up	1
GO:0030091	protein repair	0.01	BP	Up	1
GO:0030435	sporulation resulting in formation of a cellular spore	0	BP	Up	1
GO:0044550	secondary metabolite biosynthetic process	0	BP	Up	1
GO:0046416	D-amino acid metabolic process	0.02	BP	Up	1
GO:0050688	regulation of defense response to virus	0	BP	Up	1

24 hours (Keratin)

**Go term**	**Description**	**p-value**	**Class**	**Regulation**	**Genes**

GO:0055085	transmembrane transport	0	BP	Down	22
GO:0005975	carbohydrate metabolic process	0	BP	Down	14
GO:0006357	regulation of transcription by RNA polymerase II	0.03	BP	Down	12
GO:0008150	biological_process	0.01	BP	Down	12
GO:0008152	metabolic process	0	BP	Down	12
GO:0006351	transcription, DNA-templated	0	BP	Down	9
GO:0000272	polysaccharide catabolic process	0	BP	Down	4
GO:0006807	nitrogen compound metabolic process	0.01	BP	Down	3
GO:0019441	tryptophan catabolic process to kynurenine	0	BP	Down	3
GO:0030001	metal ion transport	0.03	BP	Down	3
GO:0006032	chitin catabolic process	0.01	BP	Down	2
GO:0006044	N-acetylglucosamine metabolic process	0	BP	Down	2
GO:0006801	superoxide metabolic process	0.02	BP	Down	2
GO:0016042	lipid catabolic process	0.04	BP	Down	2
GO:0019752	carboxylic acid metabolic process	0	BP	Down	2
GO:0070941	eisosome assembly	0	BP	Down	2
GO:0005976	polysaccharide metabolic process	0.02	BP	Down	1
GO:0006190	inosine salvage	0.02	BP	Down	1
GO:0006428	isoleucyl-tRNA aminoacylation	0.03	BP	Down	1
GO:0006542	glutamine biosynthetic process	0.03	BP	Down	1
GO:0006571	tyrosine biosynthetic process	0.04	BP	Down	1
GO:0007155	cell adhesion	0.01	BP	Down	1
GO:0009072	aromatic amino acid family metabolic process	0.03	BP	Down	1
GO:0009094	L-phenylalanine biosynthetic process	0.03	BP	Down	1
GO:0009168	purine ribonucleoside monophosphate biosynthetic process	0.01	BP	Down	1
GO:0010469	regulation of signaling receptor activity	0.02	BP	Down	1
GO:0015680	intracellular copper ion transport	0.01	BP	Down	1
GO:0016052	carbohydrate catabolic process	0.03	BP	Down	1
GO:0018344	protein geranylgeranylation	0	BP	Down	1
GO:0019629	propionate catabolic process, 2-methylcitrate cycle	0.02	BP	Down	1
GO:0023014	signal transduction by protein phosphorylation	0.04	BP	Down	1
GO:0030261	chromosome condensation	0.02	BP	Down	1
GO:0031408	oxylipin biosynthetic process	0.03	BP	Down	1
GO:0032264	IMP salvage	0.02	BP	Down	1
GO:0042128	nitrate assimilation	0.04	BP	Down	1
GO:0043085	positive regulation of catalytic activity	0.04	BP	Down	1
GO:0045461	sterigmatocystin biosynthetic process	0.03	BP	Down	1

GO:0046470	phosphatidylcholine metabolic process	0	BP	Down	1
GO:0051259	protein complex oligomerization	0.03	BP	Down	1
GO:0071592	nicotinic acid riboside biosynthetic process	0.04	BP	Down	1
GO:0097428	protein maturation by iron-sulfur cluster transfer	0	BP	Down	1
GO:0055114	oxidation-reduction process	0	BP	Up	38
GO:0055085	transmembrane transport	0	BP	Up	17
GO:0000226	microtubule cytoskeleton organization	0	BP	Up	3
GO:0007020	microtubule nucleation	0	BP	Up	3
GO:0043386	mycotoxin biosynthetic process	0	BP	Up	3
GO:0006096	glycolytic process	0.02	BP	Up	2
GO:0006464	cellular protein modification process	0	BP	Up	2
GO:0006694	steroid biosynthetic process	0	BP	Up	2
GO:0008610	lipid biosynthetic process	0	BP	Up	2
GO:0098869	cellular oxidant detoxification	0.02	BP	Up	2
GO:0000184	nuclear-transcribed mRNA catabolic process, nonsense-mediated decay	0.04	BP	Up	1
GO:0006006	glucose metabolic process	0.01	BP	Up	1
GO:0006048	UDP-N-acetylglucosamine biosynthetic process	0.01	BP	Up	1
GO:0006098	pentose-phosphate shunt	0.01	BP	Up	1
GO:0006535	cysteine biosynthetic process from serine	0.01	BP	Up	1
GO:0006537	glutamate biosynthetic process	0.02	BP	Up	1
GO:0006560	proline metabolic process	0	BP	Up	1
GO:0006561	proline biosynthetic process	0.01	BP	Up	1
GO:0006562	proline catabolic process	0.02	BP	Up	1
GO:0006564	L-serine biosynthetic process	0.01	BP	Up	1
GO:0006568	tryptophan metabolic process	0.03	BP	Up	1
GO:0008202	steroid metabolic process	0.02	BP	Up	1
GO:0015696	ammonium transport	0.01	BP	Up	1
GO:0017001	antibiotic catabolic process	0.03	BP	Up	1
GO:0019343	cysteine biosynthetic process via cystathionine	0.01	BP	Up	1
GO:0019344	cysteine biosynthetic process	0.03	BP	Up	1
GO:0019441	tryptophan catabolic process to kynurenine	0.04	BP	Up	1
GO:0032781	positive regulation of ATPase activity	0.02	BP	Up	1
GO:0034220	ion transmembrane transport	0.03	BP	Up	1
GO:0044550	secondary metabolite biosynthetic process	0.01	BP	Up	1
GO:0044571	[2Fe-2S] cluster assembly	0	BP	Up	1
GO:0045332	phospholipid translocation	0.04	BP	Up	1

GO:0045493	xylan catabolic process	0	BP	Up	1
GO:0051382	kinetochore assembly	0.04	BP	Up	1
GO:0072488	ammonium transmembrane transport	0.01	BP	Up	1
GO:0120113	protein localization by the NVT pathway	0.01	BP	Up	1

48 hours (Keratin)

Go term	Description	p-value	Class	Regulation	Genes
GO:0055114	oxidation-reduction process	0	BP	Down	51
GO:0055085	transmembrane transport	0	BP	Down	32
GO:0008150	biological_process	0.01	BP	Down	17
GO:0005975	carbohydrate metabolic process	0	BP	Down	14
GO:0008152	metabolic process	0	BP	Down	10
GO:0098869	cellular oxidant detoxification	0	BP	Down	5
GO:0000272	polysaccharide catabolic process	0	BP	Down	4
GO:0006979	response to oxidative stress	0	BP	Down	4
GO:0019752	carboxylic acid metabolic process	0	BP	Down	4
GO:0006032	chitin catabolic process	0	BP	Down	3
GO:0006807	nitrogen compound metabolic process	0.02	BP	Down	3
GO:0043386	mycotoxin biosynthetic process	0	BP	Down	3
GO:0070941	eisosome assembly	0	BP	Down	3
GO:0001678	cellular glucose homeostasis	0	BP	Down	2
GO:0006044	N-acetylglucosamine metabolic process	0	BP	Down	2
GO:0006801	superoxide metabolic process	0	BP	Down	2
GO:0031408	oxylipin biosynthetic process	0.01	BP	Down	2
GO:0000737	DNA catabolic process, endonucleolytic	0.04	BP	Down	1
GO:0005976	polysaccharide metabolic process	0.04	BP	Down	1
GO:0005991	trehalose metabolic process	0.02	BP	Down	1
GO:0006012	galactose metabolic process	0.03	BP	Down	1
GO:0006097	glyoxylate cycle	0.04	BP	Down	1
GO:0006190	inosine salvage	0.04	BP	Down	1
GO:0006421	asparaginyl-tRNA aminoacylation	0.03	BP	Down	1
GO:0006437	tyrosyl-tRNA aminoacylation	0.03	BP	Down	1
GO:0006797	polyphosphate metabolic process	0.04	BP	Down	1
GO:0006798	polyphosphate catabolic process	0.03	BP	Down	1
GO:0009063	cellular amino acid catabolic process	0.04	BP	Down	1
GO:0015680	intracellular copper ion transport	0.02	BP	Down	1
GO:0019594	mannitol metabolic process	0.02	BP	Down	1

GO:0032264	IMP salvage	0.03	BP	Down	1
GO:0046470	phosphatidylcholine metabolic process	0.01	BP	Down	1
GO:0046952	ketone body catabolic process	0.02	BP	Down	1
GO:0050482	arachidonic acid secretion	0.01	BP	Down	1
GO:0051259	protein complex oligomerization	0.01	BP	Down	1
GO:0071590	nicotinamide riboside biosynthetic process	0.04	BP	Down	1
GO:0055114	oxidation-reduction process	0	BP	Up	41
GO:0055085	transmembrane transport	0	BP	Up	29
GO:0032259	methylation	0.04	BP	Up	12
GO:0006364	rRNA processing	0	BP	Up	9
GO:0042254	ribosome biogenesis	0	BP	Up	8
GO:0000226	microtubule cytoskeleton organization	0	BP	Up	3
GO:0007020	microtubule nucleation	0.01	BP	Up	3
GO:0008610	lipid biosynthetic process	0	BP	Up	3
GO:0043386	mycotoxin biosynthetic process	0	BP	Up	3
GO:0006094	gluconeogenesis	0	BP	Up	2
GO:0006561	proline biosynthetic process	0.01	BP	Up	2
GO:0006816	calcium ion transport	0.01	BP	Up	2
GO:0008299	isoprenoid biosynthetic process	0.01	BP	Up	2
GO:0015936	coenzyme A metabolic process	0	BP	Up	2
GO:0019441	tryptophan catabolic process to kynurenine	0.01	BP	Up	2
GO:0031145	anaphase-promoting complex-dependent catabolic process	0.02	BP	Up	2
GO:0070588	calcium ion transmembrane transport	0.04	BP	Up	2
GO:0006144	purine nucleobase metabolic process	0.01	BP	Up	1
GO:0006177	GMP biosynthetic process	0.03	BP	Up	1
GO:0006516	glycoprotein catabolic process	0.03	BP	Up	1
GO:0006517	protein deglycosylation	0.04	BP	Up	1
GO:0006817	phosphate ion transport	0.02	BP	Up	1
GO:0007531	mating type determination	0.04	BP	Up	1
GO:0008612	peptidyl-lysine modification to peptidyl-hypusine	0.03	BP	Up	1
GO:0010133	proline catabolic process to glutamate	0.04	BP	Up	1
GO:0015696	ammonium transport	0.01	BP	Up	1
GO:0017001	antibiotic catabolic process	0.03	BP	Up	1
GO:0019628	urate catabolic process	0.01	BP	Up	1
GO:0032933	SREBP signaling pathway	0.03	BP	Up	1
GO:0034217	ascospore wall chitin biosynthetic process	0.02	BP	Up	1

GO:0034356	NAD biosynthesis via nicotinamide riboside salvage pathway	0.03	BP	Up	1
GO:0036003	positive regulation of transcription from RNA polymerase II promoter in response to stress	0.04	BP	Up	1
GO:0044571	[2Fe-2S] cluster assembly	0.03	BP	Up	1
GO:0045895	positive regulation of mating-type specific transcription, DNA-templated	0.03	BP	Up	1
GO:0051570	regulation of histone H3-K9 methylation	0.01	BP	Up	1
GO:0055129	L-proline biosynthetic process	0.02	BP	Up	1
GO:0060548	negative regulation of cell death	0.04	BP	Up	1
GO:0070125	mitochondrial translational elongation	0.03	BP	Up	1
GO:0070868	heterochromatin organization involved in chromatin silencing	0.02	BP	Up	1
GO:0070919	production of siRNA involved in chromatin silencing by small RNA	0.04	BP	Up	1
GO:0071046	nuclear polyadenylation-dependent ncRNA catabolic process	0.03	BP	Up	1
GO:0071409	cellular response to cycloheximide	0.03	BP	Up	1
GO:0072488	ammonium transmembrane transport	0.04	BP	Up	1
GO:0120113	protein localization by the NVT pathway	0.01	BP	Up	1
GO:1903474	anchoring of the mitotic actomyosin contractile ring to the plasma membrane	0.02	BP	Up	1

96 hours (Keratin)

Go term	Description	p-value	Class	Regulation	Genes
GO:0055114	oxidation-reduction process	0	BP	Down	60
GO:0055085	transmembrane transport	0	BP	Down	40
GO:0008150	biological_process	0.03	BP	Down	19
GO:0005975	carbohydrate metabolic process	0	BP	Down	18
GO:0008152	metabolic process	0	BP	Down	17
GO:0098869	cellular oxidant detoxification	0	BP	Down	7
GO:0006979	response to oxidative stress	0	BP	Down	6
GO:0000272	polysaccharide catabolic process	0.01	BP	Down	4
GO:0006099	tricarboxylic acid cycle	0.03	BP	Down	4
GO:0019752	carboxylic acid metabolic process	0.01	BP	Down	4
GO:0019441	tryptophan catabolic process to kynurenine	0.01	BP	Down	3
GO:0031408	oxylipin biosynthetic process	0	BP	Down	3
GO:0043386	mycotoxin biosynthetic process	0.02	BP	Down	3
GO:0001678	cellular glucose homeostasis	0.01	BP	Down	2

GO:0006032	chitin catabolic process	0.04	BP	Down	2
GO:0006097	glyoxylate cycle	0	BP	Down	2
GO:0016558	protein import into peroxisome matrix	0	BP	Down	2
GO:0016560	protein import into peroxisome matrix, docking	0.01	BP	Down	2
GO:0030259	lipid glycosylation	0	BP	Down	2
GO:0070941	eisosome assembly	0.02	BP	Down	2
GO:0001315	age-dependent response to reactive oxygen species	0.02	BP	Down	1
GO:0005976	polysaccharide metabolic process	0.03	BP	Down	1
GO:0006689	ganglioside catabolic process	0.02	BP	Down	1
GO:0009310	amine catabolic process	0.02	BP	Down	1
GO:0009313	oligosaccharide catabolic process	0.04	BP	Down	1
GO:0015833	peptide transport	0.02	BP	Down	1
GO:0019499	cyanide metabolic process	0.03	BP	Down	1
GO:0019500	cyanide catabolic process	0.03	BP	Down	1
GO:0019748	secondary metabolic process	0.04	BP	Down	1
GO:0030242	autophagy of peroxisome	0.03	BP	Down	1
GO:0032264	IMP salvage	0.04	BP	Down	1
GO:0071590	nicotinamide riboside biosynthetic process	0.01	BP	Down	1
GO:0055114	oxidation-reduction process	0	BP	Up	47
GO:0005975	carbohydrate metabolic process	0.04	BP	Up	9
GO:0006457	protein folding	0	BP	Up	9
GO:0006364	rRNA processing	0	BP	Up	7
GO:0006633	fatty acid biosynthetic process	0	BP	Up	5
GO:0008610	lipid biosynthetic process	0	BP	Up	5
GO:0006414	translational elongation	0.02	BP	Up	4
GO:0000226	microtubule cytoskeleton organization	0.01	BP	Up	3
GO:0000413	protein peptidyl-prolyl isomerization	0.03	BP	Up	3
GO:0006541	glutamine metabolic process	0.02	BP	Up	3
GO:0007018	microtubule-based movement	0.01	BP	Up	3
GO:0007020	microtubule nucleation	0	BP	Up	3
GO:0016126	sterol biosynthetic process	0	BP	Up	3
GO:0031505	fungal-type cell wall organization	0	BP	Up	3
GO:0042026	protein refolding	0	BP	Up	3
GO:0071555	cell wall organization	0	BP	Up	3
GO:0099132	ATP hydrolysis coupled cation transmembrane transport	0.02	BP	Up	3
GO:0000054	ribosomal subunit export from nucleus	0.01	BP	Up	2

GO:0005978	glycogen biosynthetic process	0	BP	Up	2
GO:0006006	glucose metabolic process	0	BP	Up	2
GO:0006032	chitin catabolic process	0.03	BP	Up	2
GO:0006037	cell wall chitin metabolic process	0.01	BP	Up	2
GO:0006207	'de novo' pyrimidine nucleobase biosynthetic process	0.04	BP	Up	2
GO:0006334	nucleosome assembly	0.01	BP	Up	2
GO:0006450	regulation of translational fidelity	0.02	BP	Up	2
GO:0006452	translational frameshifting	0	BP	Up	2
GO:0006515	protein quality control for misfolded or incompletely synthesized proteins	0.01	BP	Up	2
GO:0006696	ergosterol biosynthetic process	0.01	BP	Up	2
GO:0008202	steroid metabolic process	0.02	BP	Up	2
GO:0031145	anaphase-promoting complex-dependent catabolic process	0.03	BP	Up	2
GO:0042759	long-chain fatty acid biosynthetic process	0	BP	Up	2
GO:0051083	'de novo' cotranslational protein folding	0	BP	Up	2
GO:0070125	mitochondrial translational elongation	0	BP	Up	2
GO:0000464	endonucleolytic cleavage in ITS1 upstream of 5.8S rRNA from tricistronic rRNA transcript (SSU-rRNA, 5.8S rRNA, LSU-rRNA)	0.02	BP	Up	1
GO:0000465	exonucleolytic trimming to generate mature 5'-end of 5.8S rRNA from tricistronic rRNA transcript (SSU-rRNA, 5.8S rRNA, LSU-rRNA)	0.02	BP	Up	1
GO:0000959	mitochondrial RNA metabolic process	0.04	BP	Up	1
GO:0006177	GMP biosynthetic process	0.04	BP	Up	1
GO:0006430	lysyl-tRNA aminoacylation	0.04	BP	Up	1
GO:0006437	tyrosyl-tRNA aminoacylation	0.04	BP	Up	1
GO:0006516	glycoprotein catabolic process	0.03	BP	Up	1
GO:0006517	protein deglycosylation	0.04	BP	Up	1
GO:0006561	proline biosynthetic process	0.02	BP	Up	1
GO:0006616	SRP-dependent cotranslational protein targeting to membrane, translocation	0.03	BP	Up	1
GO:0006817	phosphate ion transport	0.02	BP	Up	1
GO:0008615	pyridoxine biosynthetic process	0.02	BP	Up	1
GO:0018293	protein-FAD linkage	0.04	BP	Up	1
GO:0019538	protein metabolic process	0.01	BP	Up	1
GO:0019551	glutamate catabolic process to 2-oxoglutarate	0.02	BP	Up	1
GO:0019941	modification-dependent protein catabolic process	0.01	BP	Up	1
GO:0031322	ascospore-type prospore-specific spindle pole body remodeling	0.02	BP	Up	1

GO:0032509	endosome transport via multivesicular body sorting pathway	0.04	BP	Up	1
GO:0032933	SREBP signaling pathway	0.03	BP	Up	1
GO:0032949	regulation of alpha-glucan biosynthetic process	0.04	BP	Up	1
GO:0032979	protein insertion into mitochondrial membrane from inner side	0.03	BP	Up	1
GO:0035719	tRNA import into nucleus	0.02	BP	Up	1
GO:0036003	positive regulation of transcription from RNA polymerase II promoter in response to stress	0.02	BP	Up	1
GO:0043335	protein unfolding	0.02	BP	Up	1
GO:0045984	negative regulation of pyrimidine nucleobase metabolic process	0.02	BP	Up	1
GO:0060003	copper ion export	0.03	BP	Up	1
GO:0060548	negative regulation of cell death	0.02	BP	Up	1
GO:0070370	cellular heat acclimation	0.03	BP	Up	1
GO:0070475	rRNA base methylation	0.02	BP	Up	1
GO:0071409	cellular response to cycloheximide	0.04	BP	Up	1
GO:0071630	nuclear protein quality control by the ubiquitin-proteasome system	0	BP	Up	1
GO:0120113	protein localization by the NVT pathway	0.03	BP	Up	1
GO:0140021	mitochondrial ADP transmembrane transport	0.03	BP	Up	1

Supplementary Table S7. RNA sequencing vs. real-time quantitative reverse transcription polymerase chain reaction (qRT-PCR).

ID	Condition(s)	RNA-Seq	qPCR	Gene Product Name
TERG_02307	24h G	2.35	1.67	mitotic spindle checkpoint protein (Mad2B), putative (*T. verrucosum*)
TERG_11637	24h G	-2.14	-1.21	isocitrate lyase
TERG_07959	24h G / 96h K	2.23 / 3.01	1.95 / 0.96	GPI-anchored cell wall beta-1,3-endoglucanase EglC (*A. benhamiae*)
TERG_06651	48h G / 96h K	-1.62 / -3.29	1.81 / -0.36	cytochrome P450, putative (*T. verrucosum*)
TERG_08114	48h G / 48h K	3.03 / 2.69	0.88 / 0.21	anaphase-promoting complex component Cut20/Apc4 (*T. tonsurans*)
TERG_00060	96h G	3.30	0.33	GPI anchored cell wall protein, putative (*A. benhamiae*)
TERG_00119	96h G	1.61	5.73	CAMK/CAMKL/GIN4 protein kinase
TERG_01840	96h G	3.01	1.90	carboxypeptidase S1 (*T. equinum*)
TERG_01900	96h G / 96h K	-2.21 / 2.47	-2.37 / 1.93	aquaglyceroporin, putative (*A. benhamiae*)
TERG_01901	96h G / 96h K	-2.99 / 2.13	-1.70 / 1.71	glycerol kinase
TERG_02368	96h G / 96h K	-1.98 / -3.15	-0.69 / -2.68	extracellular developmental signal biosynthesis protein FluG (*A. benhamiae*)
TERG_03544	96h G / 96h K	-3.13 / -1.55	-2.25 / -1.64	CMGC/RCK/MAK protein kinase
TERG_04282	96h G / 96h K	-5.57 / -4.05	-5.03 / -3.11	GPI ethanolamine phosphate transferase (*T. tonsurans*)
TERG_08261	96h G / 96h K	-3.75 / -3.83	-0.33 / -1.65	glutamate decarboxylase
TERG_01950	96h K	-3.52	-2.28	serine/threonine protein kinase (*T. tonsurans*)
TERG_05627	96h K	-1.64	-6.69	LysM domain-containing protein (*M. canis*)

Gene expression values are expressed in log_2 fold change.
